# Research on the influence of g-C_3_N_4_ microstructure changes on the efficiency of visible light photocatalytic degradation

**DOI:** 10.1016/j.wroa.2025.100315

**Published:** 2025-02-08

**Authors:** Hong Tu, Bihong Tian, Zhichao Zhao, Renjiang Guo, Ya Wang, Shunhong Chen, Jian Wu

**Affiliations:** State Key Laboratory of Green Pesticides, Key Laboratory of Green Pesticide and Agricultural Bioengineering, Ministry of Education, Guizhou University, Huaxi District, Guiyang 550025, PR China

**Keywords:** DFT calculation, Electron-hole pair, Wastewater contaminants, Visible-light photocatalysis

## Abstract

The structural modulation of pristine graphitic carbon nitride poses a considerable challenge in the rational design of catalysts for the efficient degradation of small organic pollutants under visible light. In this study, we combined first-principles calculations and the structure-function relationship to predict a high-performance catalyst. The results reveal that CN-8 demonstrates a remarkable degree of electron-hole separation. Notably, CN-8 shows exceptional degradation efficiency towards rhodamine B, tetracycline, bisphenol A, and fluralaner under visible light irradiation. Specifically, the degradation rate constants are 11, 4, 12, and 32 times higher, respectively, compared to bulk g-C_3_N_4_. Through density functional theory calculations and investigations of the structure-function relationship, it is confirmed that the superior catalytic activity of CN-8 lies in modifying the amino position, which alters the electron cloud distribution and promotes the efficient separation of photo-generated electron-hole pairs. This study provides valuable insights for the development of eco-friendly and efficient photocatalysts for environmental remediation.

## Highlights

• ·Structurally modulating g-C_3_N_4_ for visible-light degradation of small organic pollutants.
• ·This study combines calculations and relationships to predict a catalyst.
• ·DFT calculations reveal that electron-holes in the modified CN-8 are effectively separated.

## Graphical abstract

**Figure:**
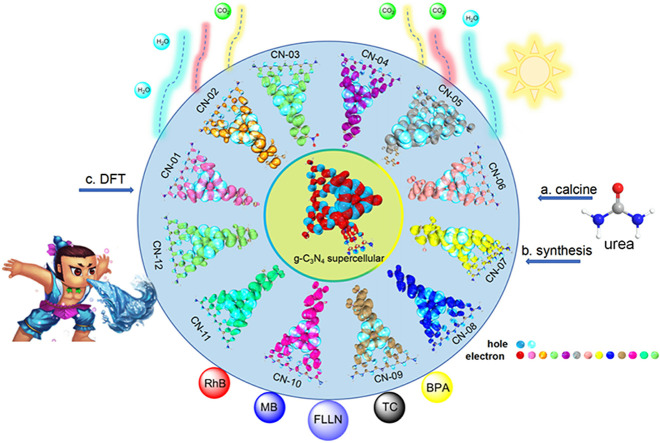

## 1 Introduction

Currently, the growing water pollution problem is mainly caused by industrial sewage discharge, medical antiviral drug use, and agricultural pesticide application(Choudhury, Ojha and Ray, 2024; Letsoalo et al., 2023; Priya et al., 2024). Emerging pollutants like pharmaceutical products (tetracycline (TC))(Chen et al., 2023; Manju et al., 2024), industrial raw materials (bisphenol A (BPA))(Hu et al., 2024; Yang et al., 2024) pesticides, and dyes (rhodamine B (RhB)(Pawariya, De and Dutta, 2024), methylene blue (MB))(Lee and Lee, 2024), in water systems pose a serious threat to the environment and living organisms. Pharmaceutical products like TC, used in human and veterinary medicine, can enter water systems through improper disposal. It can cause antibiotic-resistant bacteria and disrupt microbial balance in ecosystems. BPA, a widely used industrial raw material in plastic and epoxy resin production, can leach into water sources(Michenzi, Myers and Chiarotto, 2024). It's an endocrine-disruptor, linked to various health problems. Insecticide like fluralaner (FLLN) used in agriculture and public health can enter water systems through runoff and leaching(Gastaldi et al., 2023; Tao et al., 2024). FLLN is a broad-spectrum insecticide targeting parasites in animals(Elliot et al., 2024). Its presence in water bodies harms non-target organisms like aquatic insects, honeybees, and amphibians. Pesticides disrupt ecosystem balance and biodiversity. Dyes like RhB and MB, used in industries like textiles, printing, and paper manufacturing, contaminate aquatic environments when their wastewater is discharged(Holkar et al., 2016). Dyes reduce light penetration, affecting photosynthesis and aquatic plant growth. They are also toxic to aquatic organisms, affecting their survival, reproduction, and ecological balance(Chen et al., 2024b; Liu et al., 2024).

Overall, these emerging pollutants in water systems have wide-ranging impacts on the environment, organisms, and human health. It's crucial to develop effective removal and remediation methods to protect water resources(Yuan et al., 2024). Photocatalysis is a promising pollutant degradation strategy, but low sunlight utilization and high charge carrier recombination hinder its efficiency and applications(Qi et al., 2024). Urgently needed are green and sustainable technologies and policies to mitigate or eliminate antibiotic residues and dyes. Stable and non-biodegradable species like RhB, MB, TC, FLLN, and BPA can bioaccumulate and cause adverse effects even in trace amounts. Due to the demand for clean water, water reclamation, recycling, and reuse projects are emerging worldwide.

Semiconductor photocatalysis has great potential in degrading aqueous pollutants(Cheng, Liu and Xiong, 2024; Song, Bao and Gu, 2023). Graphitic carbon nitride (g-C_3_N_4_) is a promising inorganic nonmetal conjugated semiconductor(Bao et al., 2024). Its photocatalytic activity can be enhanced through doping, functionalization, and structural optimization to modify its electronic, surface, and structural properties, improving visible light absorption and charge separation/transfer(Cao et al., 2023; Chen et al., 2024c). This holds potential for efficient photocatalysts in pollutant removal. It has a layered graphite-like structure with triazine or heptazine units. In recent years, g-C_3_N_4_ has drawn significant attention in photocatalysis due to its physicochemical stability, large surface area, and non-toxicity. Crucially, its conduction and valence bands at -1.4 V and 1.3 V make it efficient in visible light absorption, promising for removing pollutants from water(Gupta, Kulkarni and Ahmaruzzaman, 2024).

**Fig. 1:**
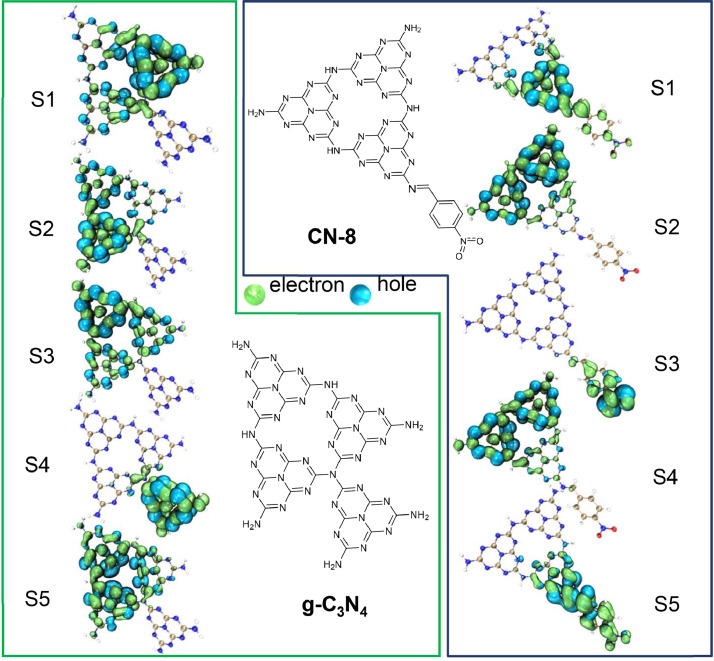
The electron-holes distribution of the first to fifth excited states in g-C_3_N_4_ and CN-8 calculated by DFT calculations.

In this study, we proposed a method to modify the electronic cloud density distribution of the organic conjugated framework via amino modification, facilitating the separation of photo-generated charge carriers. By introducing organic small molecules with benzaldehyde to pristine g-C_3_N_4_, the resulting imide structure showed an expanded shift in electron cloud density. Previous research modified the amino group successfully to enhance photocatalytic activity. Based on theoretical analysis and literature, we investigated the influence of substituents on the electron cloud density above the benzene ring. Introducing a strong electron-withdrawing group enhanced the effective separation of electrons and holes. Our team used Density Functional Theory (DFT) to compute the electron-holes distribution patterns of twelve unique functional groups and S1-S5 of CN-8 and g-C_3_N_4_ supercell (Fig. 1)(Liu, Lu and Chen, 2020; Lu and Chen, 2011; Tu et al., 2024). This effectively enhances pi-pi bond conjugation and increases the separation distance of electron-hole pairs, significantly enhancing photocatalytic activity. This study is the first to reliably verify the structure-function relationship by calculating electron-holes using DFT(Zhang and Lu, 2021).

## 2 Results and discussion

### 2.1 Preparation and characterization of photocatalysts

**Fig. 2:**
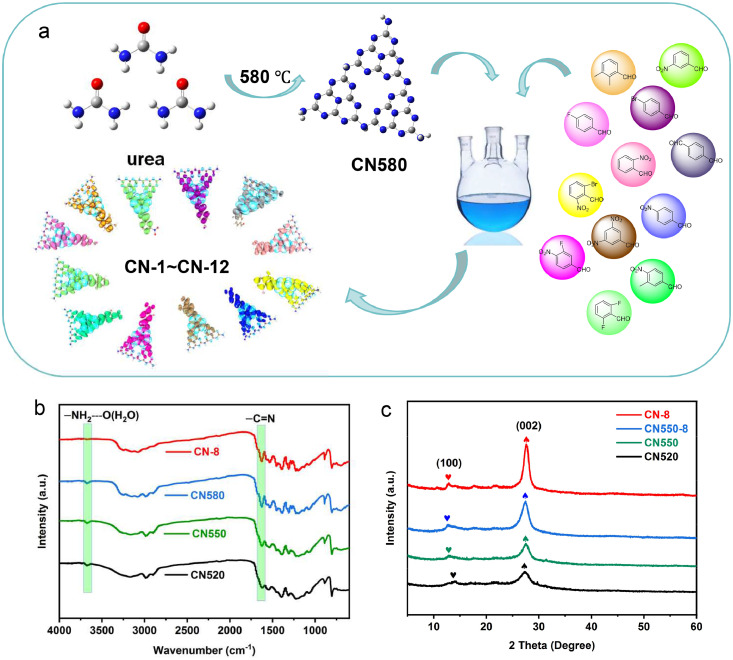
(a) Illustration of synthetic routes of CN-1∼CN-12; (b) FT-IR for CN520, CN550, CN580 and CN-8; (c) XRD for CN520, CN550, CN550–8 and CN-8.

To prepare a highly active CN-8 catalyst with enhanced light absorption, a temperature gradient selection method was used in the synthesis of g-C_3_N_4_ (Fig. 2**a**). It was found that temperatures below 520 °C led to insufficient urea polymerization, and higher temperatures (590, 600, 610 °C) led to low yields. Thus, g-C_3_N_4_ precursor materials were prepared at 520, 550 (bulk g-C_3_N_4_), and 580 °C. Infrared spectroscopy showed an increase in —C=N (1660–1680 cm^-1^) bonds with rising temperature (Fig. 2**b**)(Hemmati-Eslamlu and Habibi-Yangjeh, 2024). Among the synthesized materials, CN580 had the best degradation activity towards RhB under visible light at 450 nm and was further modified with 12 different benzaldehyde derivatives connected to the phenyl ring, resulting in the synthesis of CN-1 to CN-12.

To analyze the crystal structure of CN520, CN550, CN580, and CN-8, their X-ray powder diffraction (XRD) patterns were examined (Fig. 2**c**)(Mafa et al., 2023a). These patterns showed two distinct diffraction peaks at 13.1° and 27.3°, corresponding to the (100) in-plane long-range atomic order and (002) interlayer-stacking motif respectively. As the calcination temperature of g-C_3_N_4_ rose, the intensity of the (002) peak increased, indicating a better stacking order of the interlayers(Malefane et al., 2023). This means higher calcination temperatures promote a more ordered crystal structure. Moreover, when comparing the modified sample (CN550–8) to CN550, a significant increase in peak intensity was observed. This indicates that the modification with electron-donating groups at the amino position (CN-8) improves the crystallinity of the material, resulting in a more organized and stable structure. This enhancement in the crystal structure may contribute to the better photocatalytic performance in the degradation experiments. Overall, these XRD results strongly prove the structural changes caused by the modification of g-C_3_N_4_ and highlight the potential of these modified materials for various applications. After modifying CN580 with p-nitrobenzaldehyde to obtain CN-8, the infrared spectra showed the disappearance of the absorption peak of hydrogen bonds formed by amino groups (3620–3680 cm^-1^). Density functional theory (DFT) calculations, combined with experimental screening, identified CN-8 as the most suitable catalyst.

The SEM images in **Figure S1** and **Figure S2** and **Figure S3a-b** (Supporting Information) indicate that at low magnification, CN520, CN550, CN580, and CN-1∼CN-12 as a whole exhibits an aggregated morphology of lumps or layers. These lumpy or lamellar structures are intertwined and stacked, forming a relatively complex and irregular macroscopic profile, much like pieces of paper that have been crumpled and then stacked together. HRTEM images (**Figure S3c-d**) show they have a transparent sheet-like morphology. However, the selected area electron diffraction (SAED) pattern (**Figure S3c, inset)** shows no lattice fringes, suggesting that CN-8 is amorphous(Chen et al., 2024a; Malefane et al., 2024).

To verify the structural change in CN520, CN550, CN550–8, and CN-8, Fourier transform infrared (FTIR) spectroscopy was carried out (Fig. 2**b**). The absorption peak of the —C=N bond (1620–1680 cm^-1^) rises with the increase of the calcination temperature of g-C_3_N_4_. The —NH_2_ stretching vibration peak of the N—H bond and hydrogen bonds between molecules are at 3620–3680 cm^-1^ for CN520, CN550, and CN580. The disappearance of this absorption peak in the CN-8 sample indicates the successful modification of the amino group. These peaks show that benzaldehyde-type compounds have been successfully introduced into the framework of g-C_3_N_4_.

**Fig. 3:**
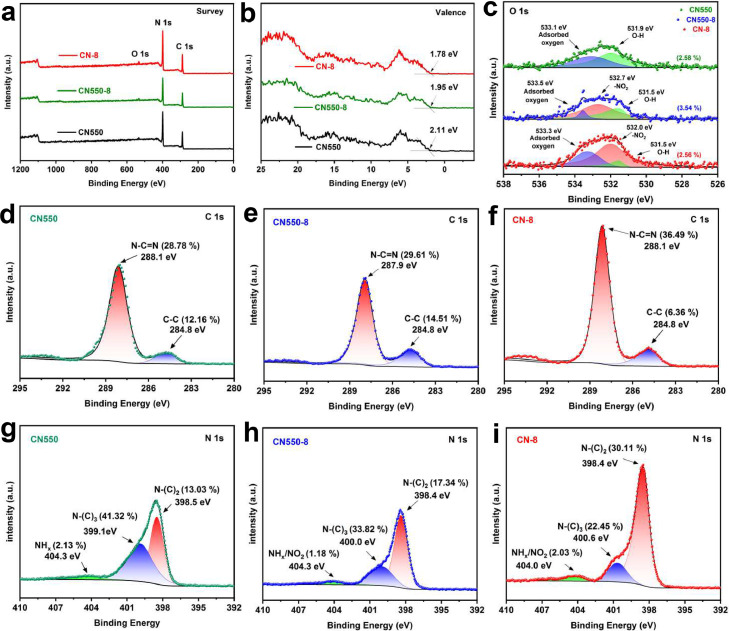
(a) XPS survey for,CN550, CN550–8, and CN-8; (b) XPS valence for CN550, CN550–8, and CN-8; (c-i) XPS of O 1s (c), C 1s (d-f), and N 1s (g-i).

To better understand the structure and surface chemical properties of CN-8, X-ray photoelectron spectroscopy (XPS) characterizations were conducted (Fig. 3**a-b**). Both the XPS survey spectrum and the O 1s spectrum with a binding energy at 532.6 eV indicate the introduction of p-nitrobenzaldehyde (Fig. 3**c**). As shown in **Supplementary Table 1**, the C 1s (Fig. 3**d-f**) and N 1s (Fig. 3**g-i**) spectra show characteristic peaks of C and N heterocycle frameworks. Specifically, the N=C—N, N—(C)_2_, and N—(C)_3_ peaks are observed at 288.1, 398.4, and 400.6 eV respectively. For CN-8, the higher binding energies of the C 1s, N 1s, and O 1s are related to the structural change modified by the p-nitrobenzaldehyde. Notably, the integrated peak area ratio N-(C)2 in N 1s significantly increases from 13.03 % (CN550) to 22.45 % (CN-8)(Seifikar and Habibi-Yangjeh, 2024).

From **Figure S4**, it is clear that the surface elemental bands of CN-8 change significantly under dark and light conditions. In the C 1s spectrum, the C-C peak at 284 eV increases from 56.4% to 62.5%, while the N-C=C content drops from 12.3% to 1.8%. In the N 1s spectrum, the N-(C)_2_ peak rises from 10% to 16.2%, and N-(C)_3_ increases from 4.2% to 5.5%. The O 1s spectrum shows a decrease in -NO_2_ content from 15.7% to 12.6%. Moreover, the valence band decreases from 1.06 eV under dark conditions to 0.62 eV. These results suggest significant surface modifications and potential improvements in the photocatalytic performance of CN-8.

**Fig. 4:**
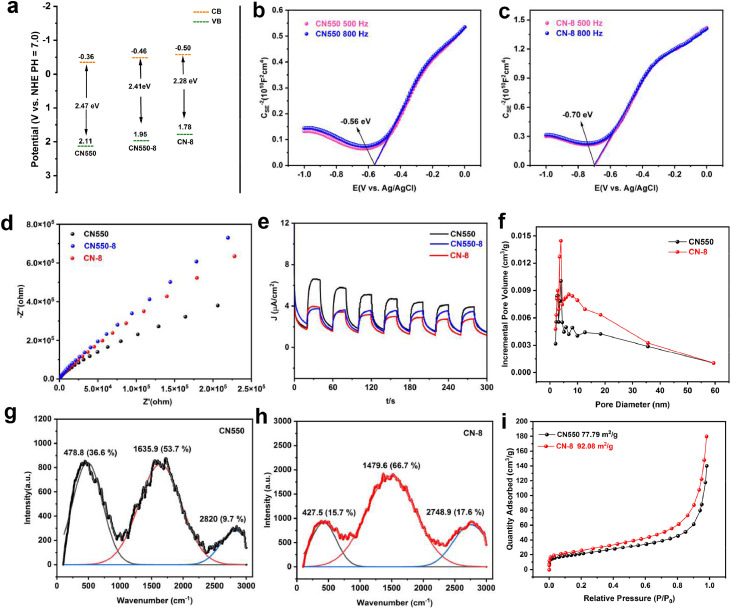
(a) Band structure alignments; (b-c) Mott-Schottky plots of CN550 and CN-8; The potential values in these figures can be converted to potential relative to the standard hydrogen electrode (NHE) using the formula:.

The amino modification and variation in calcination temperature significantly affect the optical properties and light absorption capabilities. UV–vis DRS shows that CN-8 has a stronger and red-shifted absorption capacity compared to CN550 (**Figure S5a**)(Salmanzadeh-Jamadi et al., 2023). From XPS, the valence band (VB) edge potentials of CN550 and CN-8 are estimated as 2.11 and 1.78 eV respectively, establishing the band structure alignments in Fig. 4**a**. The calculated Kubelka-Munk function reveals a progressively narrowed, direct bandgap from 2.82 eV in CN550 to 2.65 eV in CN-8 (**Figure S5b**). The flat band potentials estimated from the measured Mott-Schottky plots are directly used as the conduction band (CB) edge potentials (Fig. 4**b-c, Figure S6**). Charge transfer and separation in CN-8 are further clarified through steady-state and time-dependent photoluminescence (PL) measurements. The PL spectra confirm a red-shift in CN-8, indicating a reduced bandgap(Mafa et al., 2023b).

E (NHE) = E (Ag/AgCl) + 0.197 V; (d) Nyquist plots periodic of CN550 and CN-8 from Electrochemical impedance spectroscopy; (e) ON/OFF photocurrent response; (f) The BJH pore size distributions and (i) N_2_ adsorption isotherms of CN550 and CN-8; (g-h) Raman spectrum of CN550 and CN-8.

**Fig. 5:**
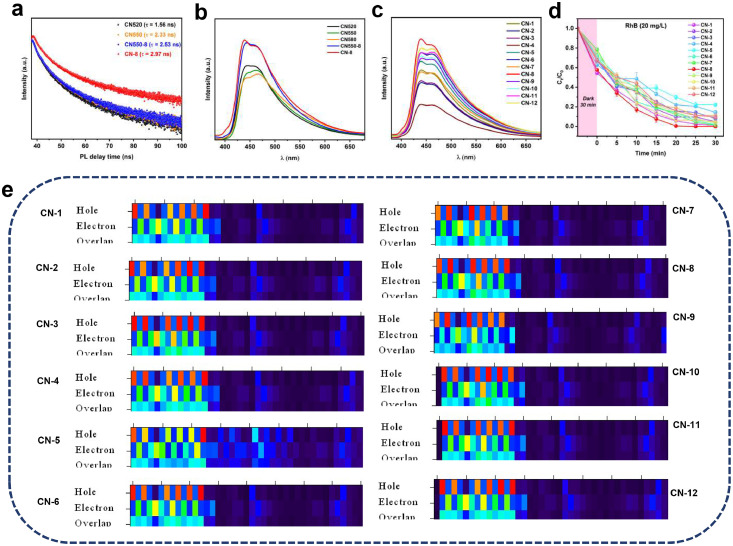
(a) Time-resolved photoluminescence spectra of CN520, CN550, CN550–8, and CN-8; (b) Steady-state PL spectra of CN520,CN550, CN580, CN550–8; (c) Steady-state PL spectra of CN-1∼CN12; (d) Time curves of degradation of RhB by CN-1∼CN-12 under the irradiation of a 6 W LED lamp at λ = 450 nm; (e) The chemical structures and S1 electron-holes distribution hot-maps of CN-1∼CN12.

The photocatalytic performances of CN550 and CN-8 were investigated using electrochemical impedance spectroscopy (EIS) (Fig. 4**d**). By comparing CN-8 to CN550 (Fig. 4**e**), it indicates its weak electron transfer ability and efficient separation of photogenerated carriers for photocatalysis. It can be observed from the graph that the differences in amino modification and calcination temperature led to changes in the electron transfer pathways, thus affecting the intensity of the photoresponse signal. The CN-8 catalysts have much higher specific surface areas and porosities than CN550, up to 92.07 m^2^g^−1^ and 0.2785 cm^3^g^−1^, which is beneficial for facilitating mass diffusion kinetics during catalysis. The Raman spectroscopy test shows that CN-8 has a broader peak width and relatively lower peak position, indicating the presence of lattice defects and distortions in its structure(Zhou et al., 2024). In contrast, CN550 has higher peak positions and narrower peak widths, suggesting a more intact and stable lattice structure (Fig. 4**g-h**). By fitting a double exponential equation, it was found that the electron/hole recombination lifetimes of CN520 were relatively short, but after high-temperature calcination and modification with dinitrobenzaldehyde at the amino sites, the electron/hole recombination lifetimes of the material were significantly improved. Considering the longer PL lifetime, CN-8 was regarded as the optimal choice (Fig. 5**a-b**).

### 2.2 DFT calculation

To have a deeper understanding of the relationship between amino modification and the distribution of electrons and holes, we carried out first-principle density functional theory (DFT) calculations. After optimizing the structure and conducting single-point energy calculations, we analyzed the energy of the first excited state. By plotting the distribution of electrons and holes in the first excited state and comparing it with the experimental rates of RhB photocatalytic degradation (Fig. 5**c-e; Table S3∼S5**), we noticed that higher proportions of electrons and holes on the modified fragments corresponded to higher catalytic rates. Among them, CN-8 and CN-9 had the highest proportion of electron holes. We further explored the bond lengths between N and O in the modified fragments (**Figure S7**) and found that CN-8 had longer bonds, facilitating better separation of electrons and holes (**Figure S8**).

### 2.3 Photocatalytic degradation test

**Fig. 6:**
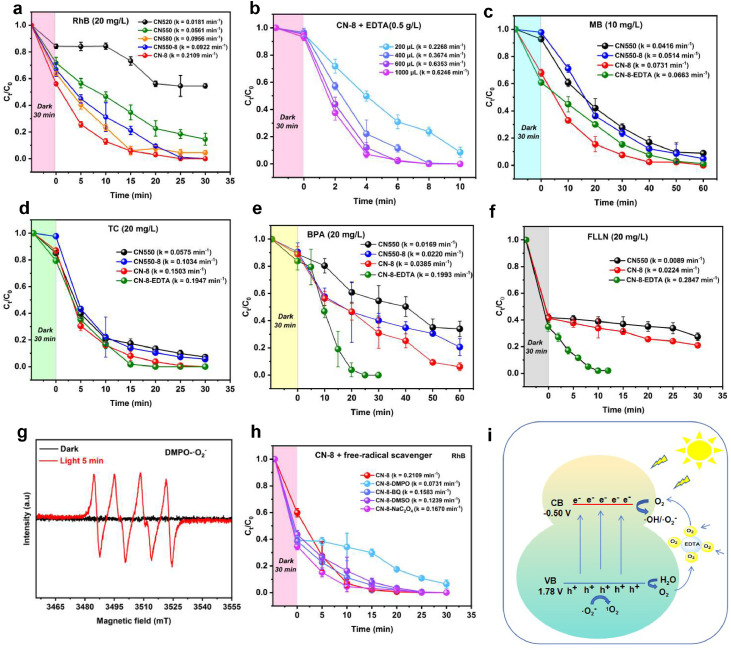
Degradation of RhB (a-b) under the irradiation of a 6 W LED lamp at λ = 450 nm; Degradation of MB (c), TC(d), BPA (e) and FLLN (f) under the irradiation of a 12 W LED lamp at λ = 450 nm; (g) EPR of **·**O^2-^ under dark and light conditions; (h) Degradation of RhB under the Different free radical trapping agents irradiation of a 6 W LED lamp at λ = 450 nm.(i) Illustration of the charge generation, separation and transformation processes in the O_2_/**·**O^2-^/EDTA/CN-8/visible-light system.

We carried out photocatalytic degradation tests of CN-8-EDTA on RhB (Fig. 6**a-c**), MB (Fig. 6**d**), TC (Fig. 6**e**), BPA (Fig. 6**f**) and FLLN (Fig. 6**g**)with a catalyst concentration of 0.5 g/L. The optimal amount of EDTA was determined by screening the degradation rate of RhB, and it was found that adding more than 600 μL did not significantly improve the degradation rate. Hence, we chose to use 600 μL of EDTA. Before the addition of EDTA, the first-order rate constants (*k*) of CN-8 for RhB, MB, TC, BPA and FLLN were 0.2109 min^-1^, 0.0731 min^-1^, 0.1503 min^-1^, 0.0385 min^-1^ and 0.0224 min^-1^ respectively. After adding 600 μL of EDTA, the degradation rates of RhB, TC, BPA and FLLN increased significantly, with k values of 0.6353 min^-1^, 0.1947 min^-1^, 0.1993 min^-1^, and 0.2847 min^-1^ respectively. However, the degradation rate of MB decreased, with a k value of 0.0663 min^-1^. The addition of EDTA facilitated O_2_ reduction through H-bonding, promoting the generation of ·O_2_^−^ and ^1^O_2_, thereby increasing the degradation efficiency. However, in the case of MB, the reaction rate actually decreased, indicating that ·OH is the main active radical for its degradation. To further analyze the degradation of TC, BPA and FLLN, we plotted in situ trace UV-visible absorption spectra (**Figure S9–18**, Supporting Information). We conducted a comparative study with similar catalysts and found that the photocatalytic performance of CN-8 is at the cutting-edge level (**Table S6-S9**, Supporting Information).

In addition, the degradation of RhB remain nearly identical after five cycles, implying our CN-8 catalyst is highly stable and recyclable (**Figure S23**). After 5 cycles, the comparison of XRD (**Figure S24**), FT-IR (**Figure S25**), and SEM (**Figure S26**) between the original CN-8 and after 5 cycles CN-8 shows that the integrity of the corresponding structure of CN-8 remains above 95%. This indicates that the crystal structure and chemical composition of CN-8 are relatively stable. We have further extended the reaction scope to other organic pollutants including MB, TC, BPA and FLLN.

### 2.4 Degradation mechanism

A reasonable reaction mechanism has been proposed in Fig. 6i, considering the aforementioned studies. In neutral conditions (pH 7), electrons and holes are generated on the CN-8 surface through efficient absorption of visible light, leading to charge separation. Oxygen molecules are activated by nearby electrons, resulting in the generation of a large quantity of ·O2− anion radicals through the one-electron reduction of oxygen (E(O_2_/·O_2_^−^) = − 0.33 V vs. NHE, as represented by **Text S1** (Equation (1), Supporting Information).

This process is feasible because the CB edge potential of CN-8 (− 0.50 V vs. NHE) is more negative than the redox potential. Since the VB edge potential of CN-8 (+1.78 V vs. NHE) is less positive than the redox potential of H_2_O/·OH (+ 2.40 V vs. NHE, **Text S1** Equation (2), Supporting Information), it is difficult to generate ·OH radicals on CN-8 through the direct oxidation of ·OH or H_2_O by h^+^. The ·O_2_^−^ anion radicals can abstract another e^−^ to produce the intermediate, H_2_O_2_ (the redox potential of ·O_2_^−^/H_2_O_2_ is 0.94 V vs NHE, **Text S1** Equation (3), Supporting Information). Subsequent one-electron reduction of H_2_O_2_ leads to the generation of ·OH radicals indirectly (the redox potential of H_2_O_2_/·OH is 0.32 V vs NHE, **Text S1** Equation (4), Supporting Information). From the comparison in band structure alignment between CN-8 and CN550, we conclude that the separation of electrons and holes caused by the modification of the amino site is essential for such direct and indirect generation of reactive species (**Text S1** Equation (1–9), Supporting Information). After LC-MS analysis, the possible degradation pathway of FLLN is illustrated in **Figure S19** (**FLLN LC-MS analysis**, Supporting Information). We conducted bio-toxicity tests before and after FLLN degradation, and the results showed a reduction in toxicity by over 95% after degradation (**Figure S20**). The mineralization rates of organic pollutants RhB, MB, TC, BPA, and FLLN, under 12 W LED λ = 450 nm illumination for 60 mins, were found to be 85.07%, 34.68%, 90.95%, 89.13%, and 46.06%, respectively. These results highlight the remarkable efficacy of our approach in degrading these small molecular pollutants (**Figure S21**).

Contribution of h^+^, ·O_2_^−^, ·OH, and ^1^O_2_ to RhB degradation. The decreased degradation kinetics by CN-8 after adding Na_2_C_2_O4, DMPO, BQ, and DMSO into the reaction system suggested that h^+^, ·O_2_^−^, ·OH, and ^1^O_2_ contributed to RhB degradation. where α and β are the decreased degradation kinetic efficiencies and relative contribution of different reactive species to RhB degradation, respectively. *k*_0_ is the pseudo-first-order constant for RhB degradation without a scavenger as the control, *k*(Na_2_C_2_O_4_), *k*(BQ), *k*(DMPO), and *k*(DMSO) are the pseudo-first-order constants for RhB degradation when Na_2_C_2_O_4_, BQ, DMPO, and DMSO were added into the suspension, respectively (**Text S2** Equation (10–17), Supporting Information).

Fundamentally, photochemical oxidation may involve different reactive species like hole (h^+^), superoxide radical (·O_2_^−^), hydroxyl radical (·OH), and singlet oxygen (^1^O_2_). **Figure S22a** shows the ESR spectra using 2,2,6,6-Tetramethylpiperidine 1-oxyl (TEMPOL) as the spin-labeling agent. After 5 mins of illumination, the ESR intensity of TEMPOL did not change, indicating that the photoinduced electrons generated by CN-8 under light exposure were not captured by TEMPOL. Fig. 6**g** and **Figure S22b** shows that spin trapping experiments using 5,5-dimethyl-1-pyrroline N-oxide (DMPO) provide direct evidence of the formation of DMPO-·O_2_^−^ adducts (quartet, 1:1:1:1) and DMPO-·OH adducts (quartet, 1:2:2:1). No EPR signal is observed when there is no DMPO or in the dark. The influence of such reactive species was verified by the use of corresponding scavengers under other identical conditions, namely, sodium oxalate (NaC_2_O_4_, h+ scavenger), benzoquinone (BQ, ·O_2_^−^ scavenger), dimethylsulfoxide (DMSO, ^1^O_2_ scavenger), and dimethylpyridine nitrogen oxide (DMPO, ·OH and ·O_2_^−^ scavenger) were added to the reaction system in separate runs. **Text S2** Equation (13–16) show that β(h^+^) = 16.36 %, β(**·**O_2_^−^) = 19.59 %, β(**·**OH) = 31.67 %, and β(^1^O_2_) = 32.38 % are the decreased degradation kinetic efficiencies and relative contribution of different reactive species to RhB degradation, respectively (Fig. 6**h**). Therefore, based on DFT calculations and experimental characterizations, a feasible mechanism for photocatalytic degradation by CN-8 is proposed (Fig. 6**i**).

## 3 Conclusion

In conclusion, we investigated the changes in the photocatalytic performance of modified g-C_3_N_4_ from a new perspective, taking advantage of benzaldehyde compounds with different electron-donating and electron-withdrawing groups on the amino group. Through DFT calculations and experimental verification, we found that CN-8 exhibited the highest degradation efficiency for MB, reaching 0.0844 min^-^¹. After adding EDTA, the degradation rates of RhB, TC, BPA, and FLLN were significantly increased to 0.6353 min^-1^, 0.1947 min^-1^, 0.1993 min^-1^, and 0.2847 min^-1^, respectively. Mechanistic studies confirmed that introducing strong electron-donating functional groups at the amino position of g-C_3_N_4_ improved the separation of photo-generated carriers. This improvement enabled CN-8 to more effectively generate superoxide radicals under light conditions in the presence of EDTA, thereby promoting the degradation reaction. This study provides a new approach for designing efficient photocatalysts to purify water pollutants.

## 4 Materials and methods

### 4.1 Materials and reagents

Urea, absolute ethyl alcohol, glacial acetic acid, Na_2_C_2_O_4_, DMSO, DMPO, BQ, TEMPOL, RhB, MB, TC, and BPA were purchased from Macklin. 4-fluorobenzaldehyde (CN-1), 2,3-dimethylbenzaldehyde (CN-2), 3-nitrobenzaldehyde (CN-3), 4-bromobenzaldehyde (CN-4), terephthalaldehyde (CN-5), 2-nitrobenzaldehyde (CN-6), 2-bromo-6-nitrobenzaldehyde (CN-7), 4-nitrobenzaldehyde (CN-8), 2,4-dinitrobenzaldehyde (CN-9), 3-fluoro-4-nitrobenzaldehyde (CN-10), 3-methyl-4-nitrobenzaldehyde (CN-11), and 2,6-difluorobenzaldehyde (CN-12) were purchased from Aladdin.

### 4.2 Synthesis of pristine g-C_3_N_4_(CN550) and CN580

The CN550 were fabricated by a typical synthesis route. In brief, urea (10.0 g) in a silica boat was heated at 550 °C for 3 h using a heating rate of 10 °C min^−1^in a muffle furnace in an air atmosphere. The resulting yellow product was pulverized using an agate mortar for further utilization. The synthesis of CN580 is similar to CN550, with the only difference being its calcination temperature at 580 °C.

### 4.3 Synthesis of CN-1∼CN-12

Place 0.5 g of CN580 into a 100 mL round bottom flask and add 50 mL of anhydrous ethanol. Stir the mixture evenly and then add 0.1g of 4-nitrobenzaldehyde (CN-8). Reflux the solution at 80 °C for 12 hours. Once the reaction is complete, pour the mixture into 200 mL of ice water and stir for 10 mins. Then, filter it, rinse it three times with distilled water, followed by three rinses with anhydrous ethanol.^[31]^ Dry the product in an 80 °C oven for 2 hours and grind it into powder using an agate mortar. CN-1 to CN-12 are all prepared using this method with different benzaldehyde compounds.

### 4.4 Material characterizations

The morphology and structure of the samples were characterized by SEM(FEI Nova NanoSEM450), TEM (FEI Talos F200C), and HRTEM. X-ray diffraction (XRD) (Japan Rikagu Ultima IV). X-ray photoelectron spectroscopy was performed using a Thermo SCIENTIFIC ESCALAB 250Xi system . All binding energies were calibrated by using the contaminant carbon (C 1 s = 284.8 eV) as a reference. Fourier transform infrared (FTIR) spectra were obtained on Thermo Fisher iS5. The Bruker EMXPlus used for ESR testing. The full pore was tested using the Micromeritics ASAP2460 from the United States. Diffuse reflectance absorption testing was conducted using the UH4150 instrument. Photocurrent correlation testing was performed using the CHI660e instrument from China-Shanghai Chenhua. PL testing was conducted using the Hitachi F-4700. The electronic transient lifetime was measured using the Horiba Fluorolog-QM steady-state and transient fluorescence phosphorescence spectrometer. The Raman spectroscopy tests using the Thermo DXR2xi instrument from the renowned American company, Thermo Fisher Scientific. The LC-MS tested by the U3000 system from the renowned American company, Thermo Fisher Scientific.The TOC (Total Organic Carbon) testing using the varioTOC select instrument from the renowned German company, Elementar.

### 4.5 Computational methods

All DFT calculations were performed using Gaussian 09 software. The method chosen for structure optimization was DFT-(B3LYP) with a basis set of 6–31G. The single-point energy calculations were performed using TD-DFT-(CAM-B3LYP) with a basis set of 6–31G' d. The data obtained from DFT calculations were processed using Multiwfn software.

### 4.6 Photocatalytic degradation experiment

The photocatalytic degradation experiments were conducted using the PL-SX100A multi-channel photocatalytic reaction instrument. Visible light with a wavelength of λ= 450 nm was selected, and the volume of the degradation solution was 10 mL. The catalyst dosage was 0.5 g/L, and the experiments were conducted in parallel in 3 groups, with the average values taken. For testing RhB, the LED light intensity selected was 6 W (The light intensity is 86.81 mW/cm^2^, with the liquid's surface area in contact with light measuring 37.70 cm^2^), while for testing MB, TC, and BPA, the LED light intensity selected was 12 W (The light intensity is 173.62 mW/cm^2^, with the liquid's surface area in contact with light measuring 37.70 cm^2^). The absorbance values of RhB and MB were measured using the BioTek uQuant full-wavelength microplate reader. The TC and BPA were measured using the Thermo Fisher NanoDrop One microvolume UV–Vis spectrophotometer.

### 4.7 Regression analysis

We used Origin Pro 2022 software to analyze the measured results. The iterations were conducted until a Chi-square tolerance of 1 × 10^−9^ was reached, and the fits were iterated until at least 95% of the data matched the model (R^2^ > 0.95).

## CRediT authorship contribution statement

**Hong Tu:** Writing – original draft, Visualization, Supervision, Methodology, Investigation, Data curation, Conceptualization. **Bihong Tian:** Investigation. **Zhichao Zhao:** Investigation. **Renjiang Guo:** Investigation. **Ya Wang:** Investigation. **Shunhong Chen:** Investigation. **Jian Wu:** Writing – review & editing, Supervision, Funding acquisition, Conceptualization.

## Declaration of competing interest

The authors declare that they have no known competing financial interests or personal relationships that could have appeared to influence the work reported in this paper.

## Data Availability

Data will be made available on request

## Appendix Supplementary materials

Image, application 1
