# Supplementary material for: Research on the influence of g-C3N4 microstructure changes on the efficiency of visible light photocatalytic degradation
Source: Water Res X. 2025 Feb 8;28:100315. doi: 10.1016/j.wroa.2025.100315 (PMC11869602; doi:10.1016/j.wroa.2025.100315)
Supplement: Supplementary file 1 [file mmc1.docx]

**Supporting Information**

**Research on the influence of g-C_3_N_4_ microstructure changes on the efficiency of visible light photocatalytic degradation**

Hong Tu, Bihong Tian, Zhichao Zhao, Renjiang Guo, Ya Wang, Shunhong Chen, Jian Wu*

*State Key Laboratory of Green Pesticide, Key Laboratory of Green Pesticide and Agricultural Bioengineering, Ministry of Education, Guizhou University, Huaxi District, Guiyang 550025, China*

*Corresponding author. E-mail: [jwu6@gzu.edu.cn](mailto:jwu6@gzu.edu.cn)

**Table of Contents**

**Text S1.** Possible reaction paths (pH = 7) for DFT calculations: (**4P**)

**Text S2.** The degradation kinetic rate constants (k) were further analyzed. The equation for the kinetics is as follows: (**5p**)

**Figure S1.** SEM images of (a) CN520, (b) CN550, (c) CN580, (d) CN-8. (**6P**)

**Figure S2.** SEM images of CN-1~CN-12 at 500 nm. (**7P**)

**Figure S3.** (a-b) SEM and (c–e) TEM images of CN-8; (f-h) the corresponding elemental mapping on C, N, and O. (**8P**)

**Figure S4.** The XPS analysis of CN-8 was performed under an N_2_:O_2_ atmosphere ratio of 8:2, including measurements under dark and 5-minute light conditions. (**9P**)

**Figure S5.** UV-vis DRS spectra of CN520, CN550,CN55-8 and CN-8. (**10P**)

**Figure S6.** Mott-Schottky plots of CN550-8. The potential values in these figures can be converted to potential relative to the standard hydrogen electrode (NHE) using the formula: E (NHE) = E (Ag/AgCl) + 0.197 V. (**11P**)

**Figure S7.** The bond length distance between the modification position and the functional group atoms. (**12P**)

**Figure S8.** Visualization of the First Excited State electron-holes in CN-1~CN-12. (**13P**)

**Figure S9.** In situ monitoring of the degradation of BPA by CN550, CN550-8, and CN-8. (**14P**)

**Figure S10.** In situ monitoring of the degradation of TC by CN550, CN550-8, and CN-8. (**15P**)

**Figure S11.** In situ monitoring of the degradation of FLLN by CN550, CN-8 and CN-8-EDTA. (**16P**)

**Figure S12.** The degradation rate of RhB is iteratively fitted until at least 95% of the data matches the model (R^2^ > 0.95). (**17P**)

**Figure S13.** The degradation rate of CN-8-EDTA is iteratively fitted until at least 95% of the data matches the model (R^2^ > 0.95). (**18P**)

**Figure S14.** The degradation rate of free radical capture of CN-8 is iteratively fitted until at least 95% of the data matches the model (R^2^ > 0.95). (**19P**)

**Figure S15.** The degradation rate of MB is iteratively fitted until at least 95% of the data matches the model (R^2^ > 0.95). (**20P**)

**Figure S16.** The degradation rate of TC is iteratively fitted until at least 95% of the data matches the model (R^2^ > 0.95). (**21P**)

**Figure S17.** The degradation rate of BPA is iteratively fitted until at least 95% of the data matches the model (R^2^ > 0.95). (**22P**)

**Figure S18.** The degradation rate of FLLN is iteratively fitted until at least 95% of the data matches the model (R^2^ > 0.95). (**23P**)

**Figure S19.** Possible degradation pathways of FLLN inferred from LC-MS analysis. (**24P**)

**Figure S20.** Testing of toxicity against the diamondback moth using immersion method: (a) solution of 20 mg/L FLLN, (b) solution after 60 minutes of photocatalytic degradation. (**25P**)

**Figure S21.** Mineralization rate after 60 minutes of 450 nm 12W LED light exposure. (**26P**)

**Figure S22.** EPR of **·**OH and e^-^ under dark and light conditions. (**27P**)

**Figure S23.** Rate of degradation over 5 cycles. (**28P**)

**Figure S24.** XRD comparison between original CN-8 and CN-8 after 5 cycles. (**29P**)

**Figure S25.** FT-IR comparison of CN-8 after 5 cycles. (**30P**)

**Figure S26.** (a-b) Original CN-8 (c-d) CN-8 after 5 cycles. (**31P**)

**Table S1.** Surface compositions of the CN550, CN550-8, CN-8 obtained from the XPS analysis. (**32P**)

**Table S2.** BET surface area, average pore size, and pore volume of CN520, CN550, and CN-8. (**33P**)

**Table S3.** Comparison of the contribution of Fragment 1 to the first excited state electron holes and its degradation rate of RhB. (**34P**)

**Table S4.** DFT calculation of excitation energy. (**35P**)

**Table S5.** Excitation State 1 contribution of each fragment to holes and electrons. (**36P**)

**Table S6.** Comparison of photocatalytic RhB properties of g-C_3_N_4_-based photocatalyst. (**37P**)

**Table S7.** Comparison of photocatalytic MB properties of g-C_3_N_4_-based photocatalyst. (**38P**)

**Table S8.** Comparison of photocatalytic TC properties of g-C_3_N_4_-based photocatalyst. (**39P**)

**Table S9.** Comparison of photocatalytic BPA properties of g-C_3_N_4_-based photocatalyst. (**40P**)

**FLLN LC-MS analysis** (**41-45P**)

**References** (**46-47P**)

**Supplementary** **Texts**

**Text S1.**

Possible reaction paths (pH = 7) for DFT calculations:

Equation (1) $e^{-}$ + $O_{2}$ → **·**$O_{2}^{-}$  $E_{(O_{2}/\boldsymbol{\cdot}O_{2}^{-})}$ = $-$0.33 *V* vs. *NHE*

Equation (2) $H_{2}O$ + $h^{+}$ → **·**$OH$ + $H^{+}$ $E_{(H_{2}O/\cdot OH)}$ = +2.4 *V* vs. *NHE*

Equation (3) $e^{-}$ + **·**$O_{2}^{-}$ + 2$H^{+}$ → $H_{2}O_{2}$ $E_{(\boldsymbol{\cdot}O_{2}^{-}/H_{2}O_{2})}$ = 0.94 *V* vs. *NHE*

Equation (4) $e^{-}$ $+$ $H_{2}O_{2}$ → **·**$OH$ + $H^{+}$  $E_{(H_{2}O_{2}/\boldsymbol{\cdot}OH)}$ = 0.32 *V* vs. *NHE*

Equation (5) $H_{2}O$ + ${2h}^{+}$ → $\frac{1}{2}O_{2}$ + 2$H^{+}$ $E_{(O_{2}/H_{2}O)}$ = +0.82 *V* vs. *NHE*

Equation (6) $O_{2}$ $+$ $hv$ → ^1^$O_{2}$

Equation (7) **·**$O_{2}^{-}$ $+h^{+}$ → ^1^$O_{2}$

Equation (8) $E_{CB}$ = *E_CB_*(pH = 0) $-$ 0.0591pH

Equation (9) $E_{VB}$ = *E_VB_*(pH = 0) $-$ 0.0591pH

**Text S2.**

The degradation kinetic rate constants (k) were further analyzed. The equation for the kinetics is as follows:

$$\text{ln}\text{(}\text{C}\text{t}\text{/}\text{C}\text{0}\text{) = }\text{−}\text{k}\text{ }\text{×}\text{ t}$$

C_0_ and C_t_ refer to the concentration of the organic pollutant at the beginning of the degradation process and at a specific time during the process, respectively.

The relatively contribution of four reactive species was calculated by using following equations:

Equation (10) *α* ($h^{+}$) = $\frac{k_{0} - k_{{Na}_{2}C_{2}O_{4}}}{k_{0}}$ × 100%

Equation (11) *α* (**·**O_2_^−^) = $\frac{k_{0} - k_{BQ}}{k_{0}}$ × 100%

Equation (12) *α* (**·**OH) = $\frac{{{(k}_{0} - k_{DMPO}) - (k}_{0} - k_{BQ})}{k_{0}}$ × 100%

Equation (13) *α* (^1^O_2_) = $\frac{{k_{0}- k}_{DMSO}}{k_{0}}$ × 100%

Equation (14) *β* ($h^{+}$) = $\frac{\alpha(h^{+})}{\alpha(h^{+}) + \alpha(\boldsymbol{\cdot}{O_{2}}^{\mathbf{-}}) + \alpha(\boldsymbol{\cdot}\mathrm{OH}) +\alpha({1_{O}}_{2})}$ × 100%

Equation (15) *β* (**·**O_2_^−^) = $\frac{\alpha(\boldsymbol{\cdot}{O_{2}}^{\mathbf{-}})}{\alpha(h^{+}) + \alpha(\boldsymbol{\cdot}{O_{2}}^{\mathbf{-}}) + \alpha(\boldsymbol{\cdot}\mathrm{OH}) +\alpha({1_{O}}_{2})}$ × 100%

Equation (16) *β* (**·**OH) = $\frac{\alpha(\boldsymbol{\cdot}\mathrm{OH})}{\alpha(h^{+}) + \alpha(\boldsymbol{\cdot}{O_{2}}^{\mathbf{-}}) + \alpha(\boldsymbol{\cdot}\mathrm{OH}) +\alpha({1_{O}}_{2})}$ × 100%

Equation (17) *β* (^1^O_2_) = $\frac{\alpha({1_{O}}_{2})}{\alpha(h^{+}) + \alpha(\boldsymbol{\cdot}{O_{2}}^{\mathbf{-}}) + \alpha(\boldsymbol{\cdot}\mathrm{OH}) +\alpha({1_{O}}_{2})}$ × 100%

**Supplementary Figures**


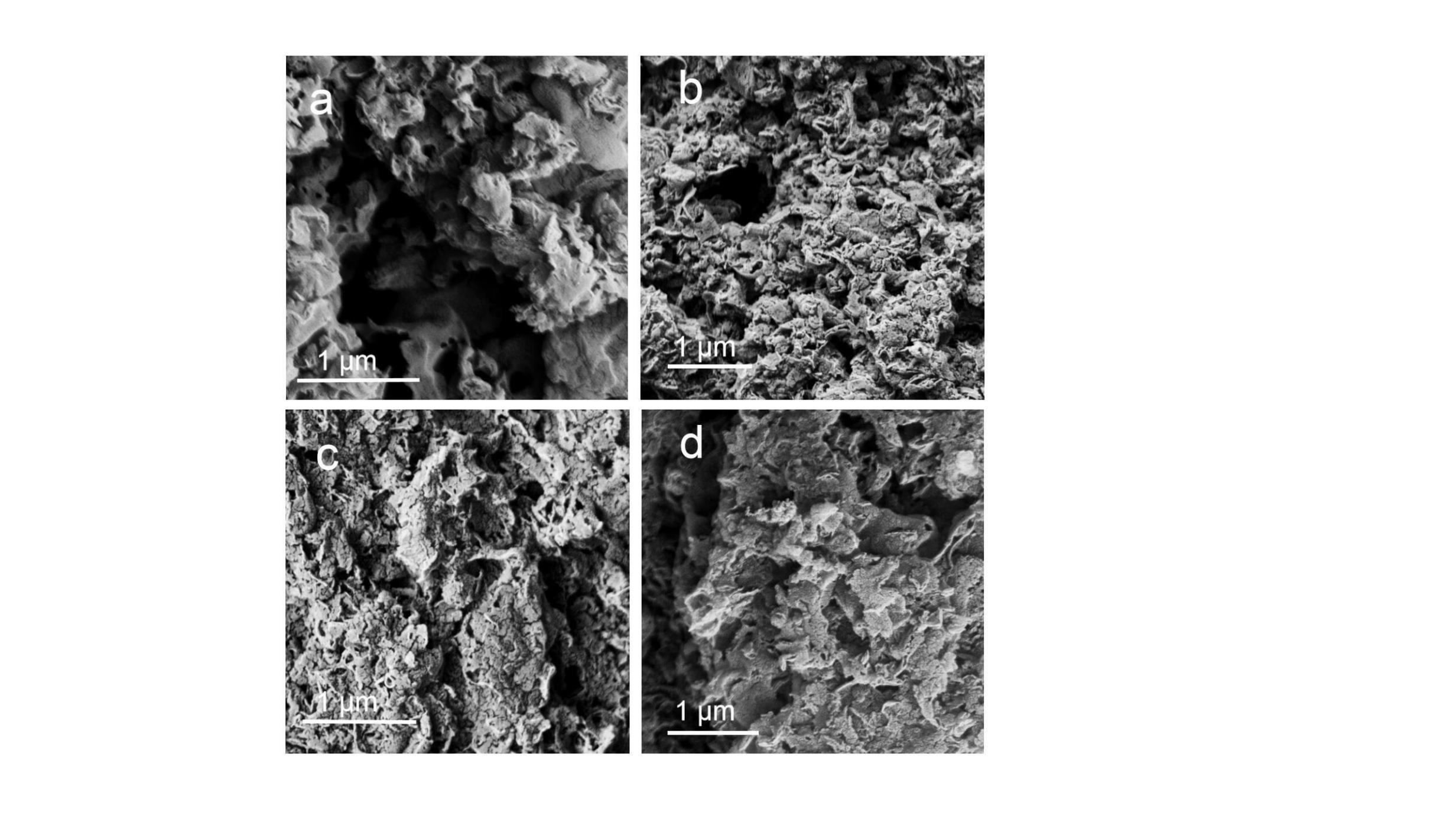


**Figure S1.** SEM images of (a) CN520, (b) CN550, (c) CN580, (d) CN-8.


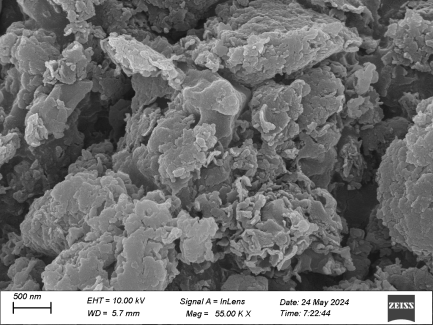


**CN-1**


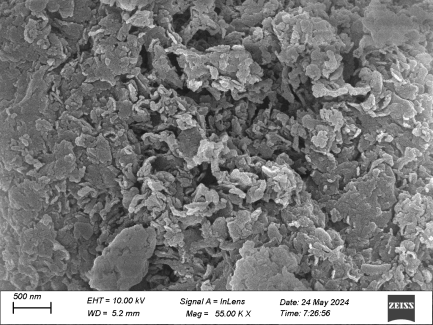

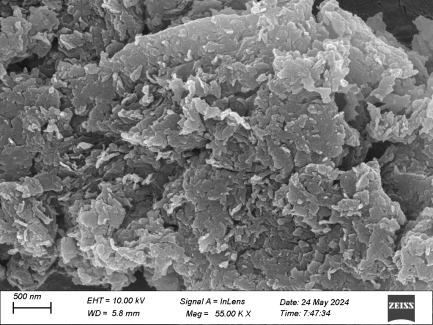


**CN-2**

**CN-3**


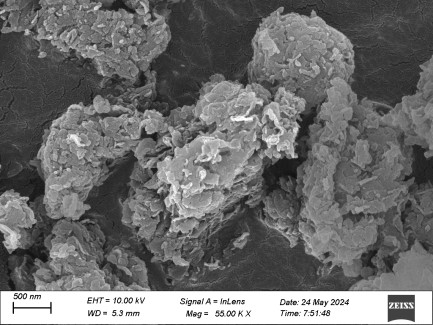

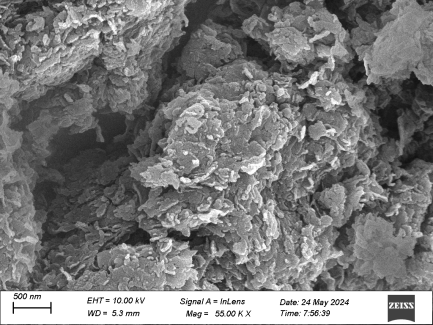

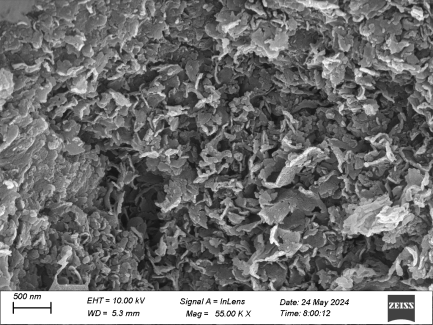


**CN-6**

**CN-5**

**CN-4**


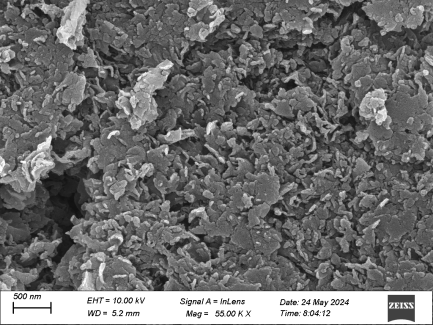

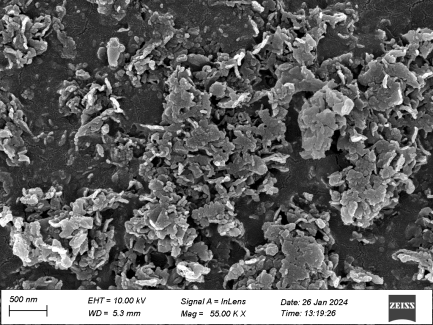

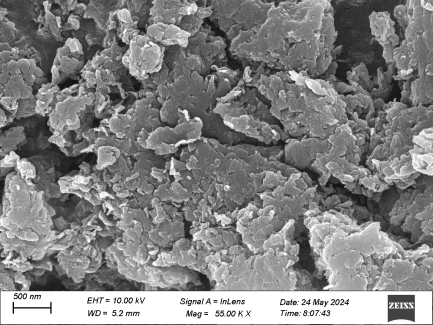


**CN-7**

**CN-8**

**CN-9**


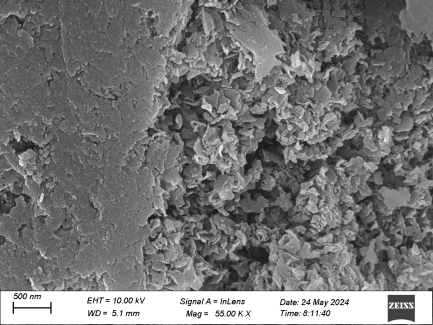

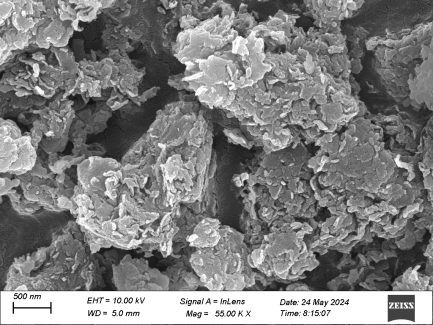

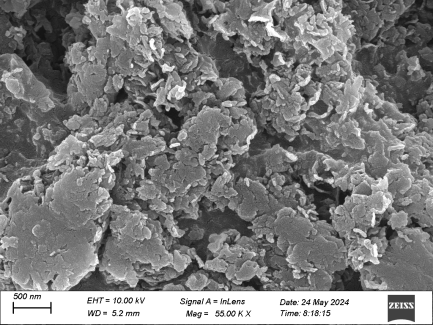


**CN-10**

**CN-11**

**CN-12**

**Figure S2.** SEM images of CN-1~CN-12 at 500 nm.


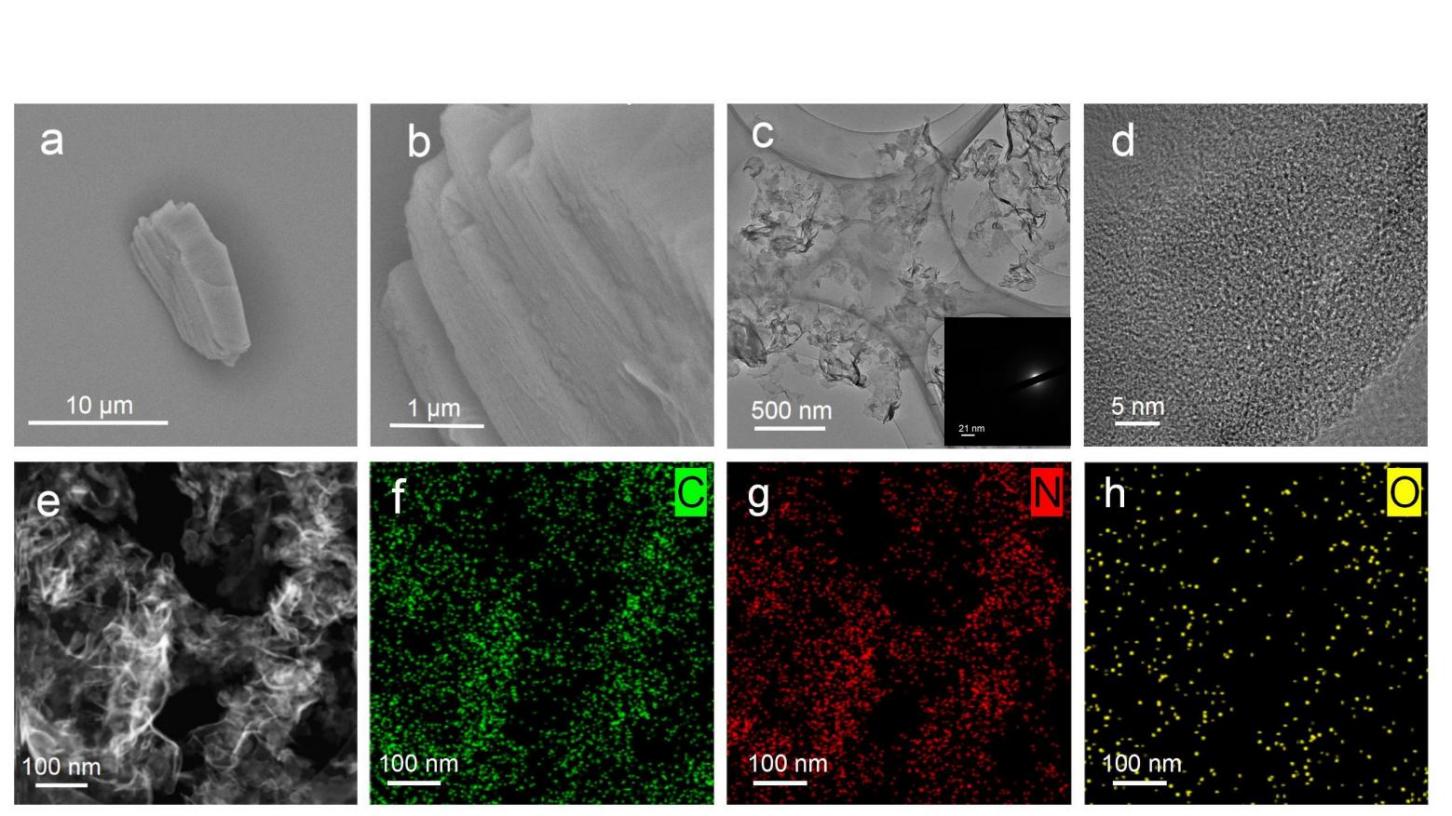


**Figure S3.** (a-b) SEM and (c–e) TEM images of CN-8; (f-h) the corresponding elemental mapping on C, N, and O.


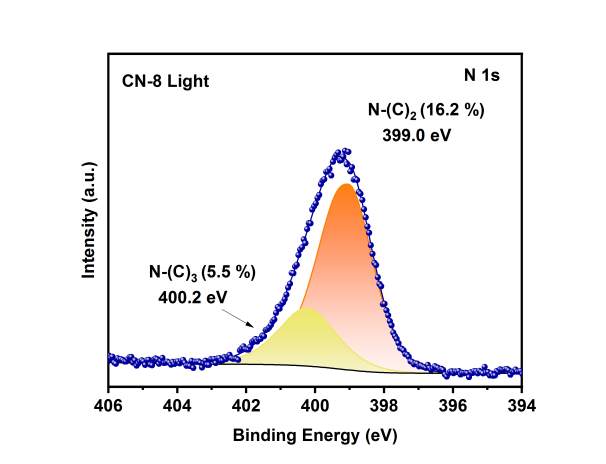

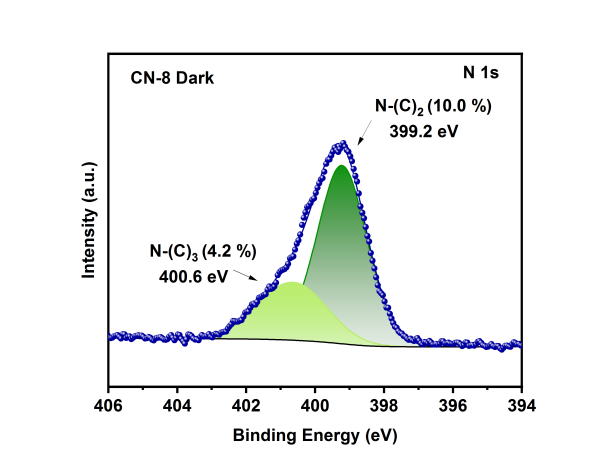

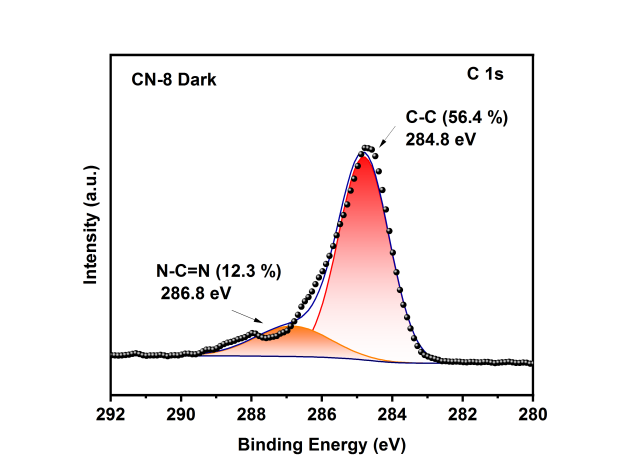

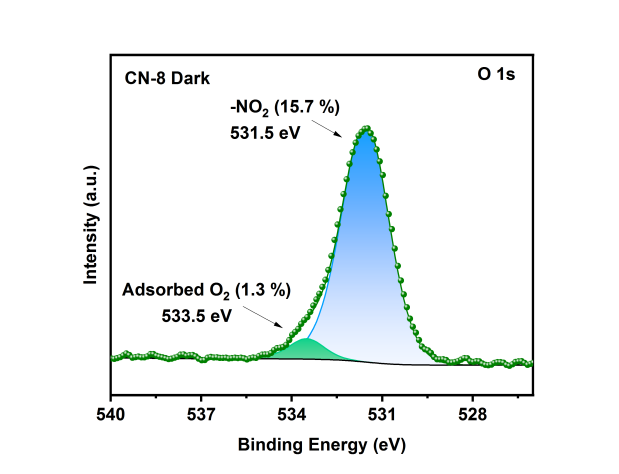

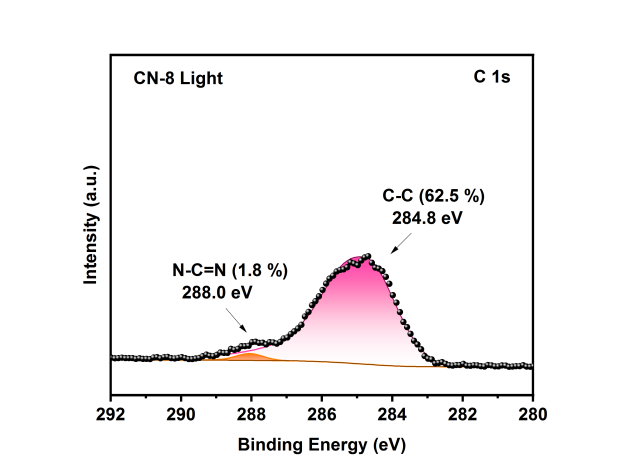

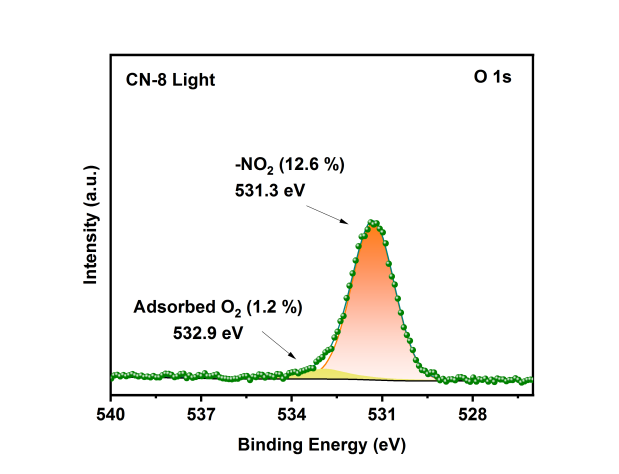

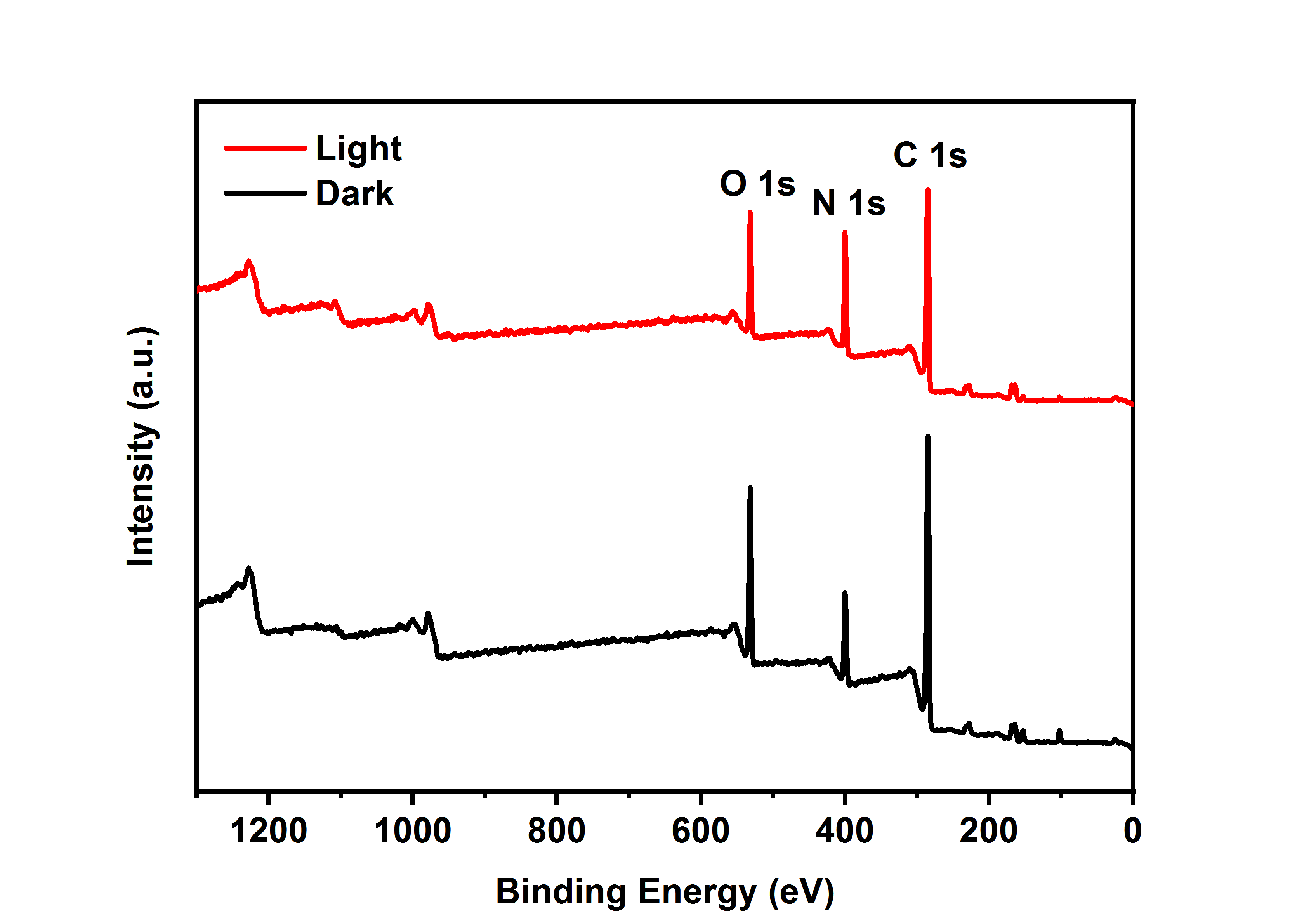

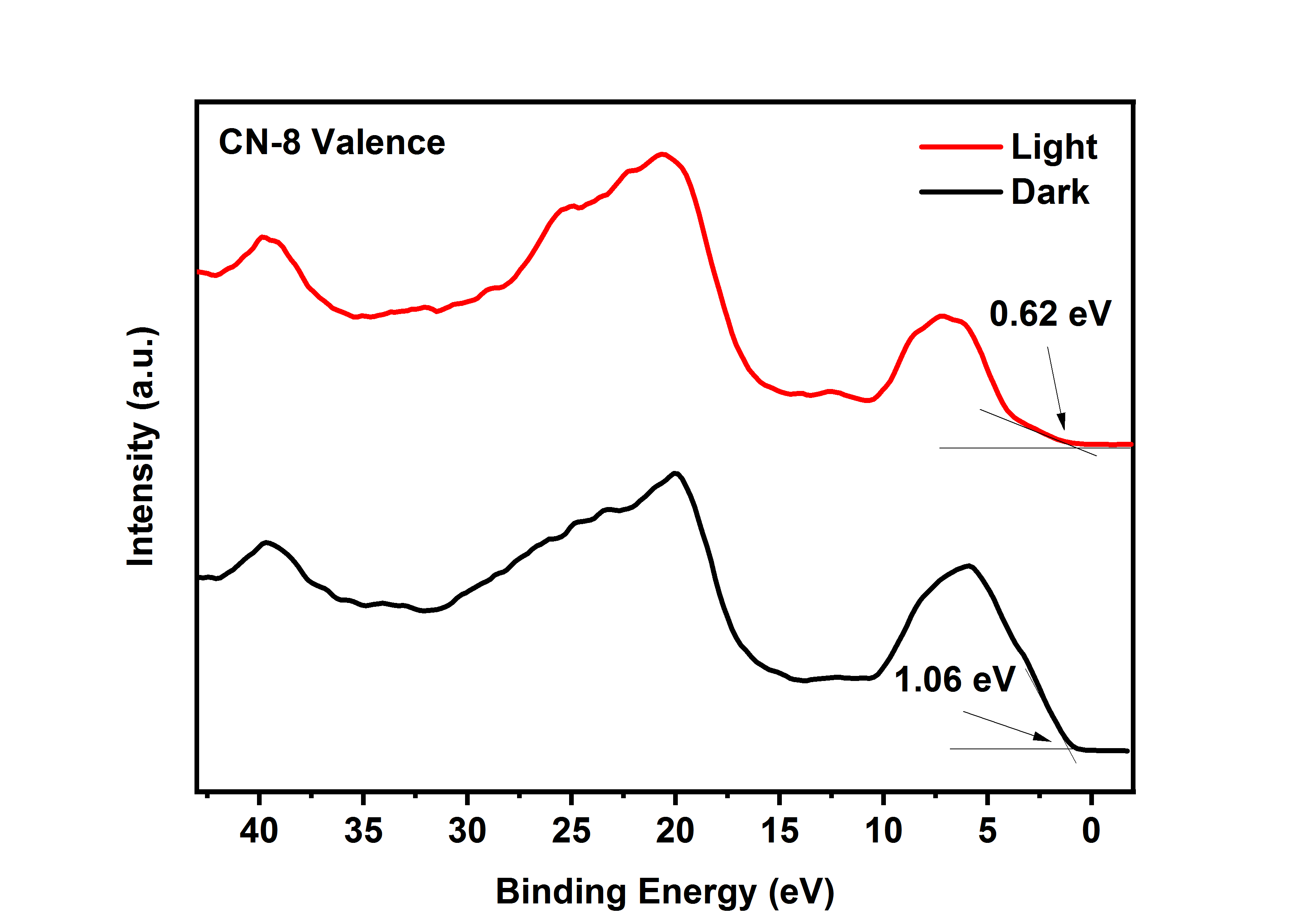


**a**

**b**

**c**

**d**

**e**

**f**

**g**

**h**

**Figure S4.** The XPS analysis of CN-8 was performed under an N_2_:O_2_ atmosphere ratio of 8:2, including measurements under dark and 5-minute light conditions.


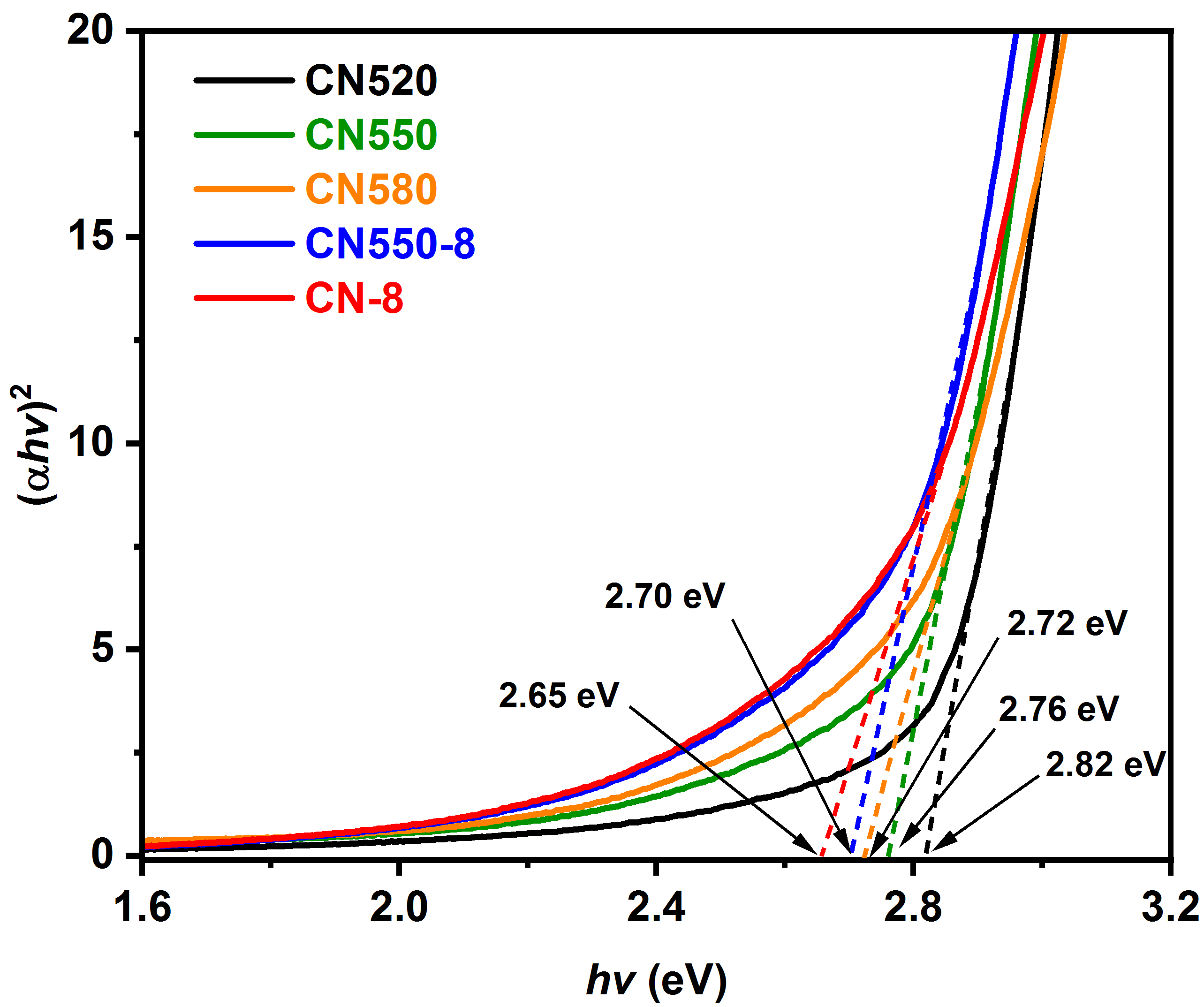

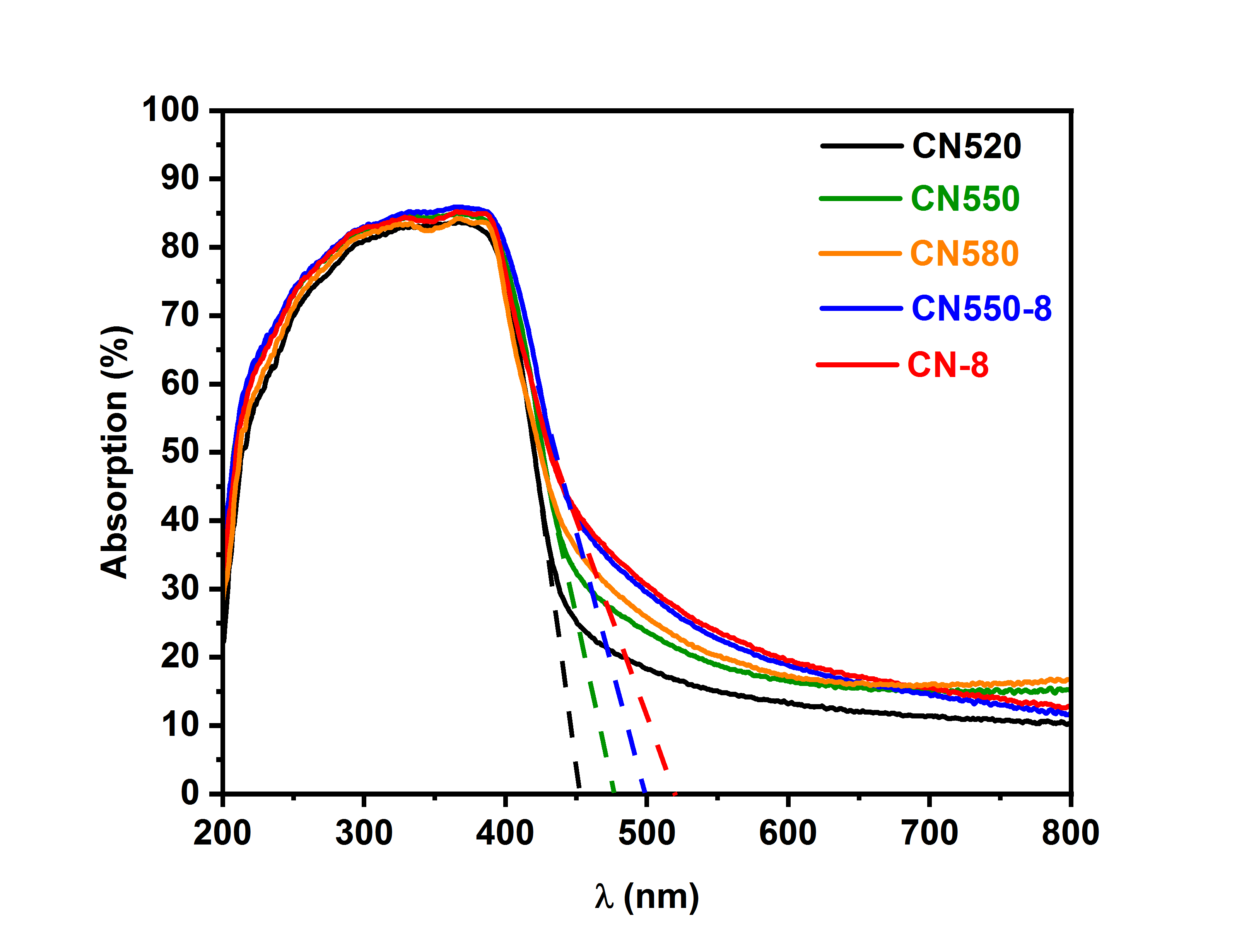


**a**

**b**

**Figure S5.** UV-vis DRS spectra of CN520, CN550,CN55-8 and CN-8.


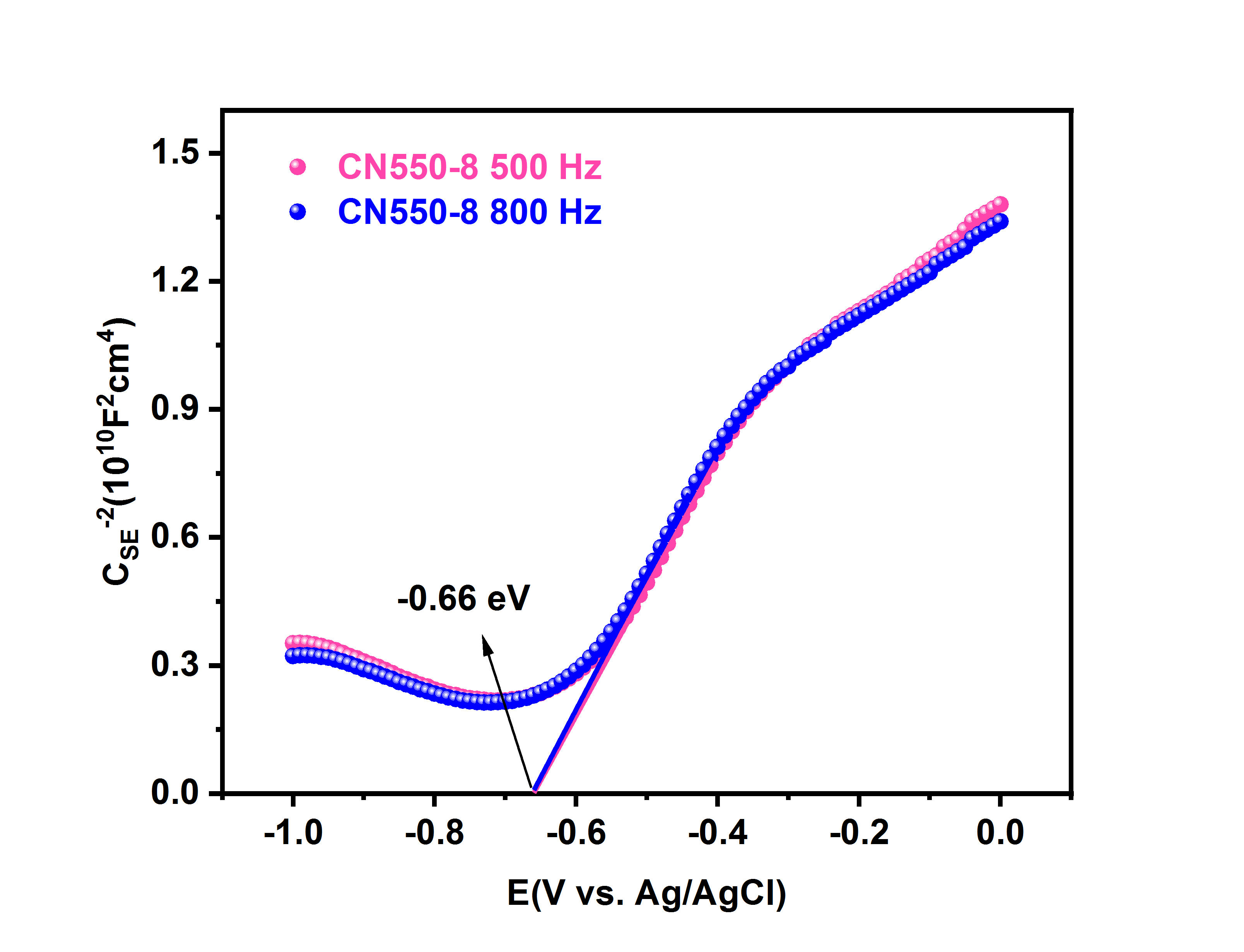


**Figure S6.** Mott-Schottky plots of CN550-8. The potential values in these figures can be converted to potential relative to the standard hydrogen electrode (NHE) using the formula: E (NHE) = E (Ag/AgCl) + 0.197 V.

**
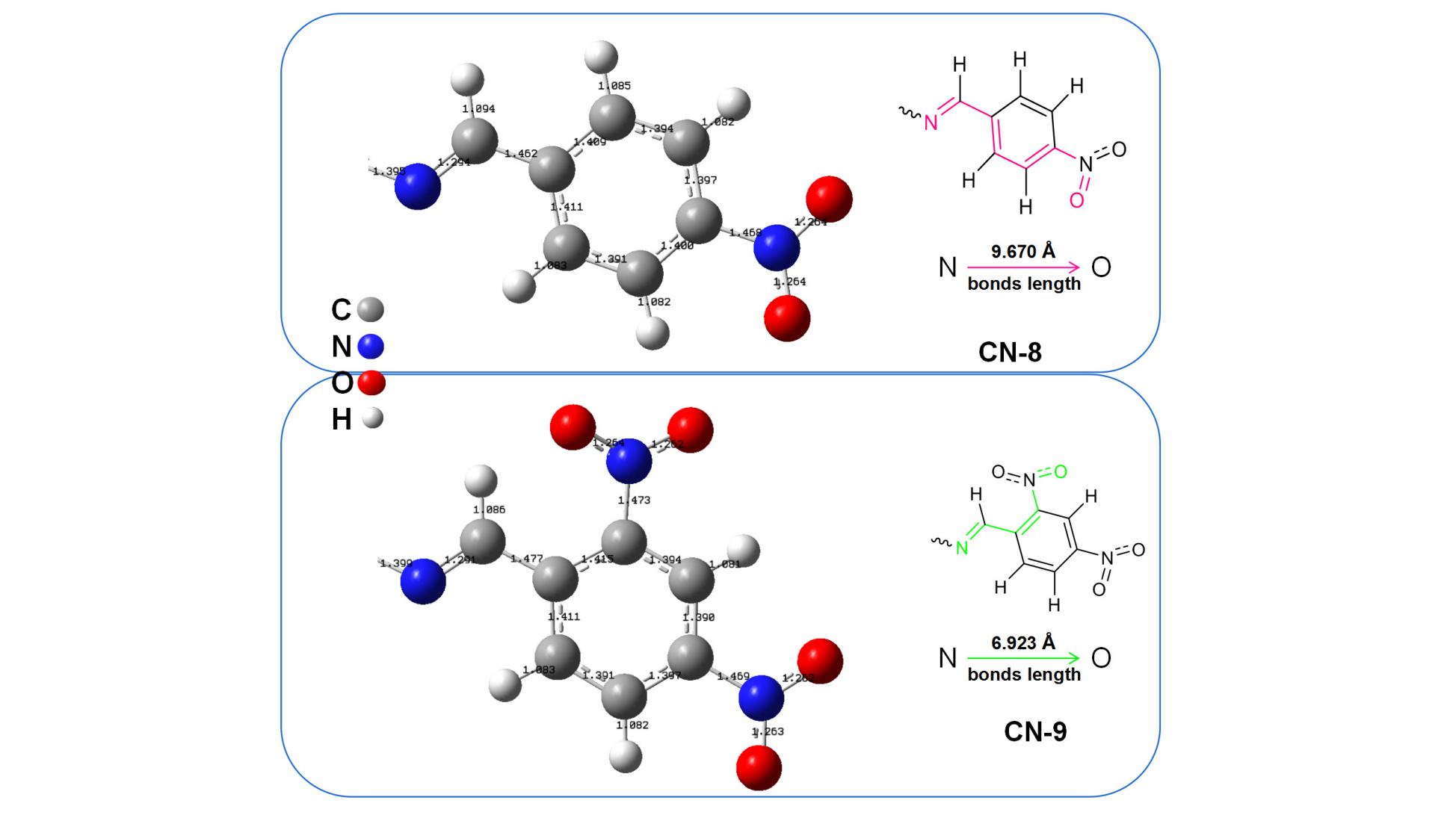
**

**Figure S7.** The bond length distance between the modification position and the functional group atoms.


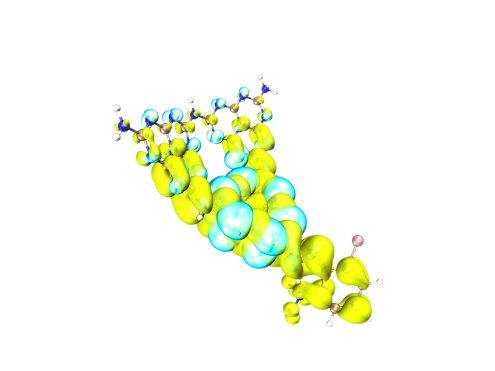

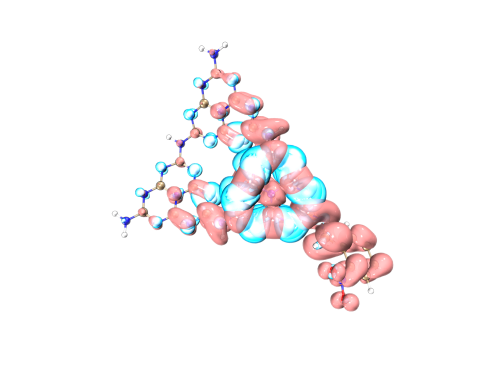

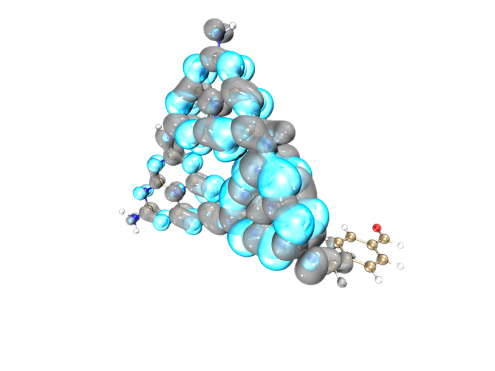

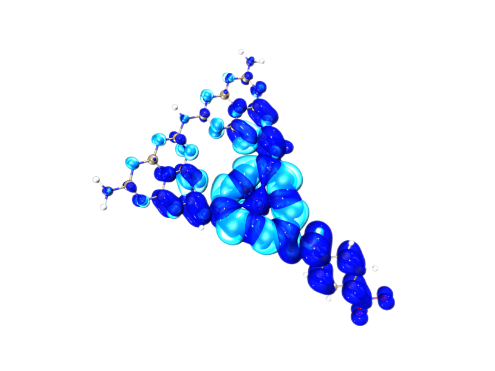

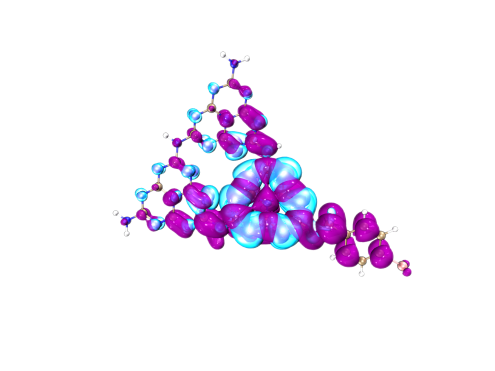

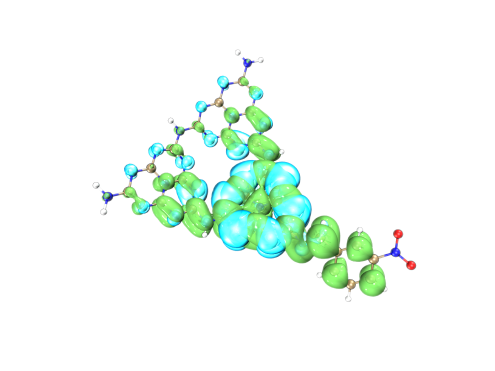

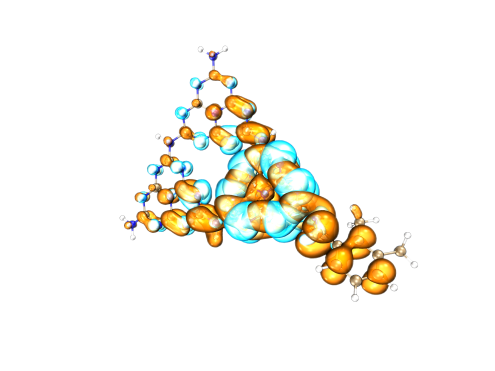

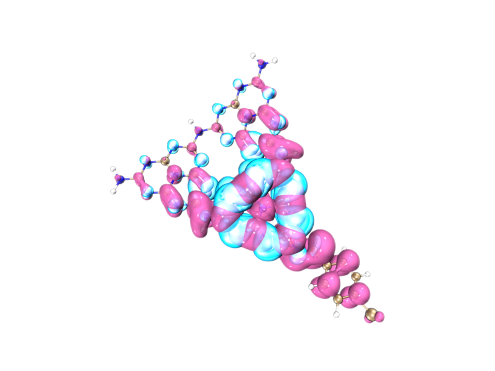

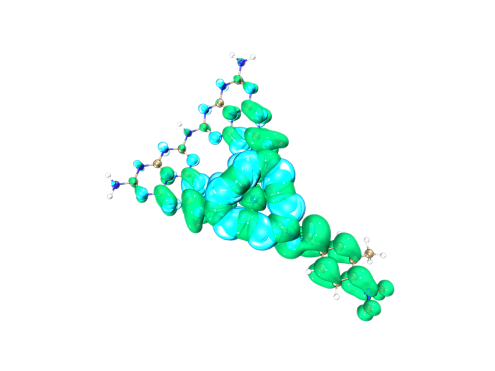


**CN-1**

**CN-2**

**CN-4**

**CN-10**

**CN-3**

**CN-5**

**CN-6**

**CN-8**

**CN-7**

**CN-11**

**CN-9**

**CN-12**


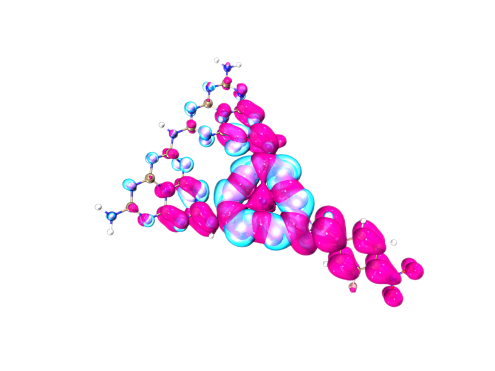

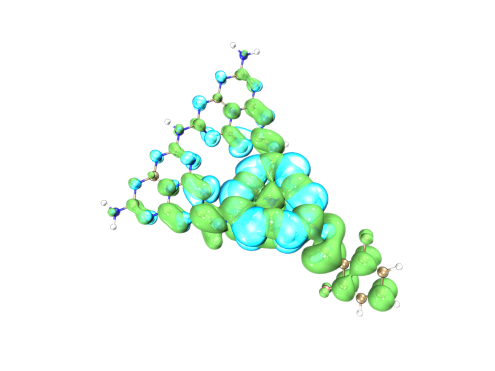

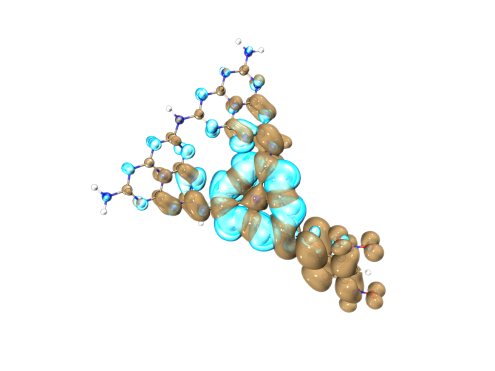

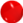

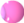

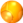

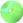

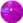

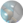

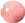

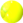

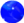

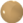

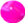

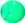

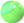

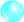


**hole**

**electron**

**Figure S8.** Visualization of the First Excited State electron-holes in CN-1~CN-12.


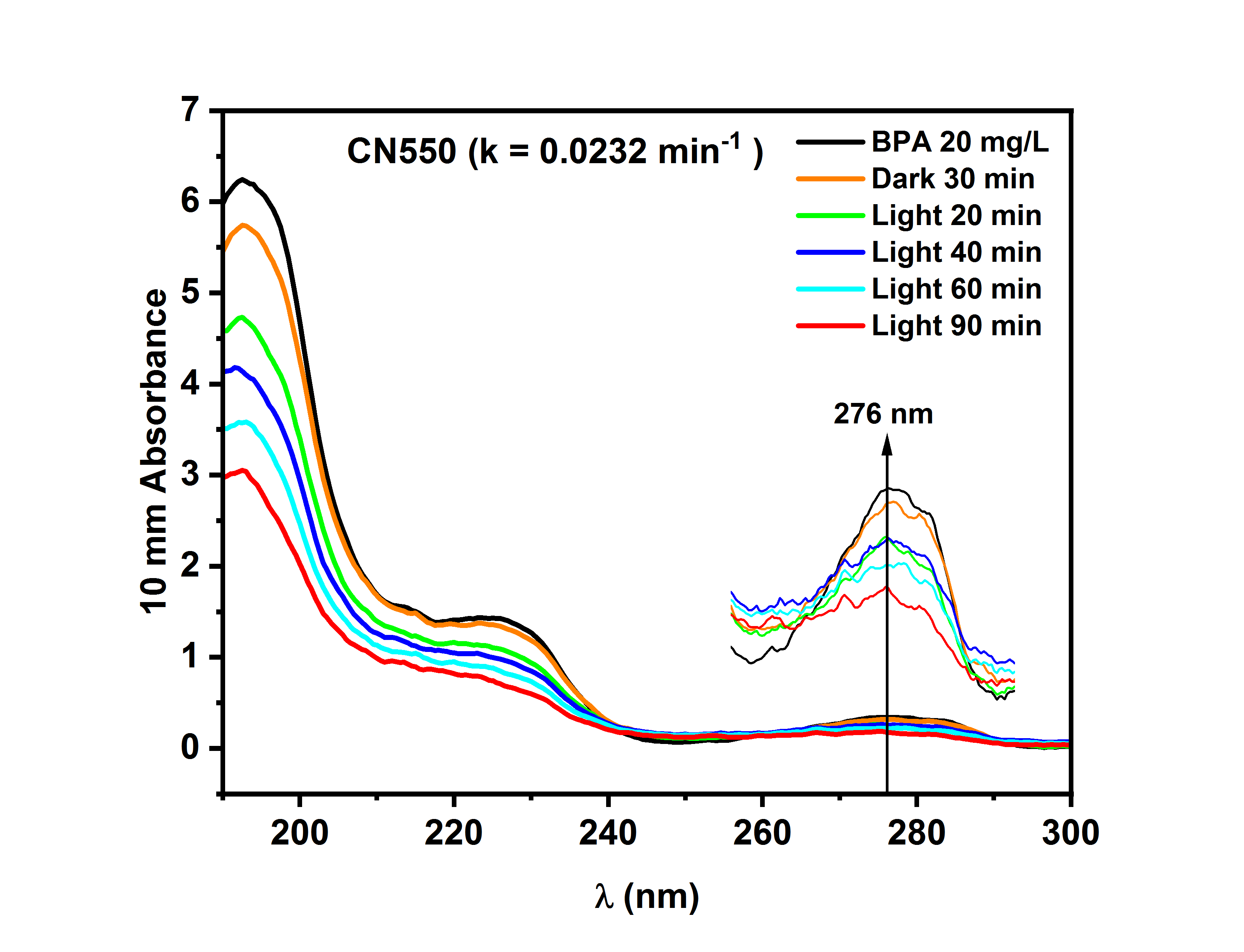

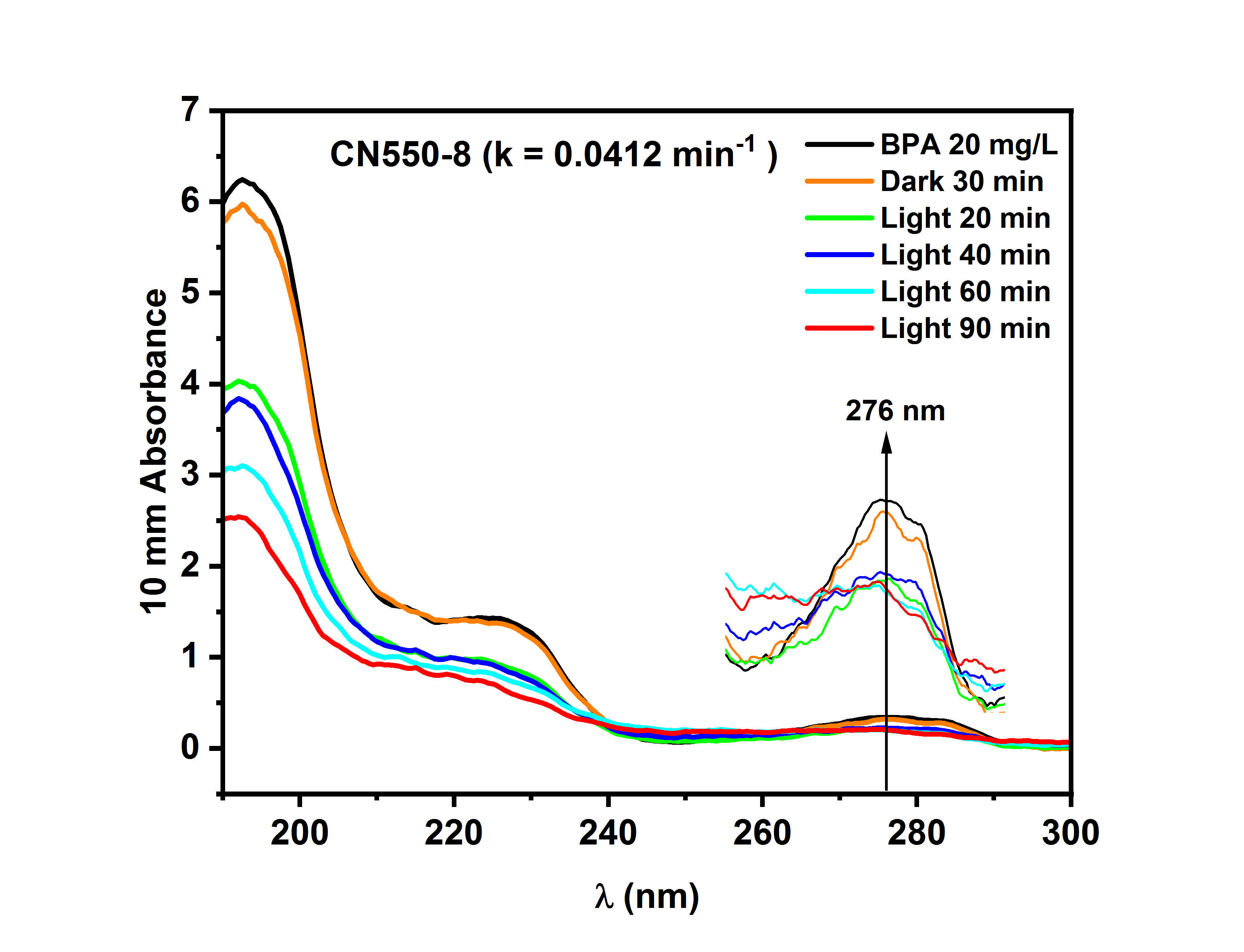

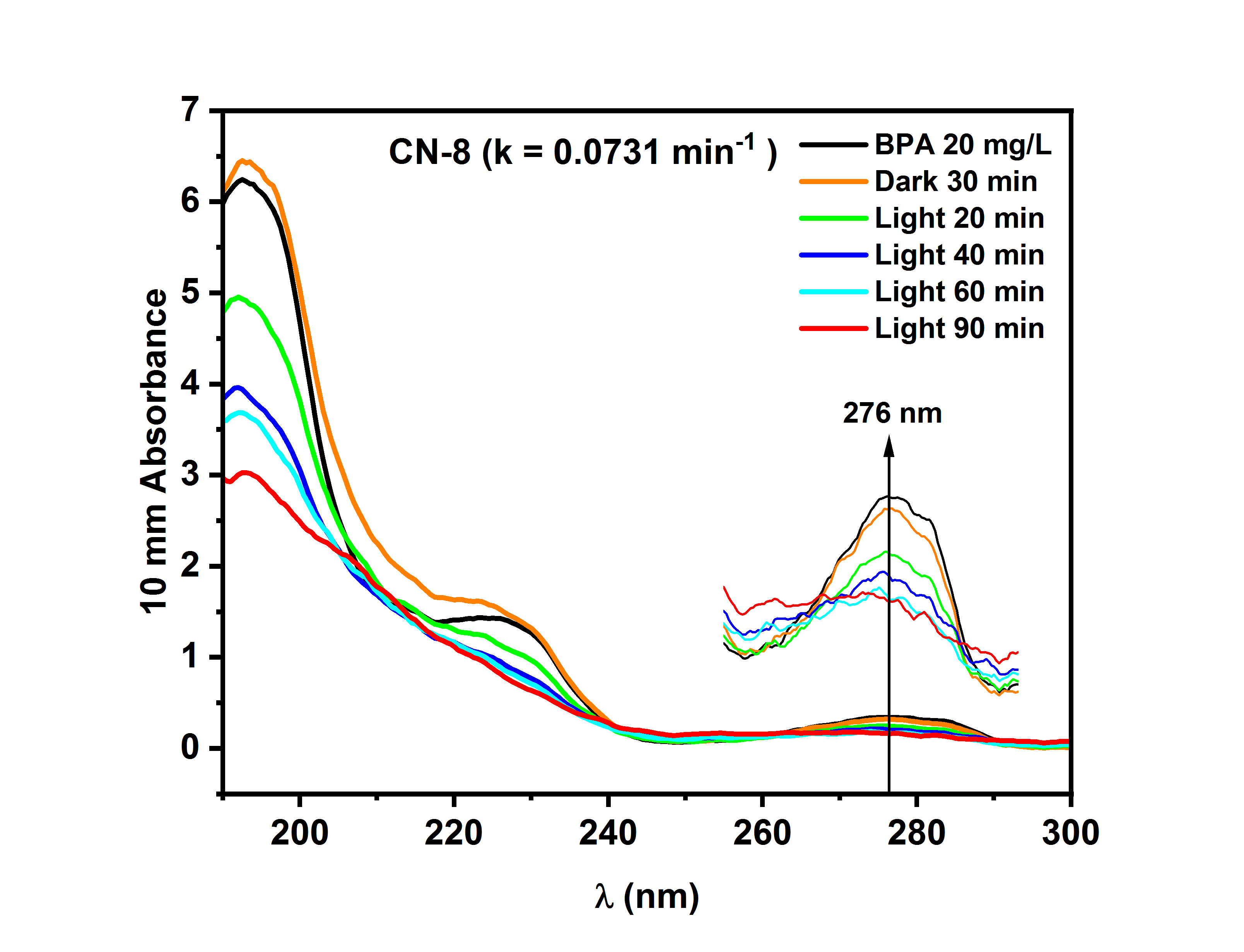


**a**

**b**

**c**

**Figure S9.** In situ monitoring of the degradation of BPA by CN550, CN550-8, and CN-8.


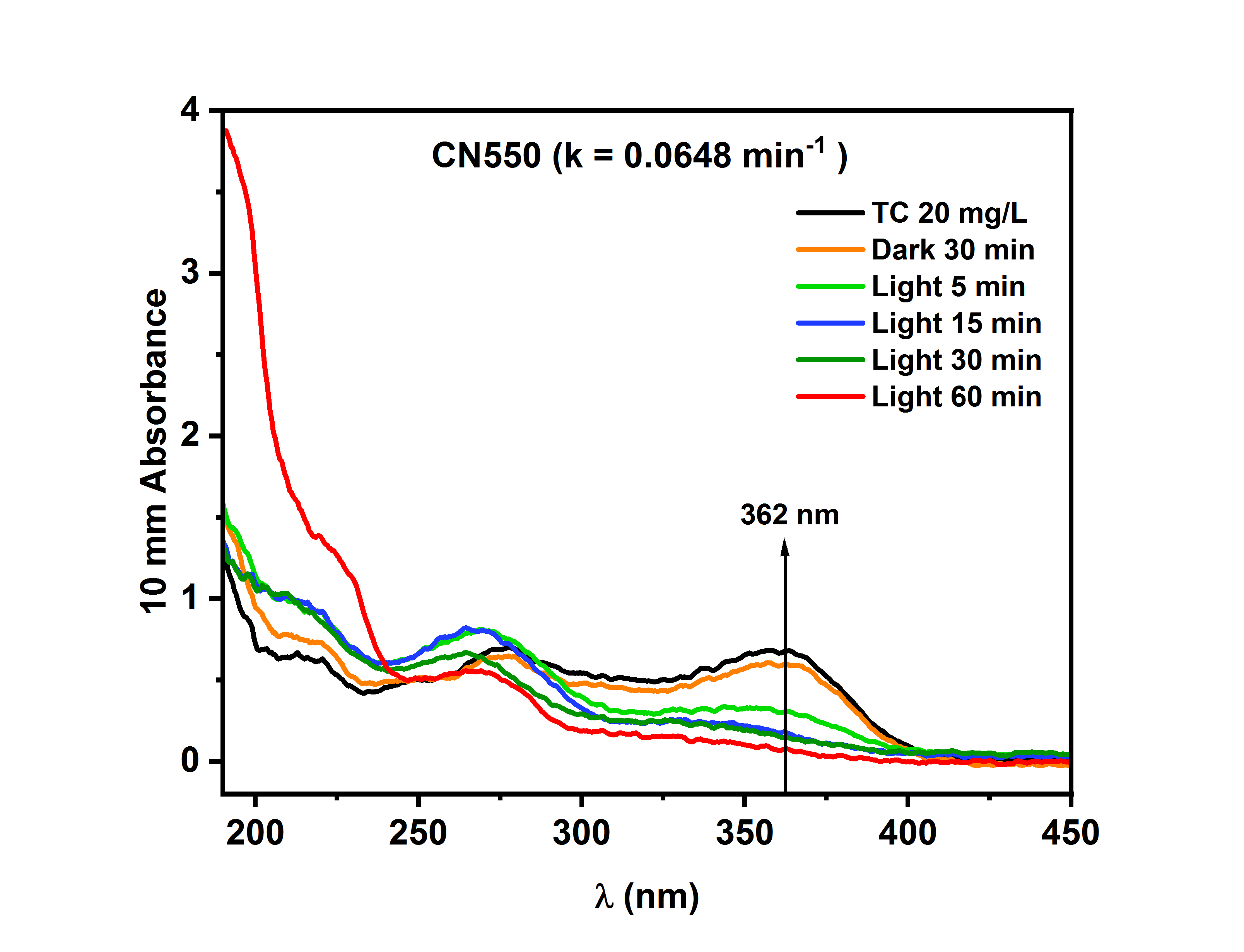

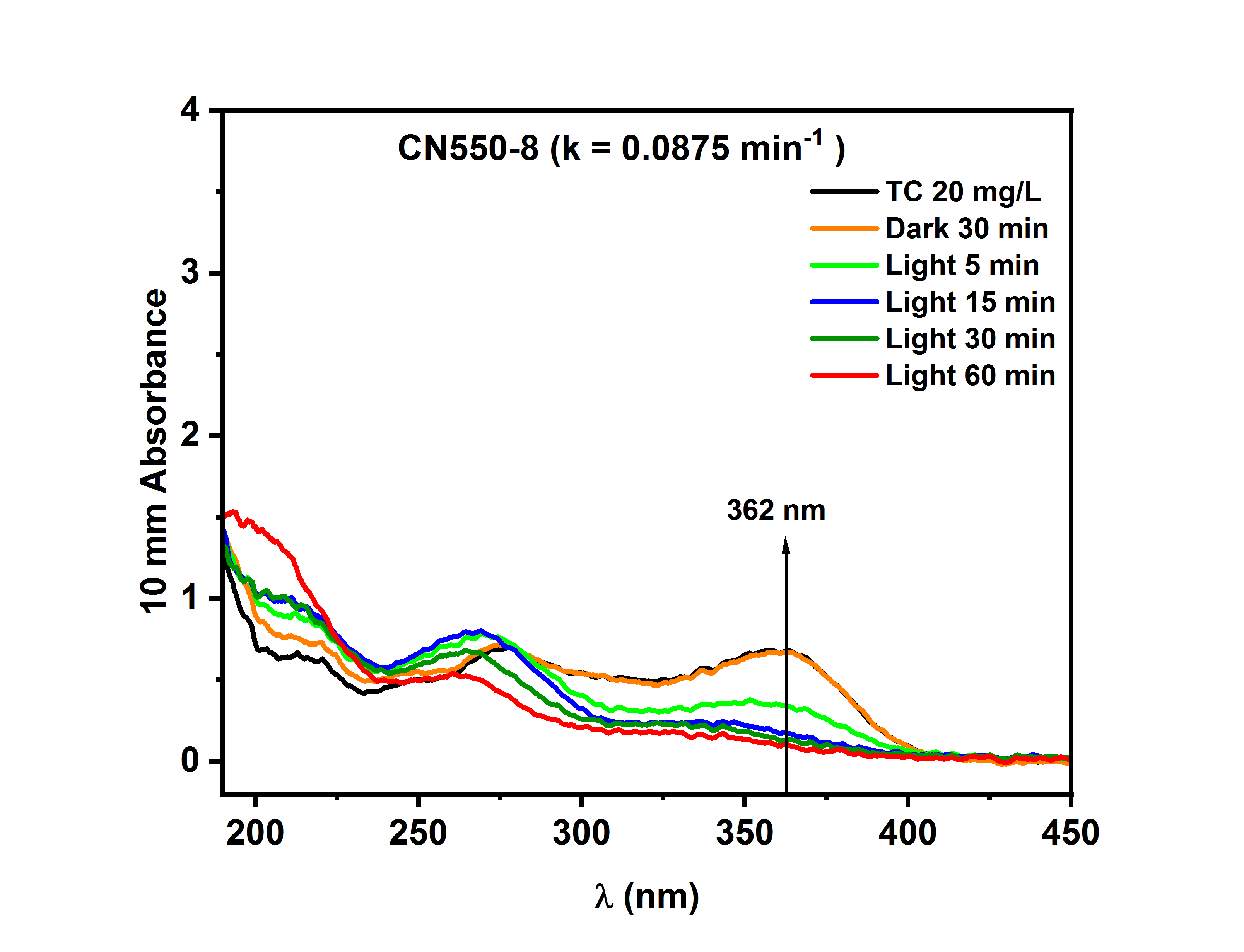

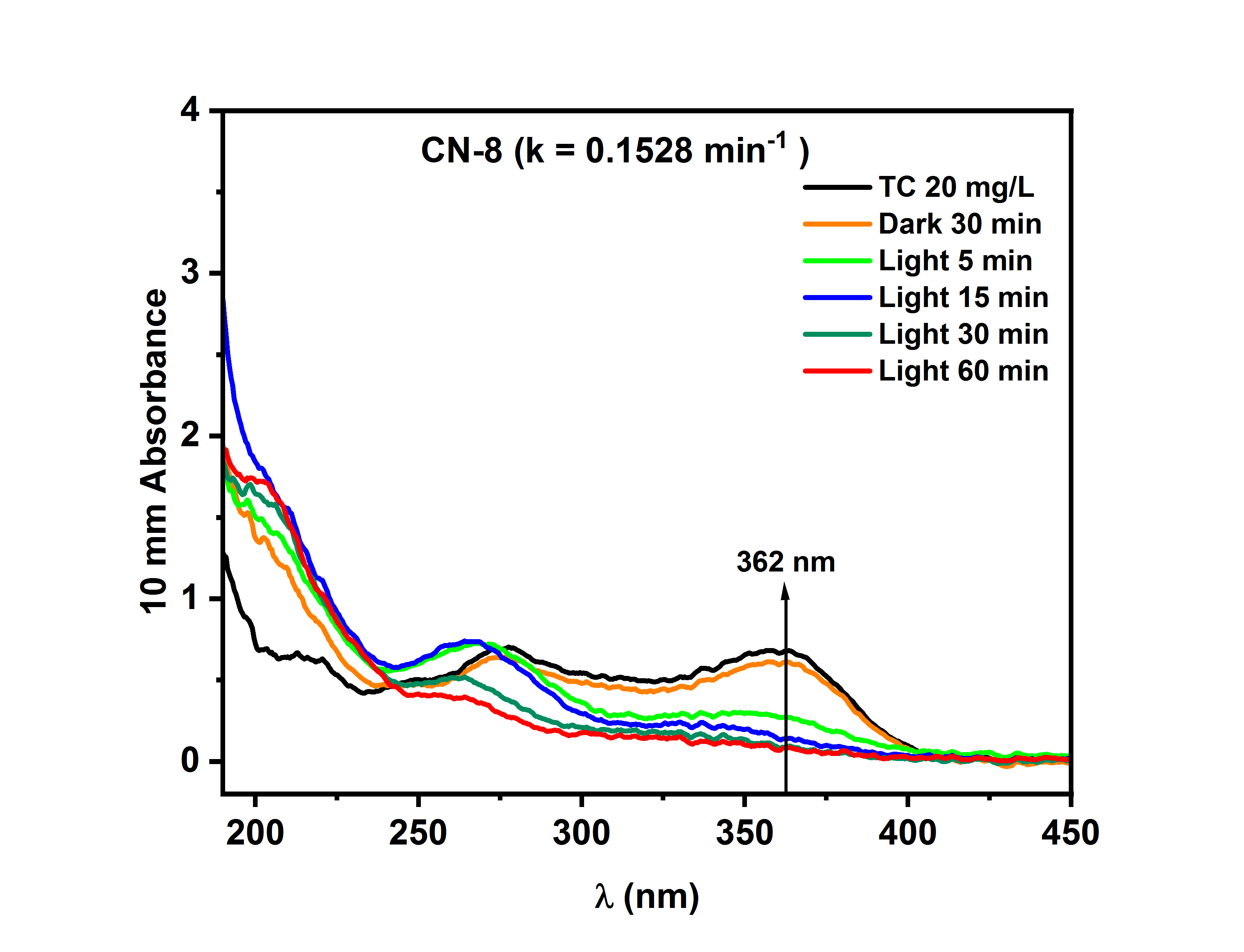


**a**

**b**

**c**

**Figure S10.** In situ monitoring of the degradation of TC by CN550, CN550-8, and CN-8.


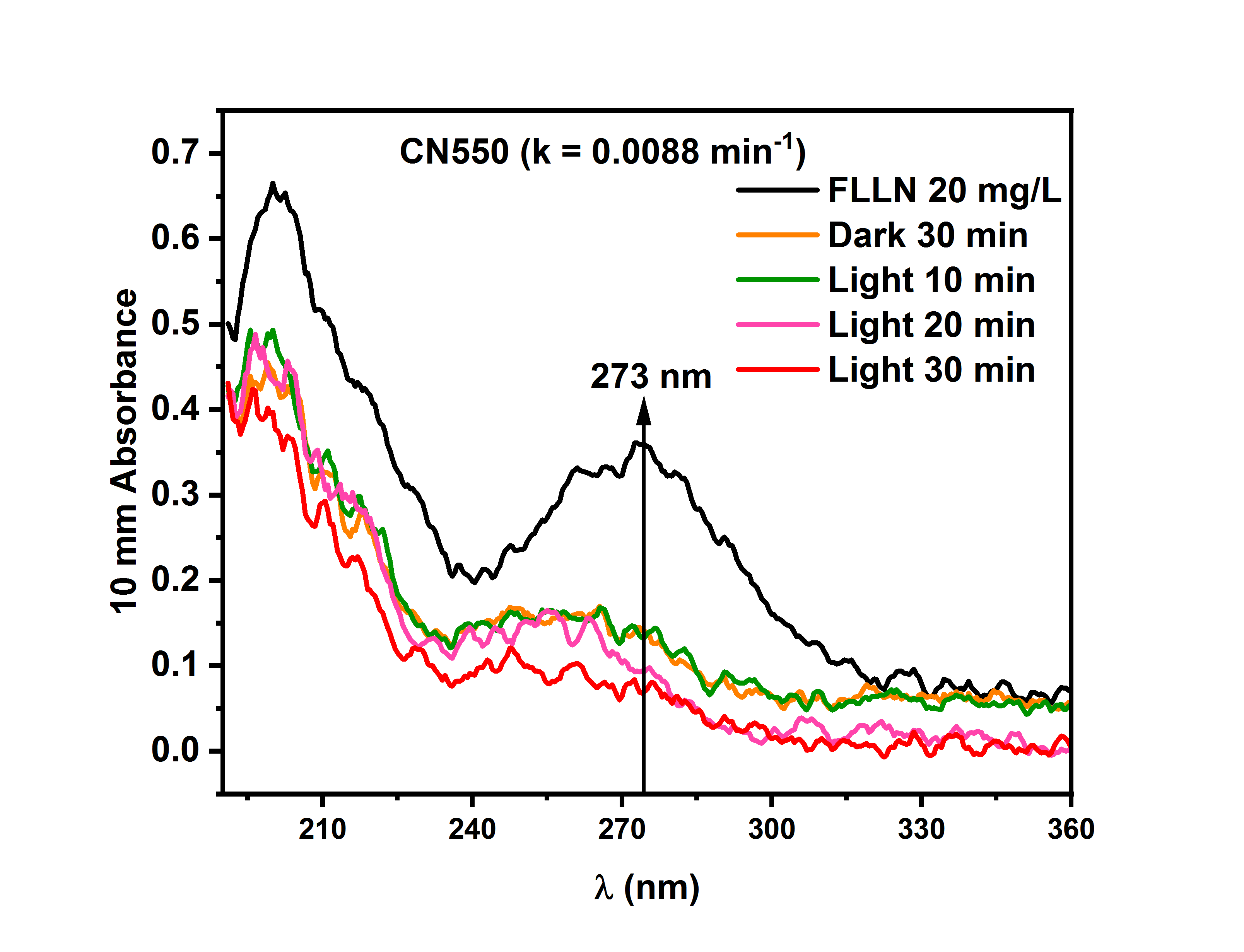

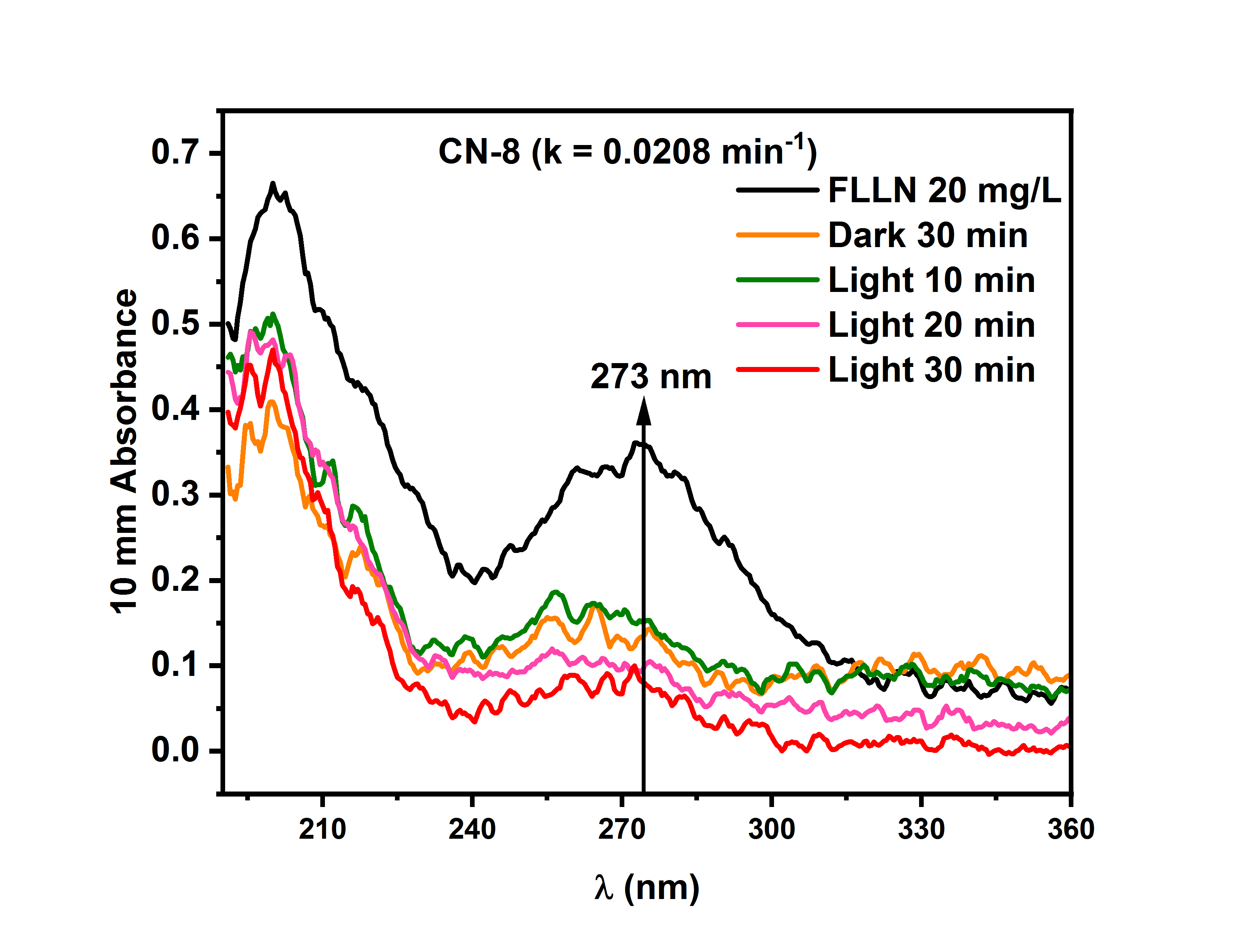

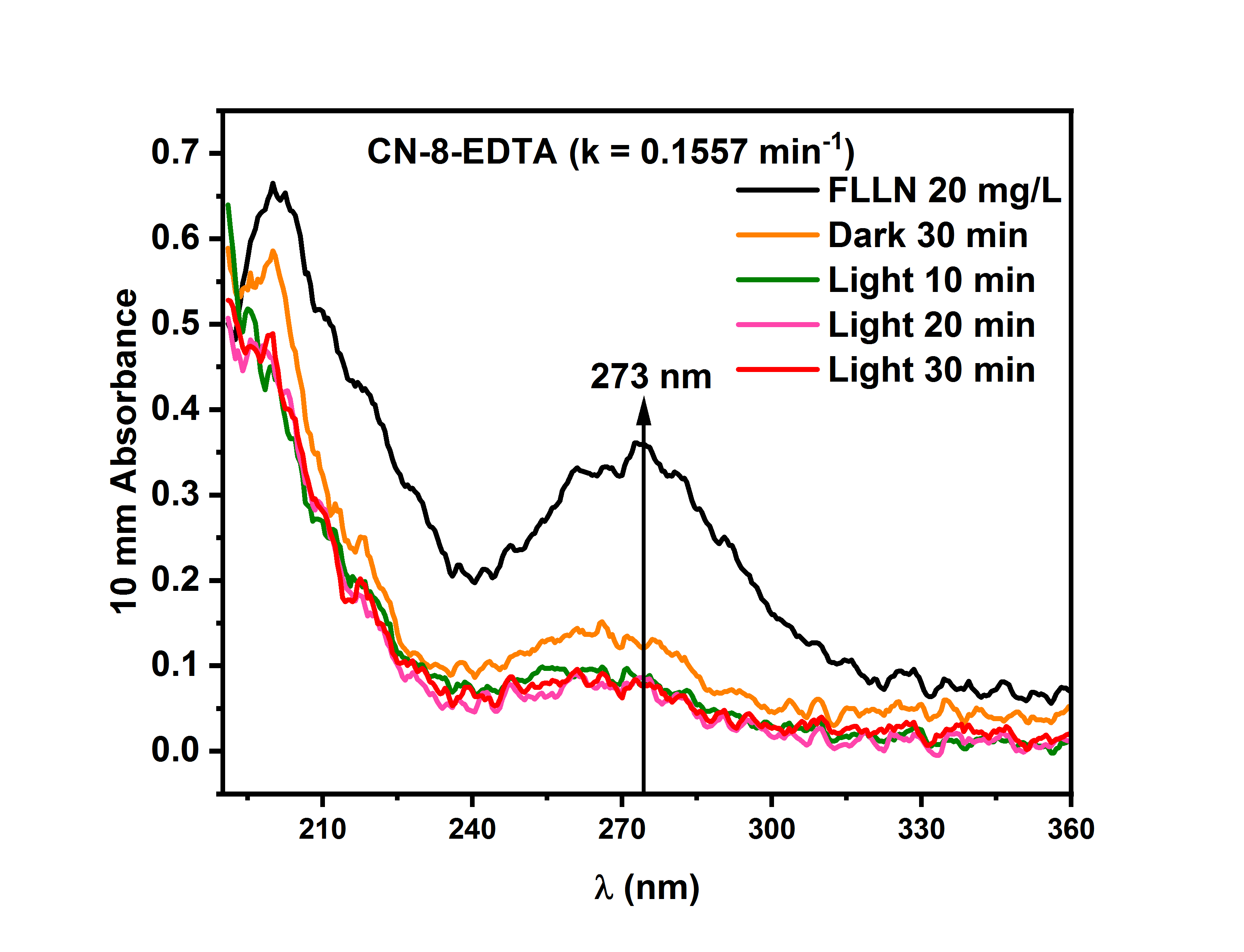


**a**

**c**

**b**

**Figure S11.** In situ monitoring of the degradation of FLLN by CN550, CN-8 and CN-8-EDTA.


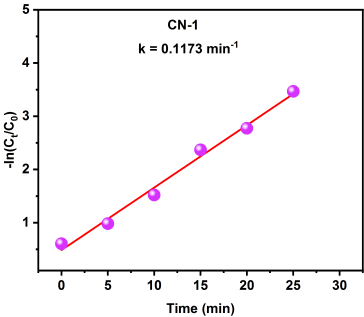

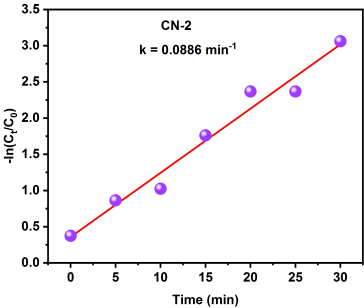

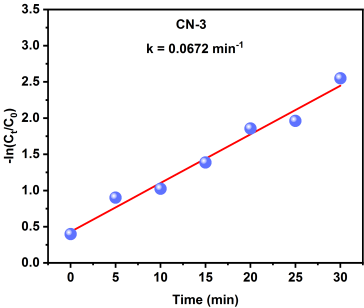

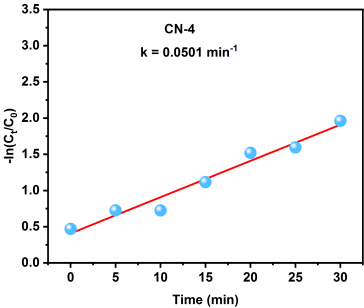

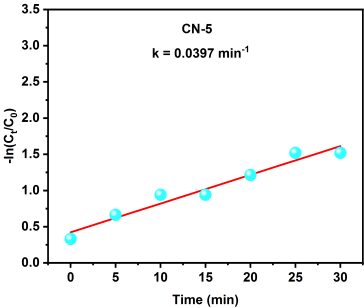

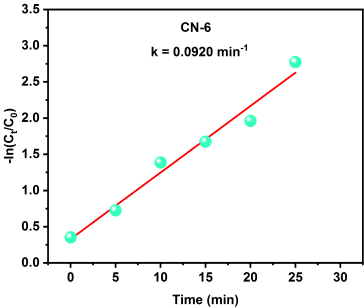

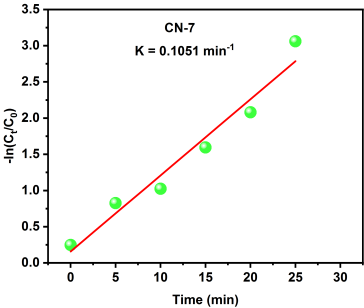

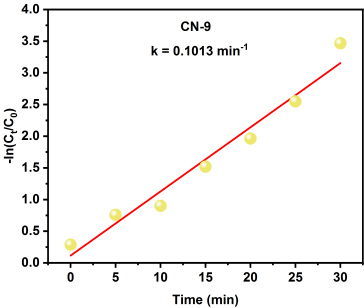

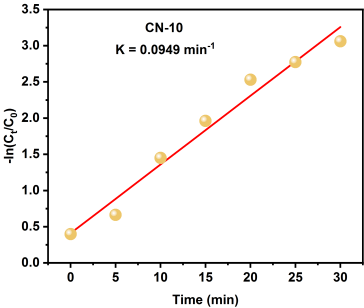

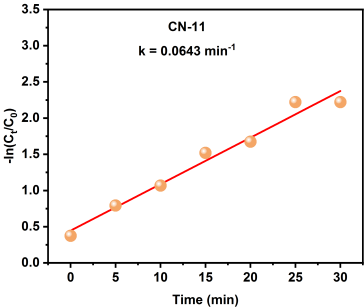

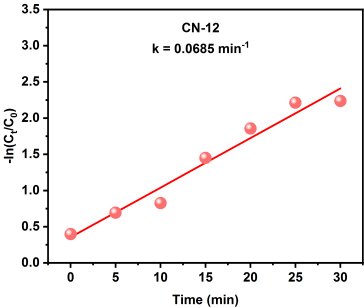

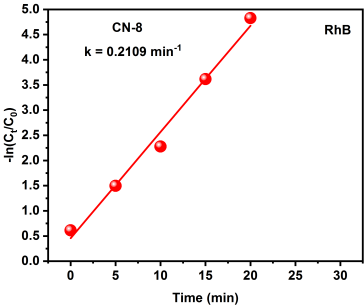


**Figure S12.** The degradation rate of RhB is iteratively fitted until at least 95% of the data matches the model (R^2^ > 0.95).


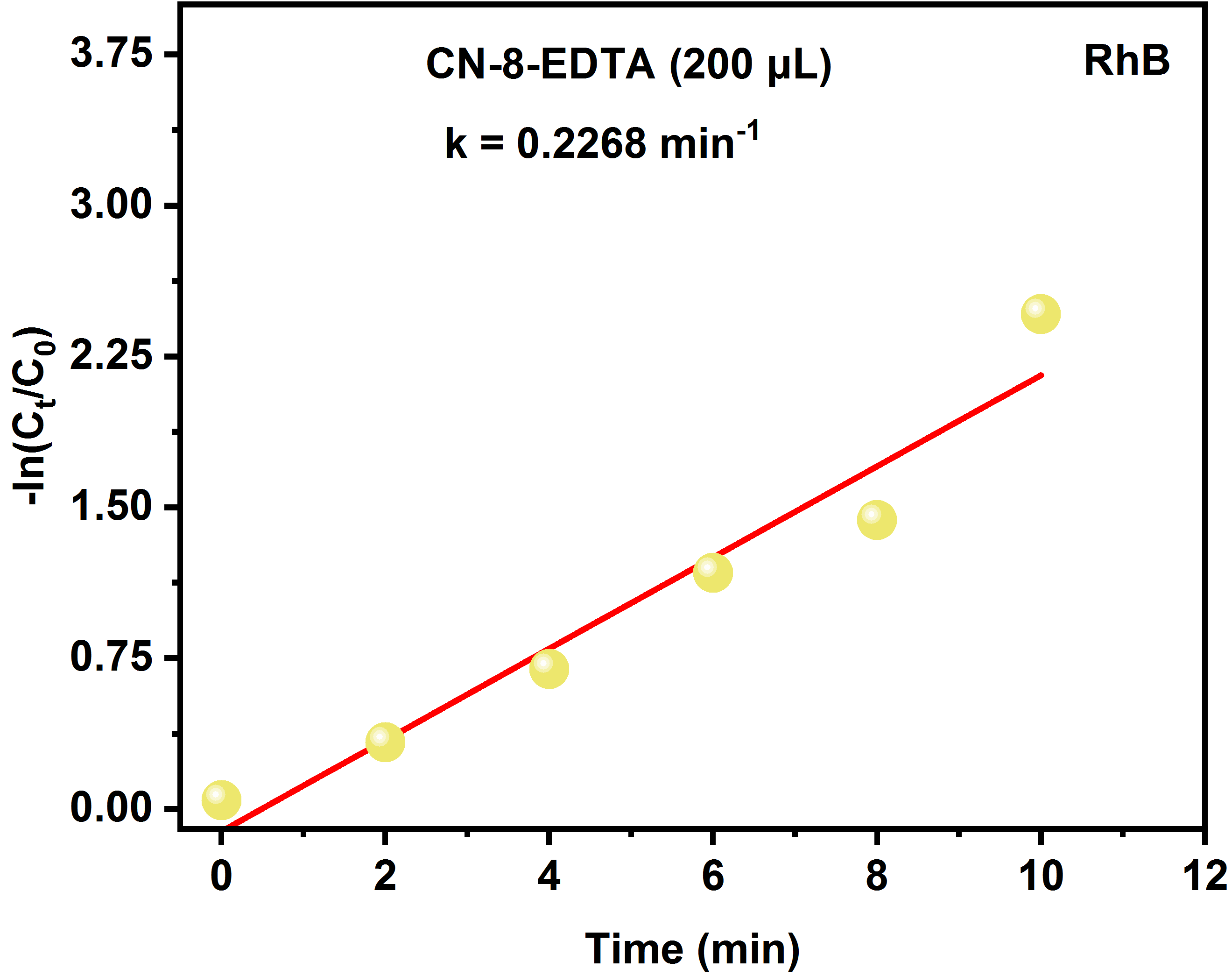

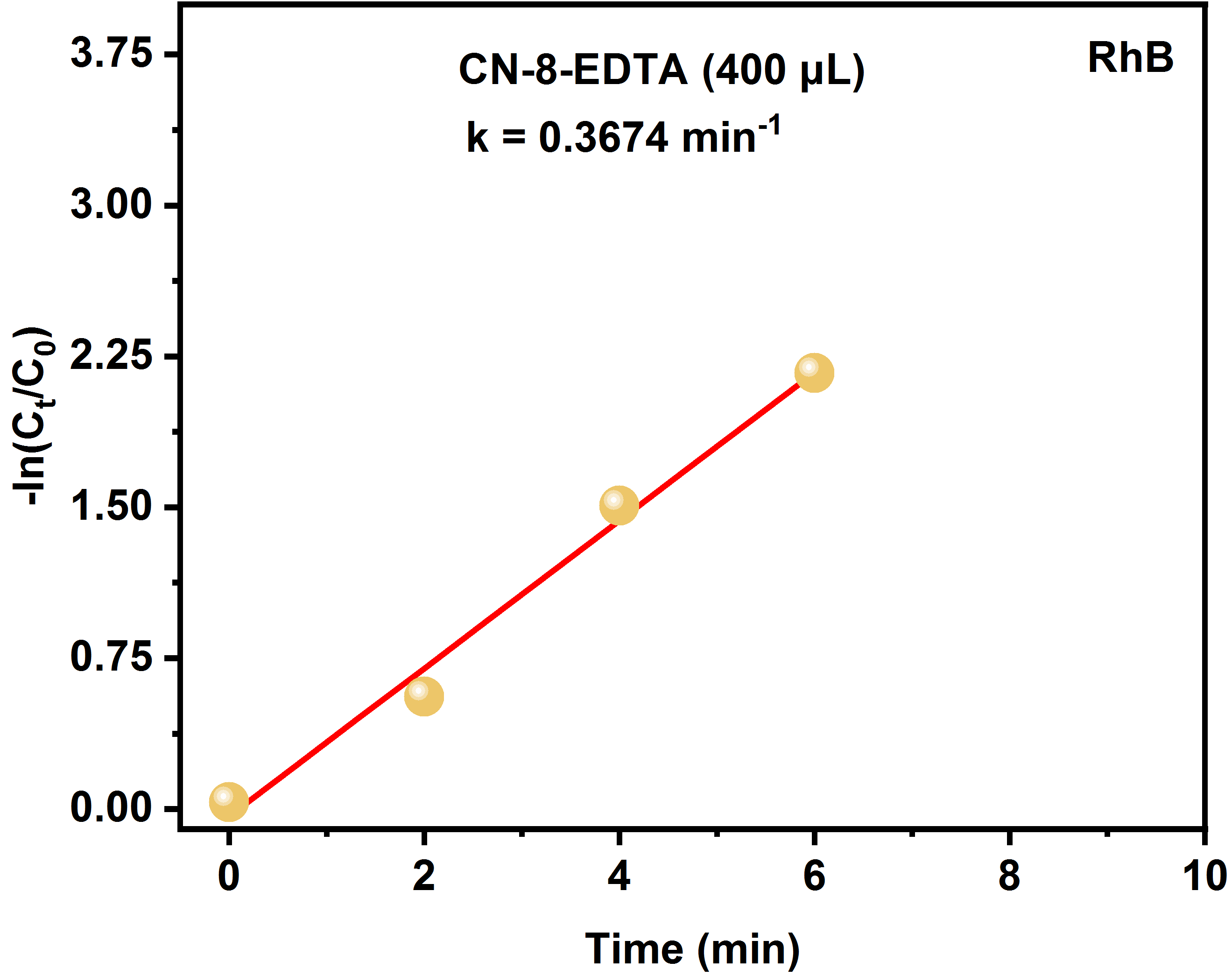

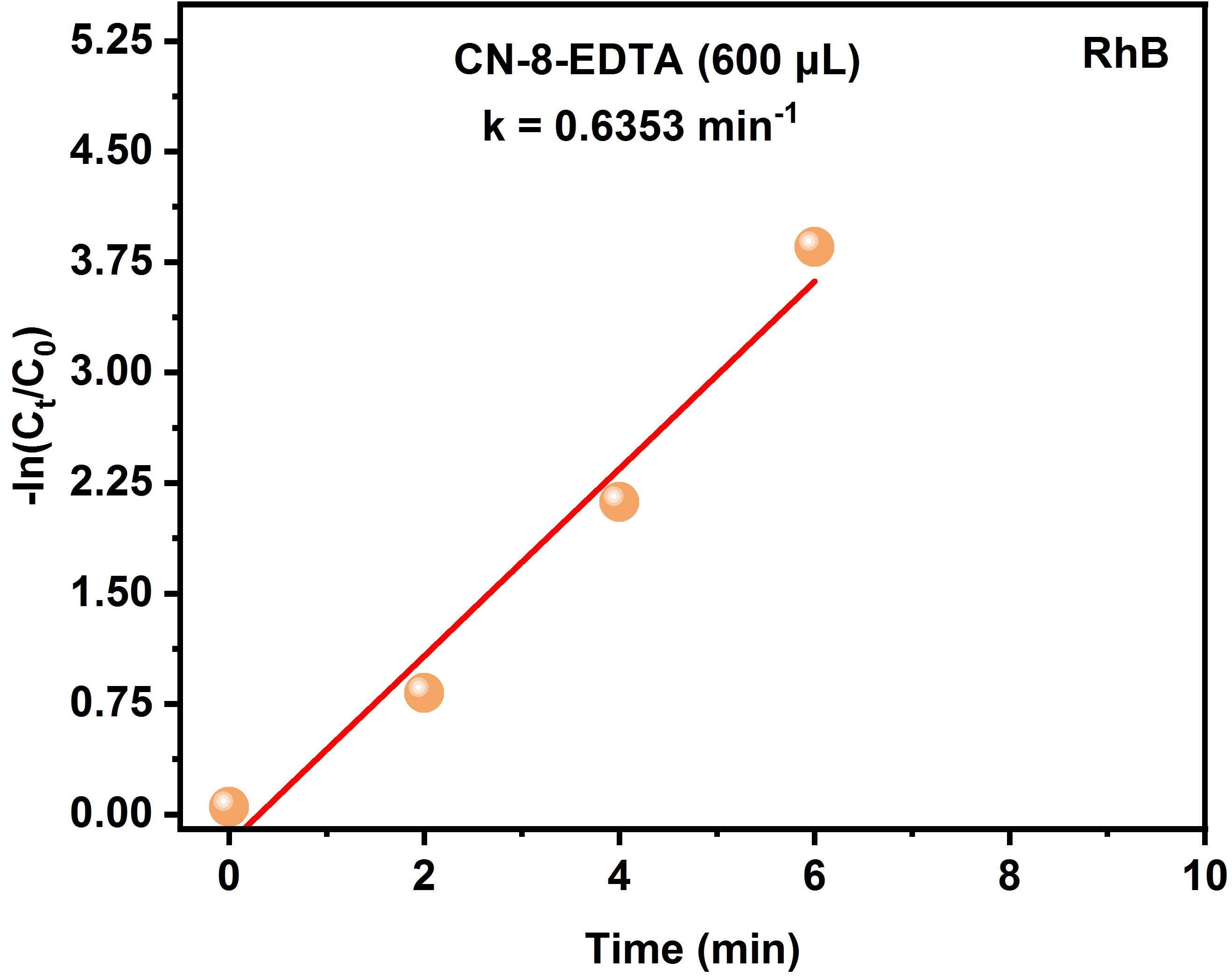

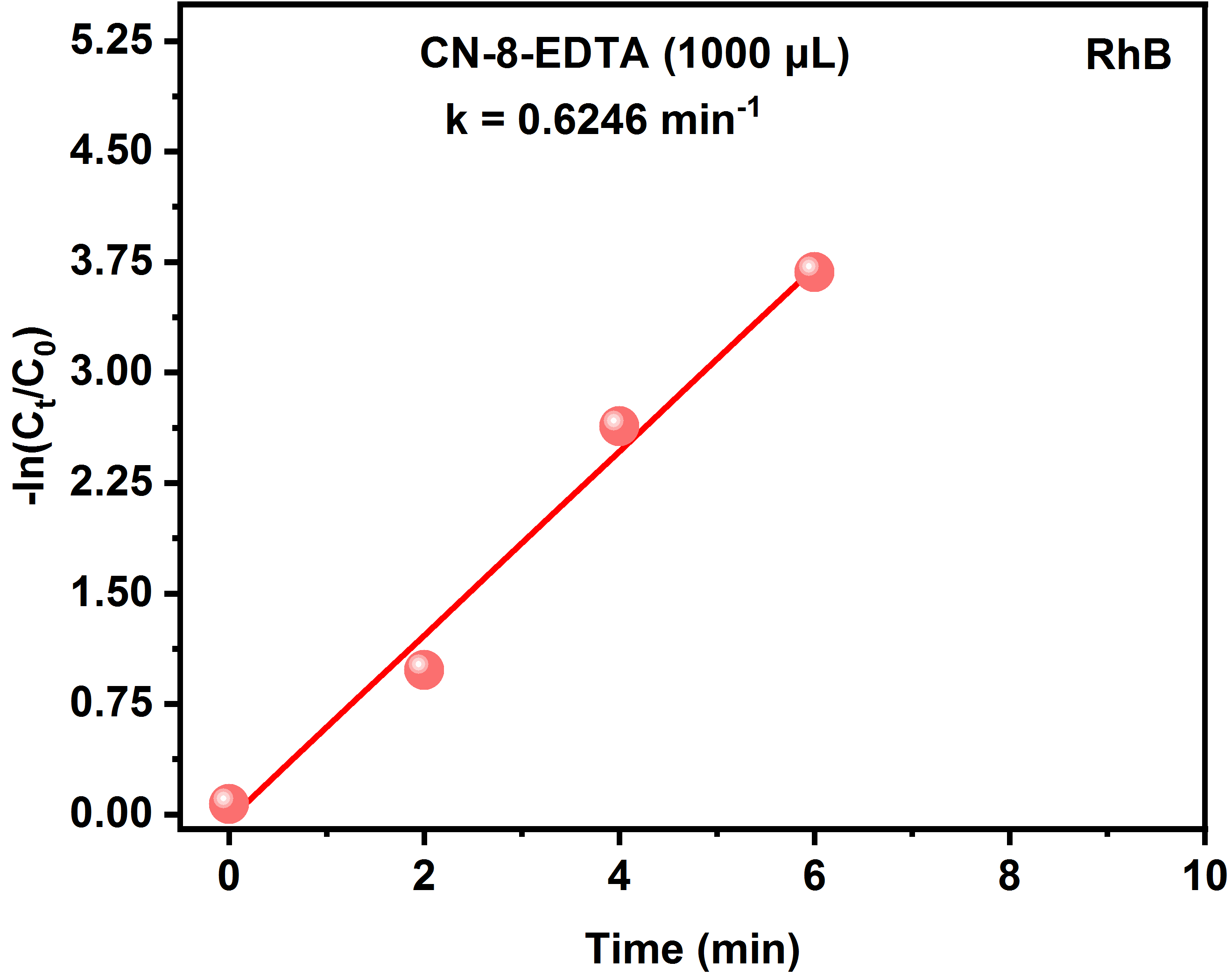


**Figure S13.** The degradation rate of CN-8-EDTA is iteratively fitted until at least 95% of the data matches the model (R^2^ > 0.95).


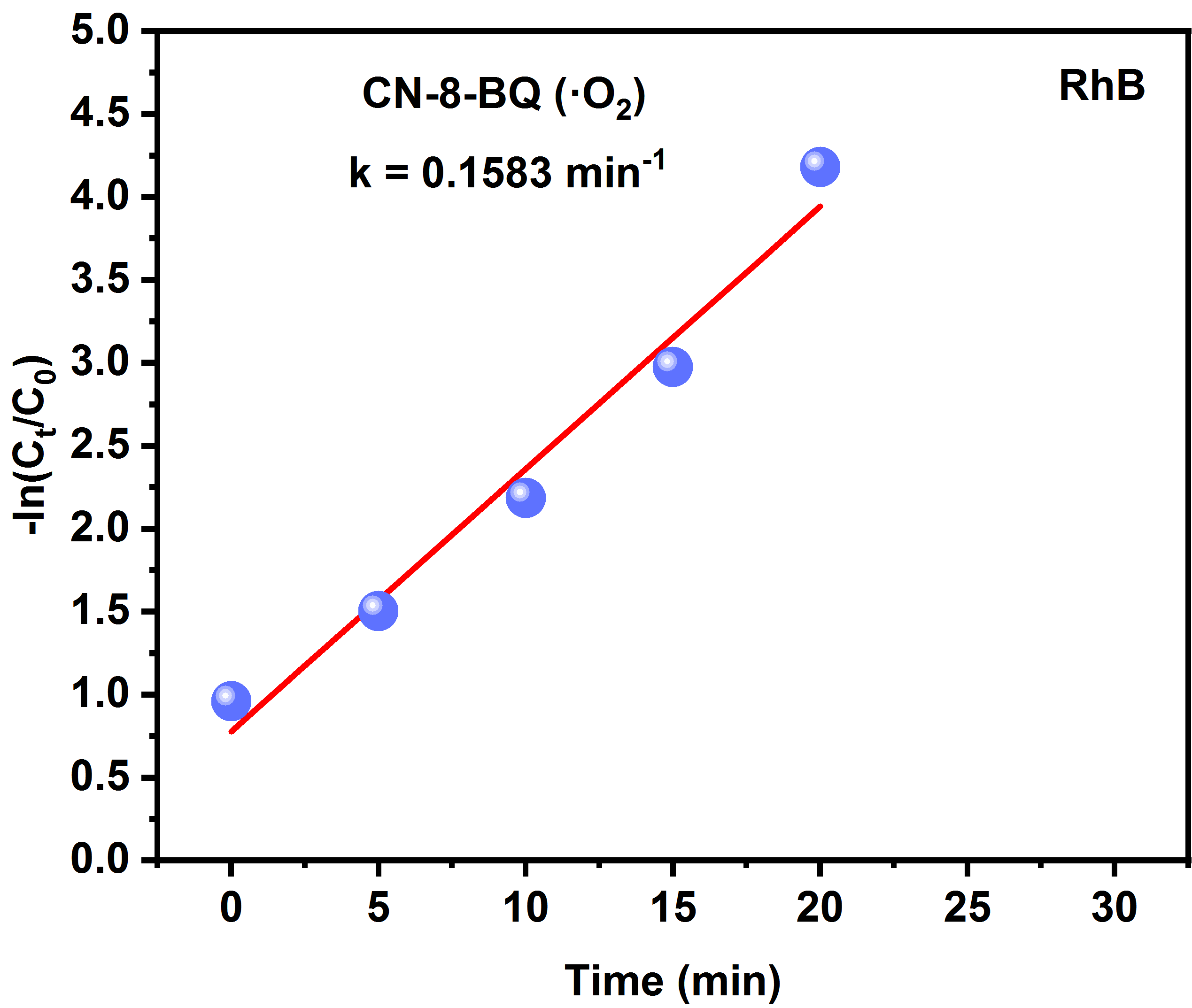

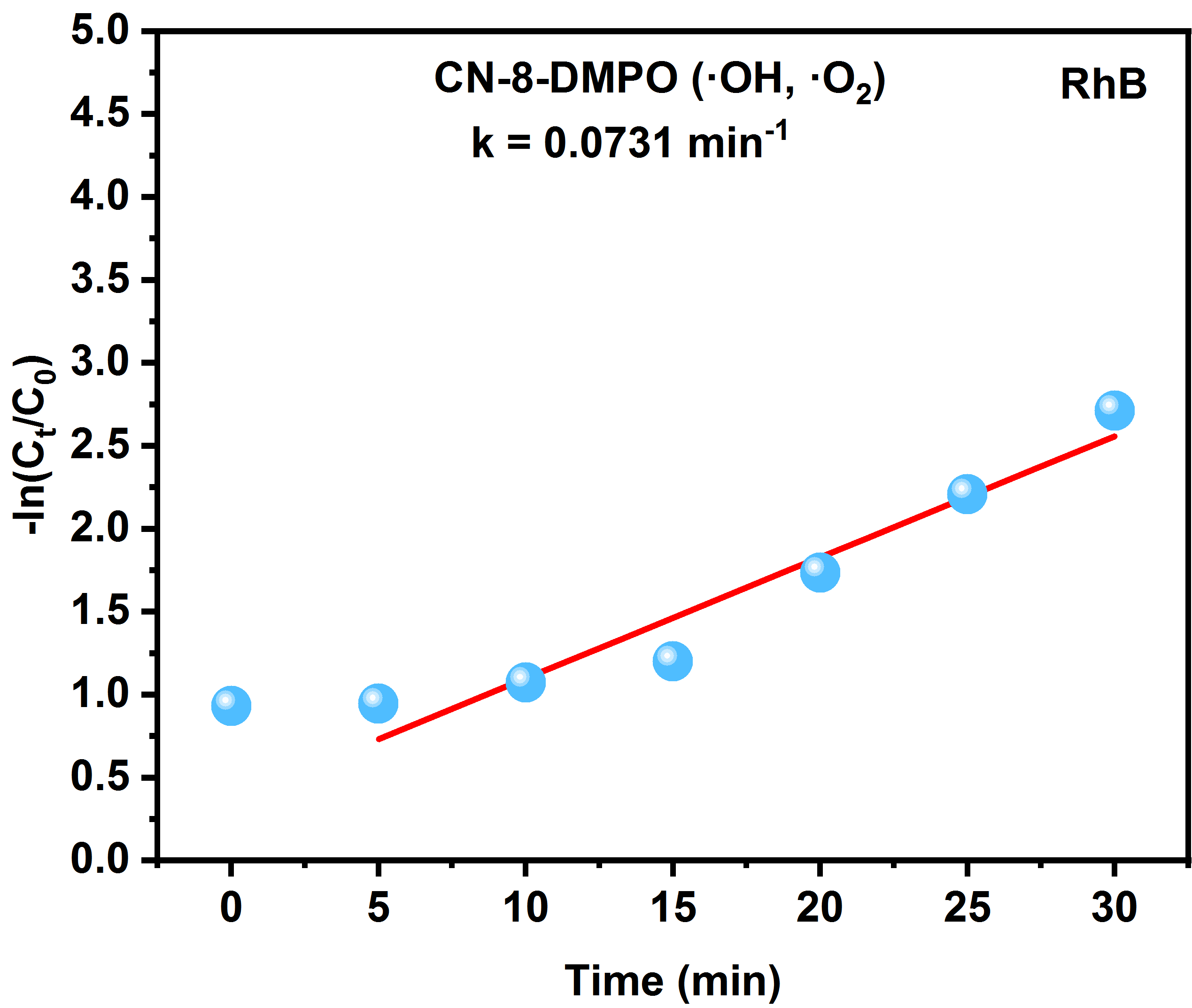

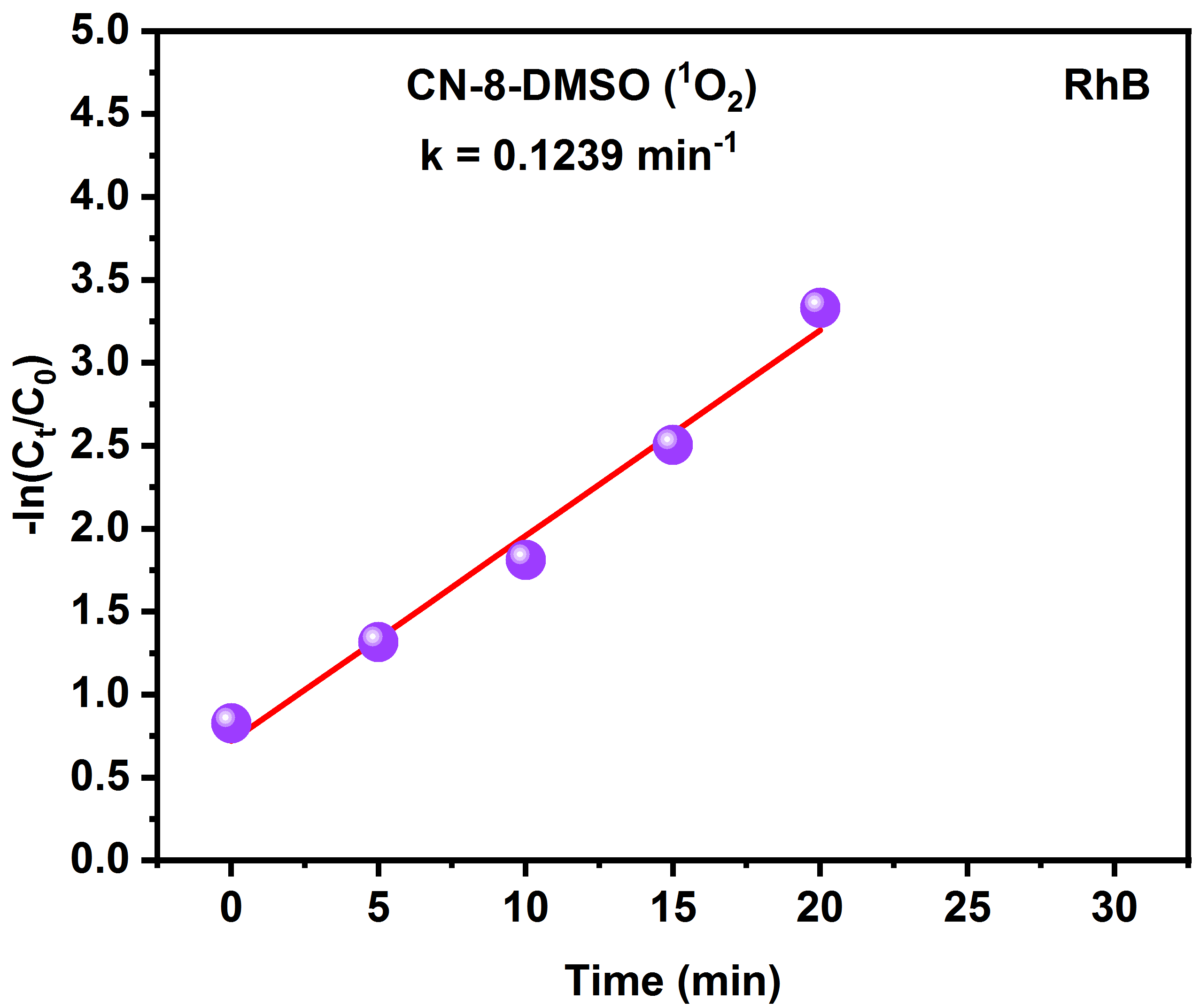

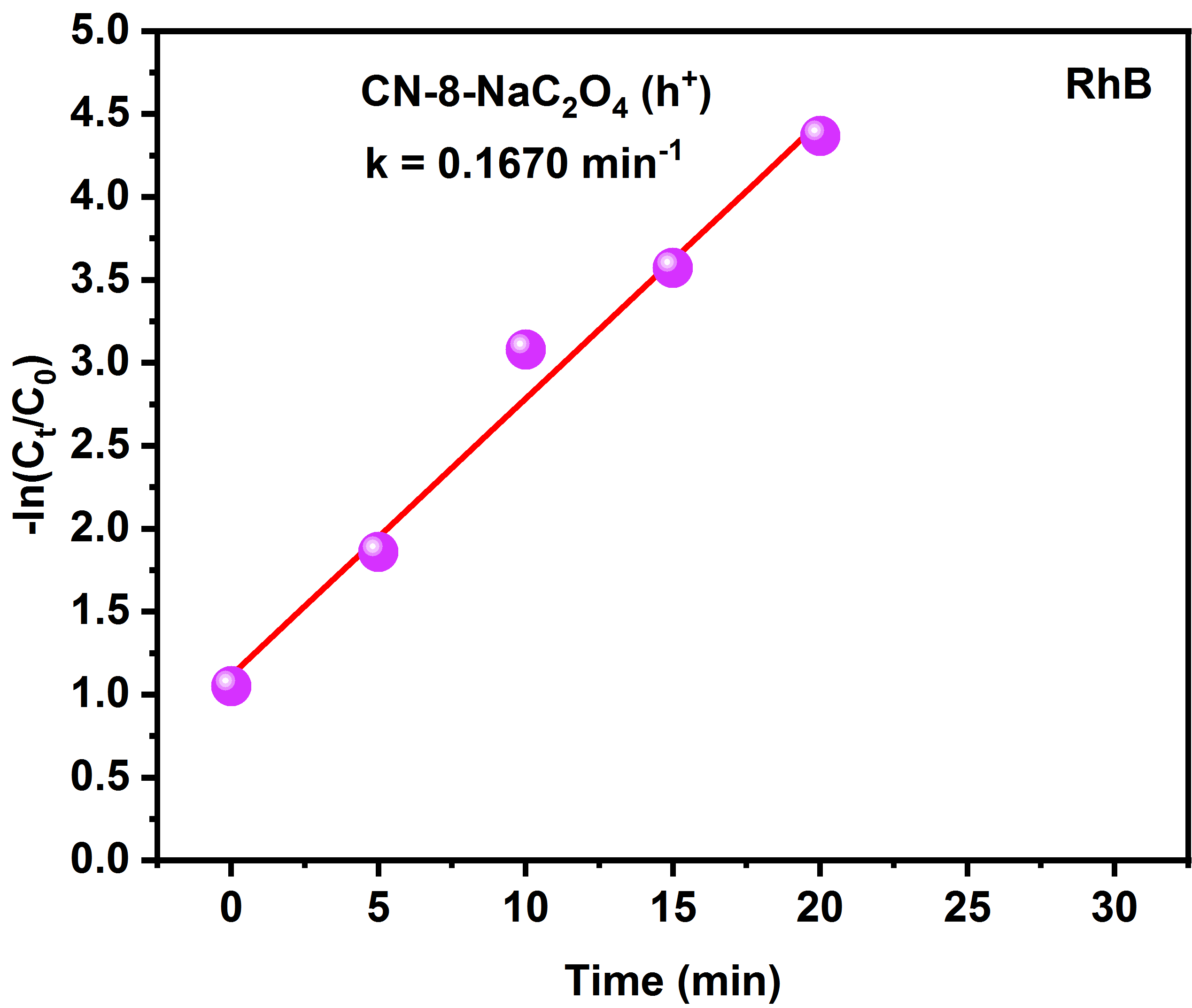


**Figure S14.** The degradation rate of free radical capture of CN-8 is iteratively fitted until at least 95% of the data matches the model (R^2^ > 0.95).


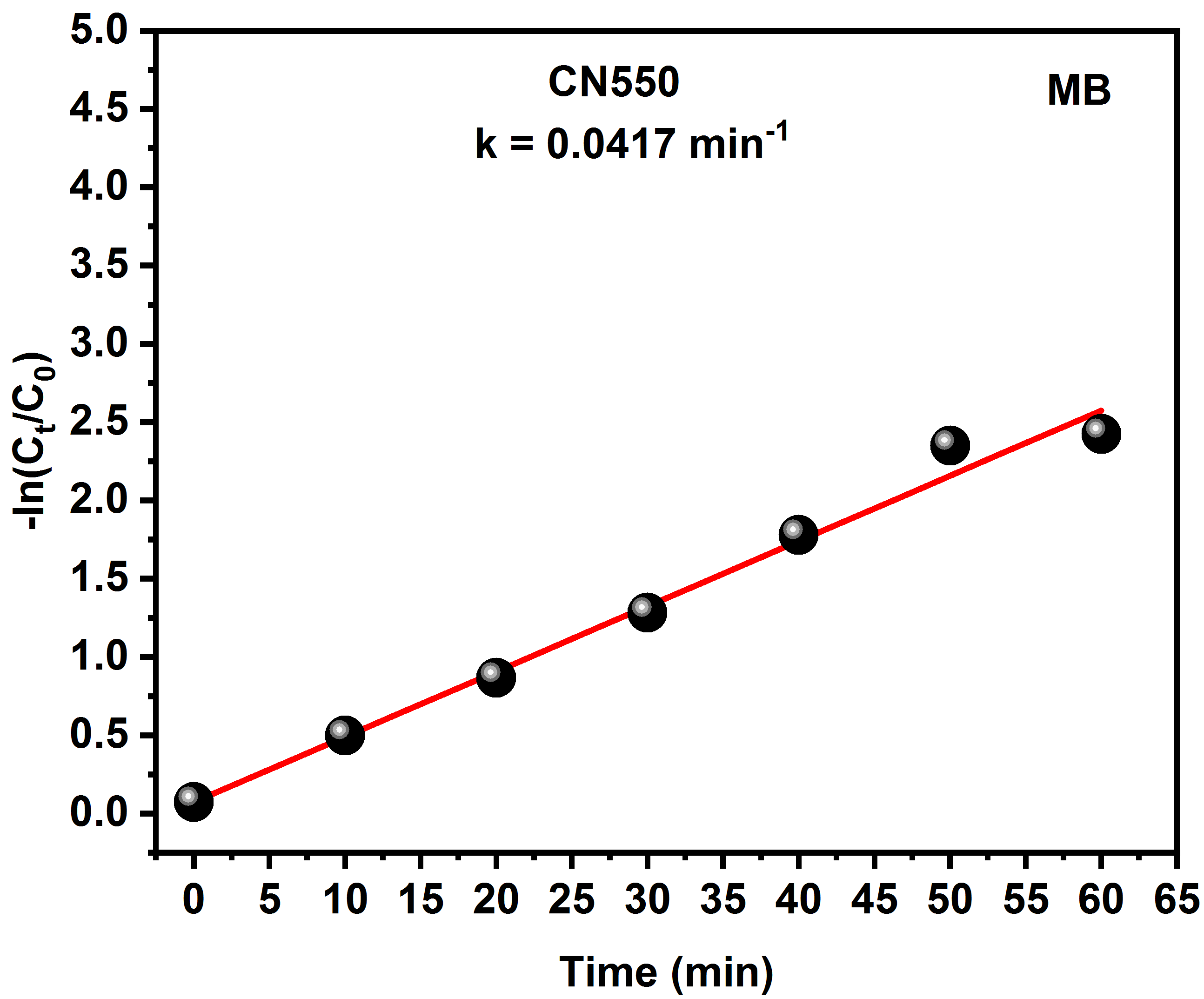

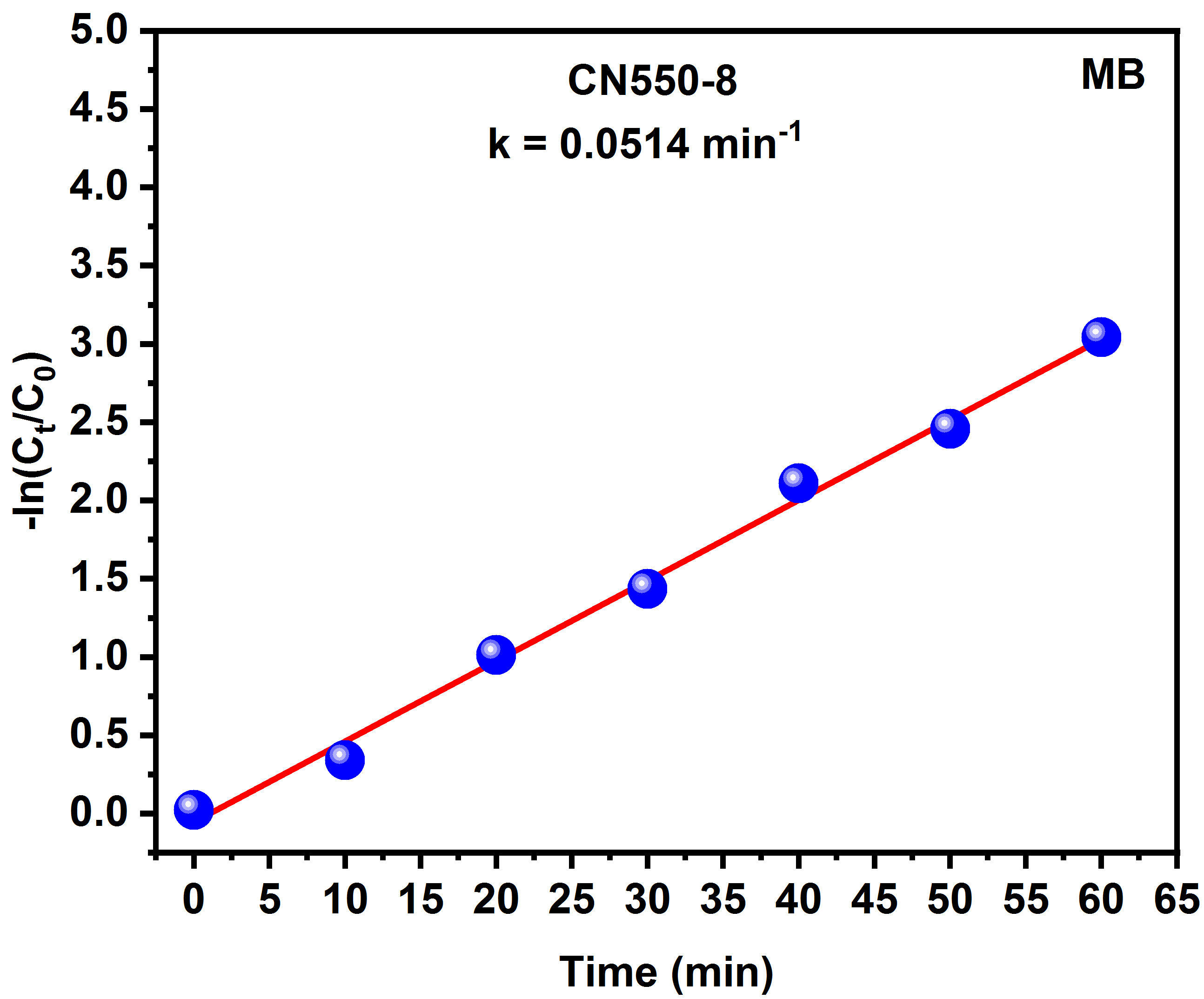

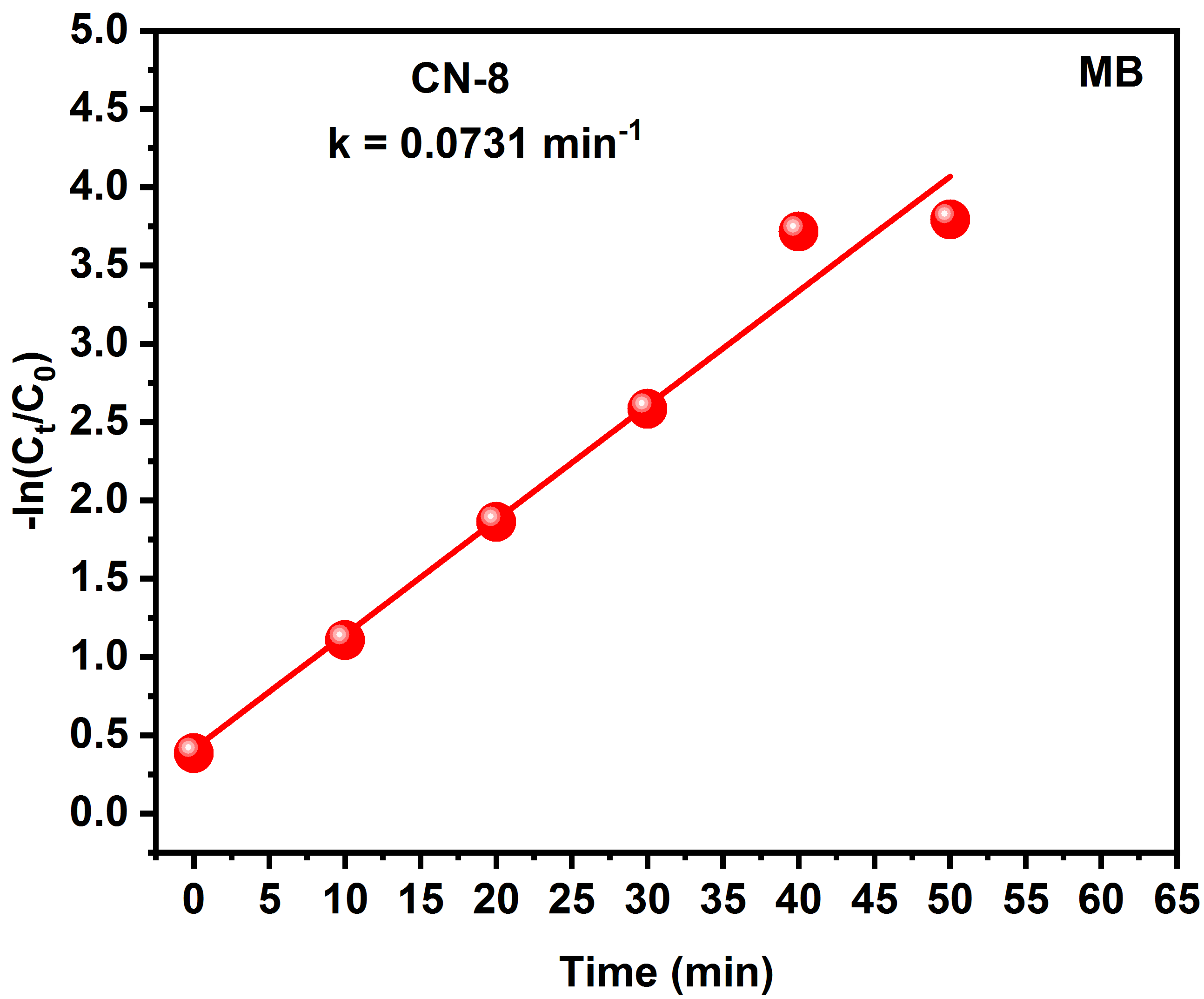

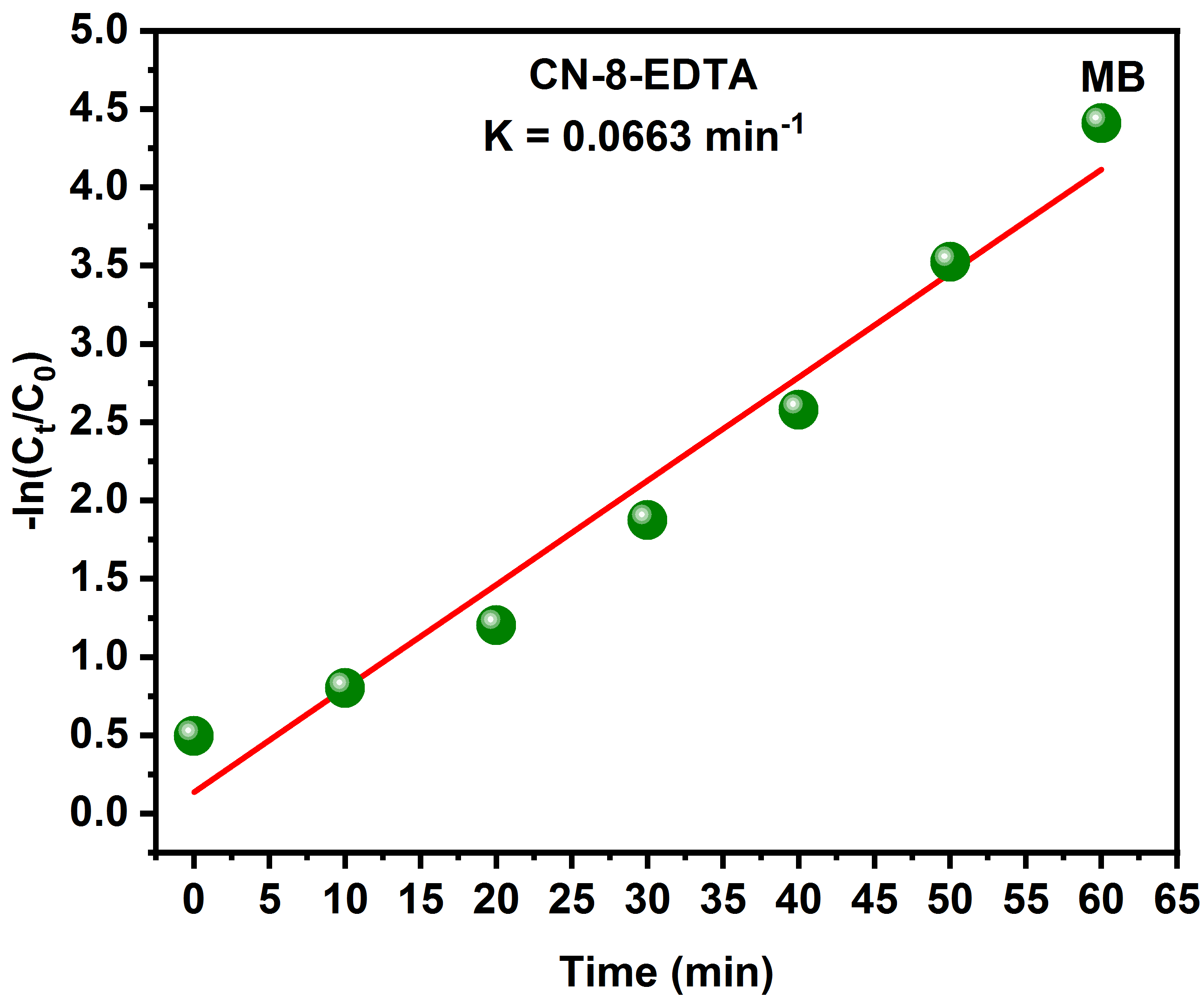


**Figure S15.** The degradation rate of MB is iteratively fitted until at least 95% of the data matches the model (R^2^ > 0.95).


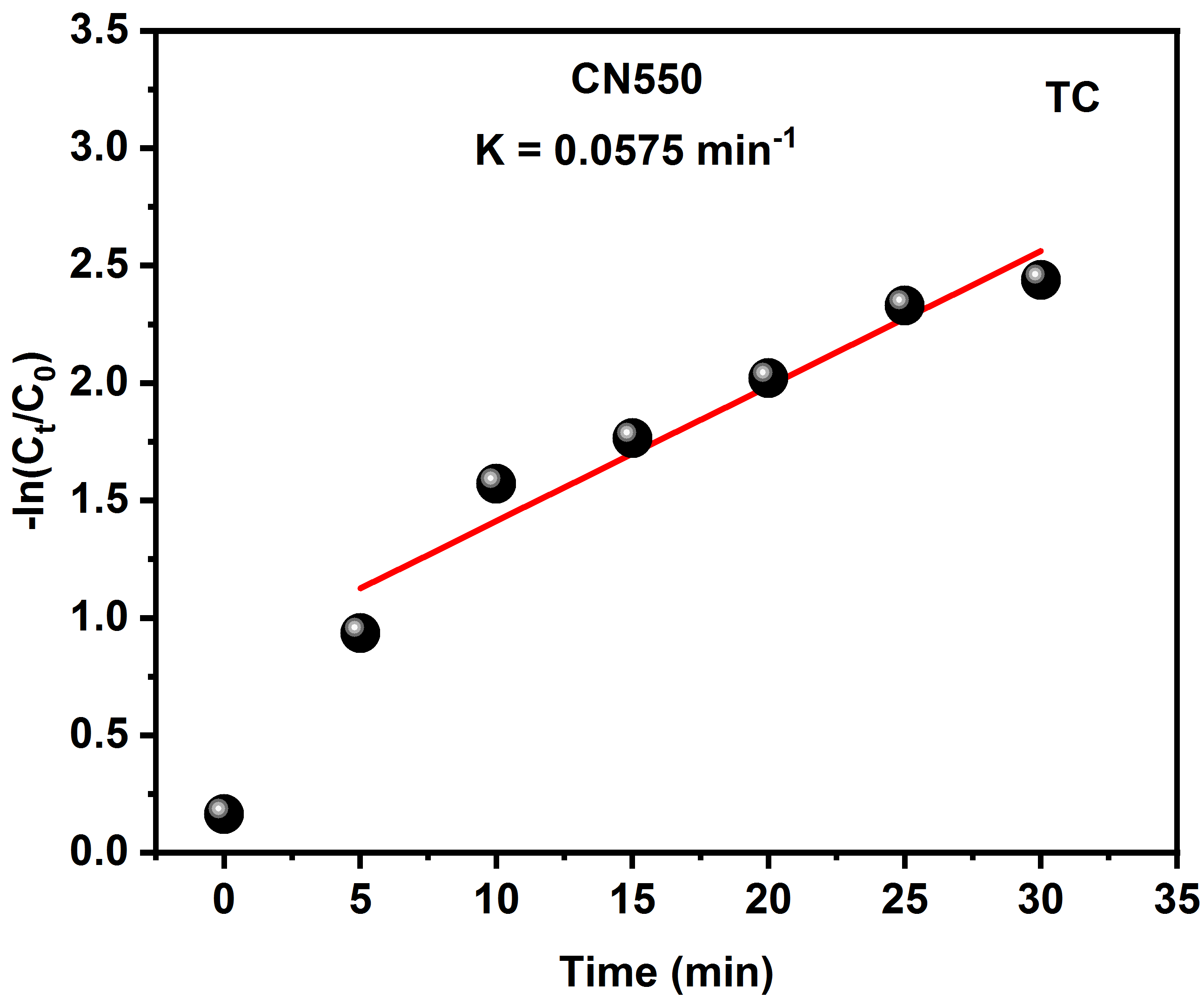

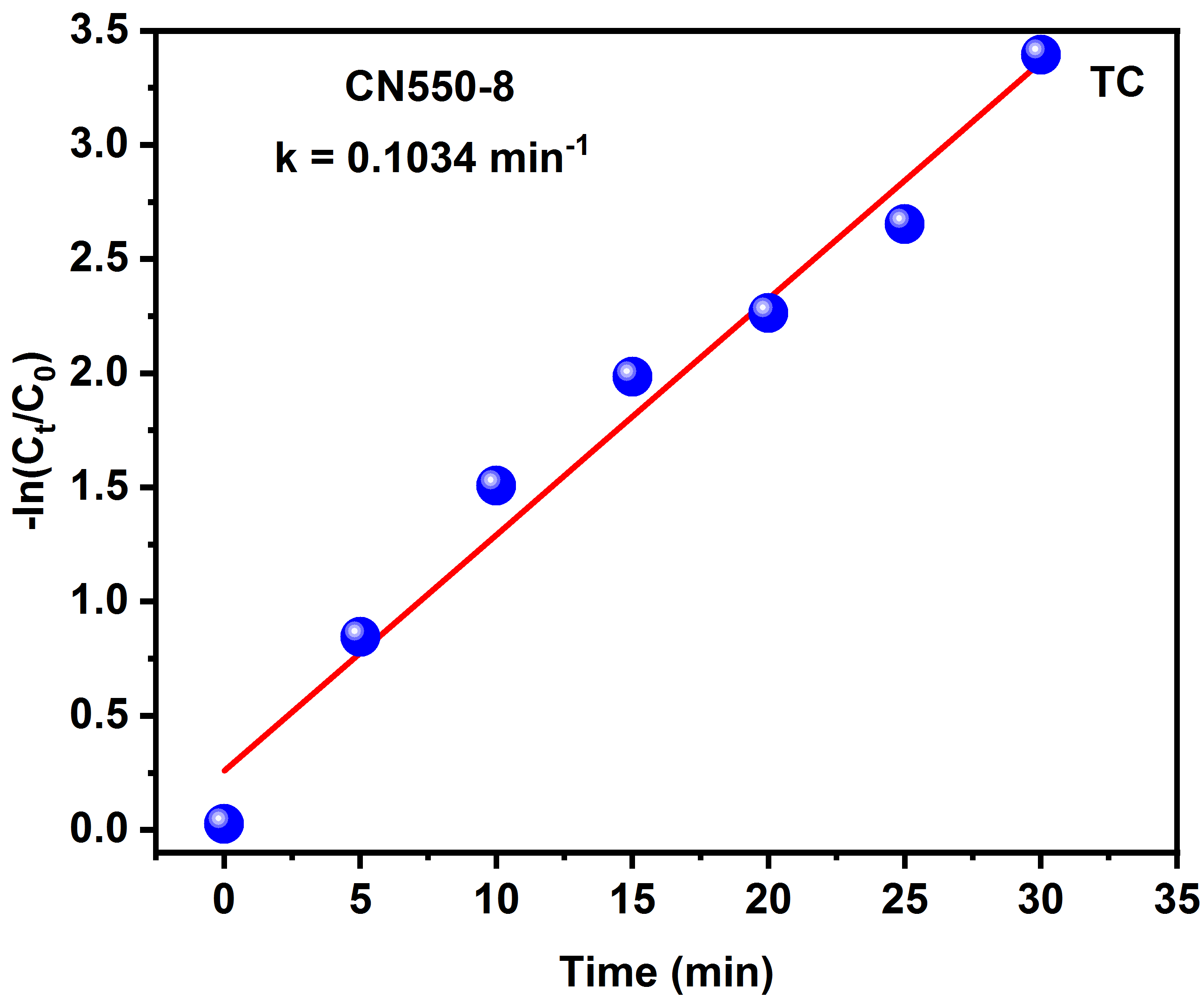

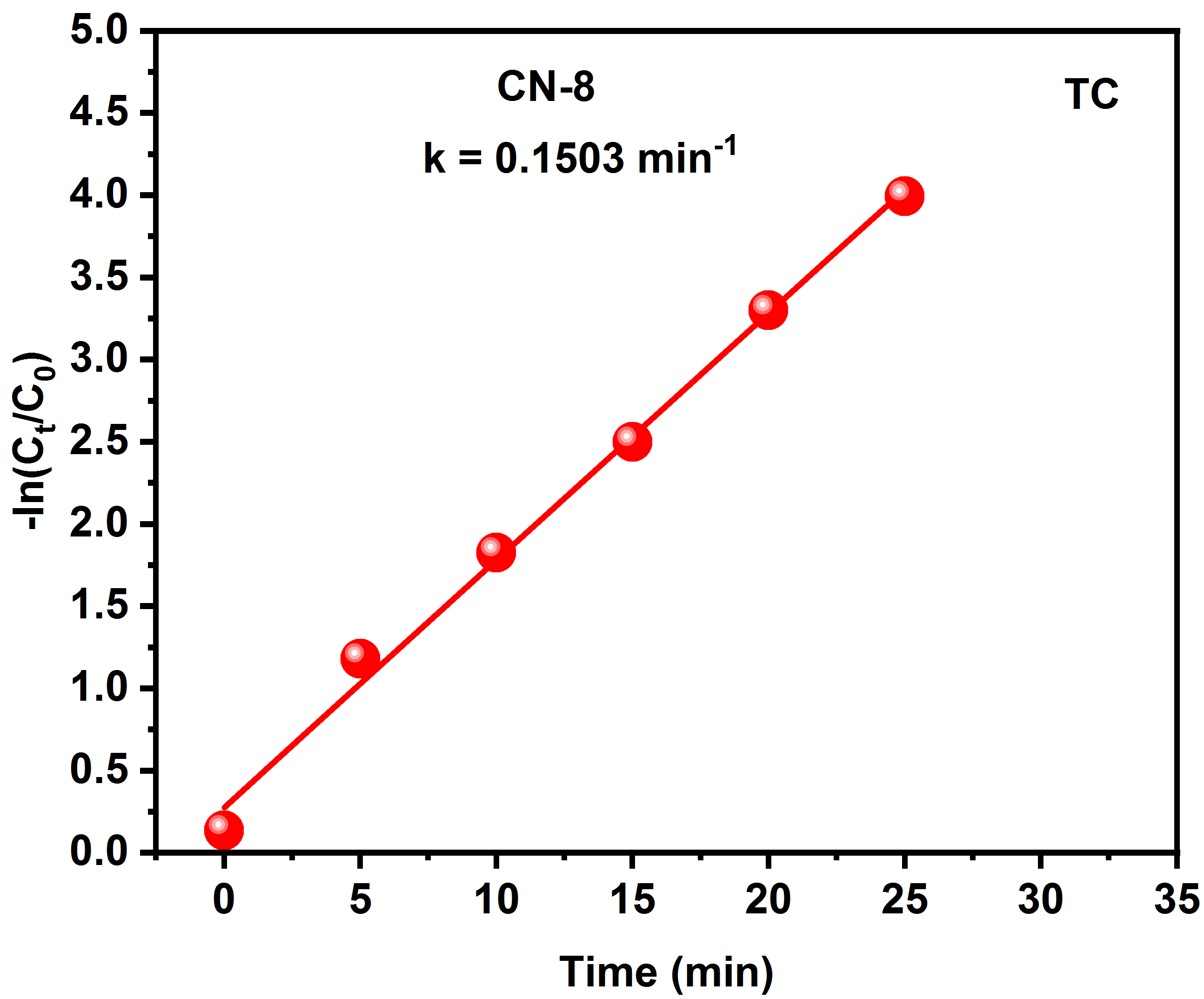

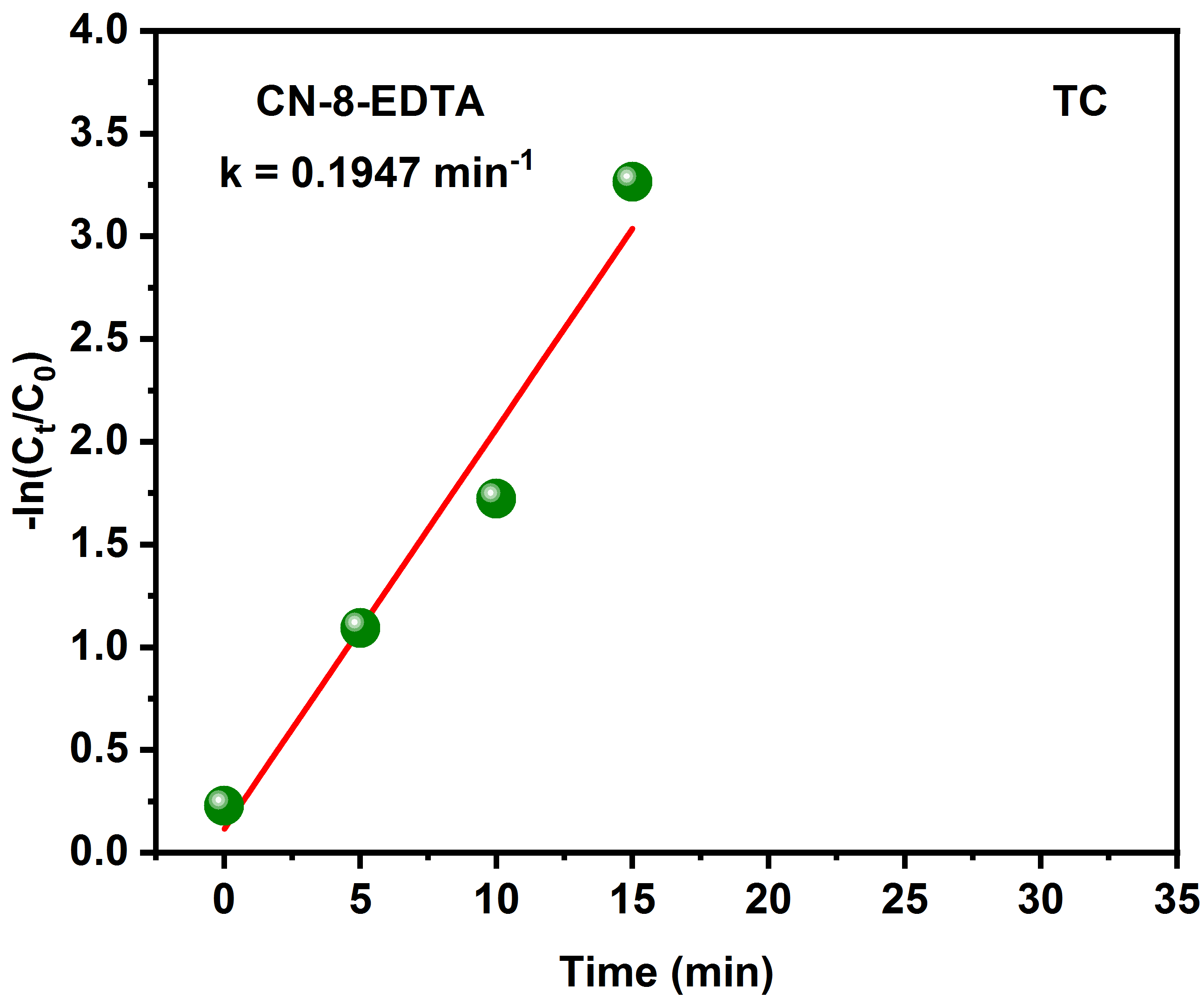


**Figure S16.** The degradation rate of TC is iteratively fitted until at least 95% of the data matches the model (R^2^ > 0.95).


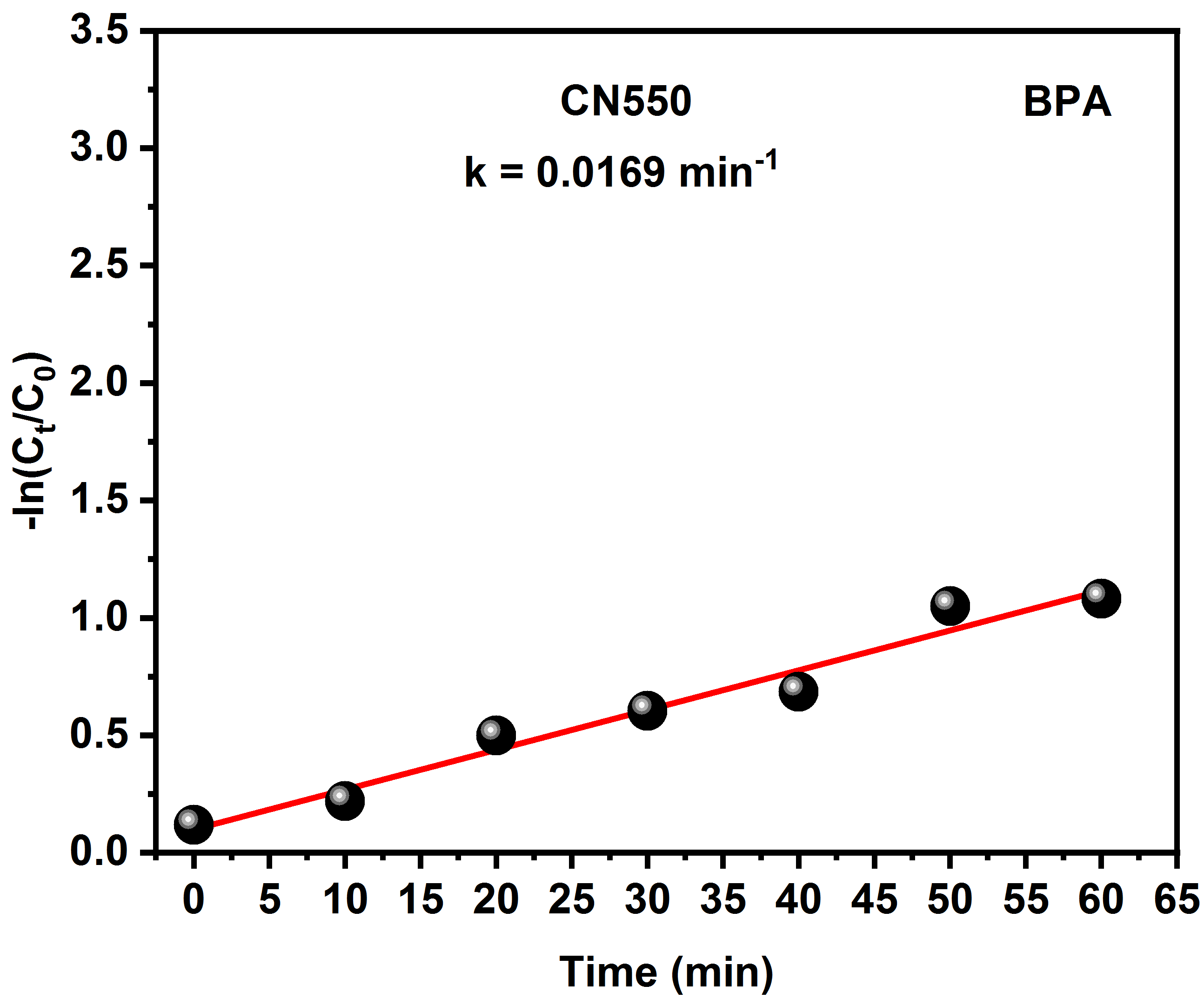

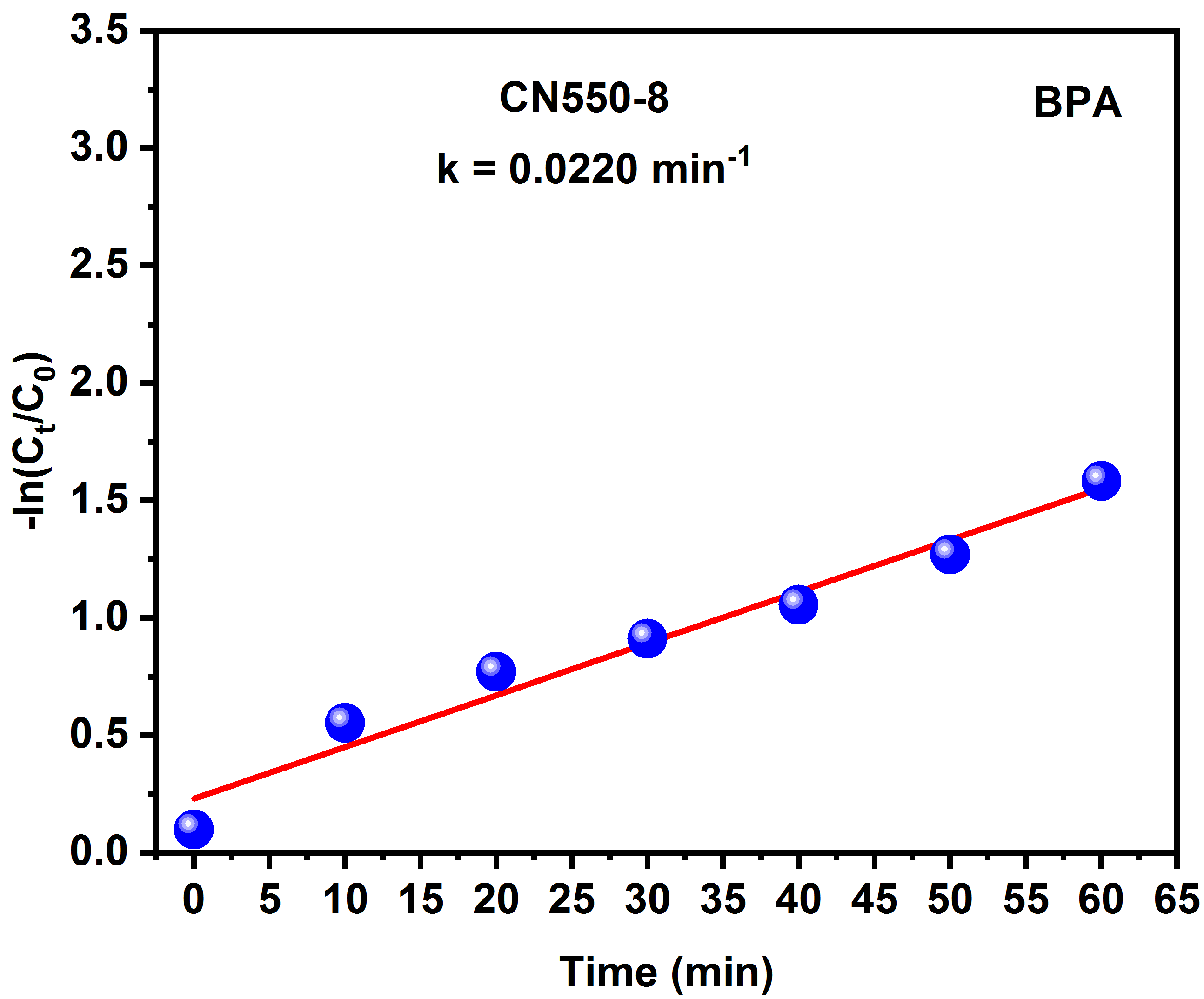

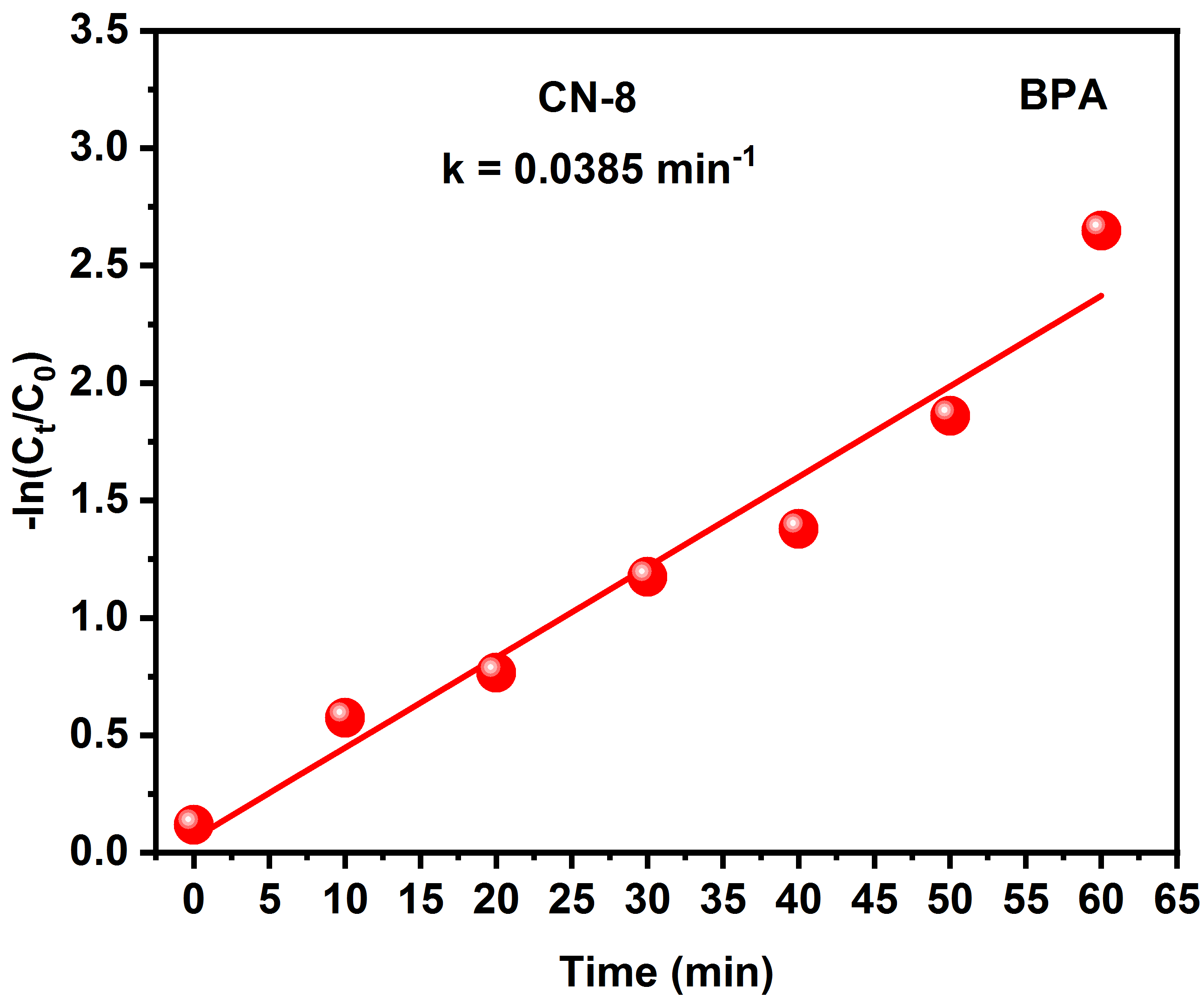

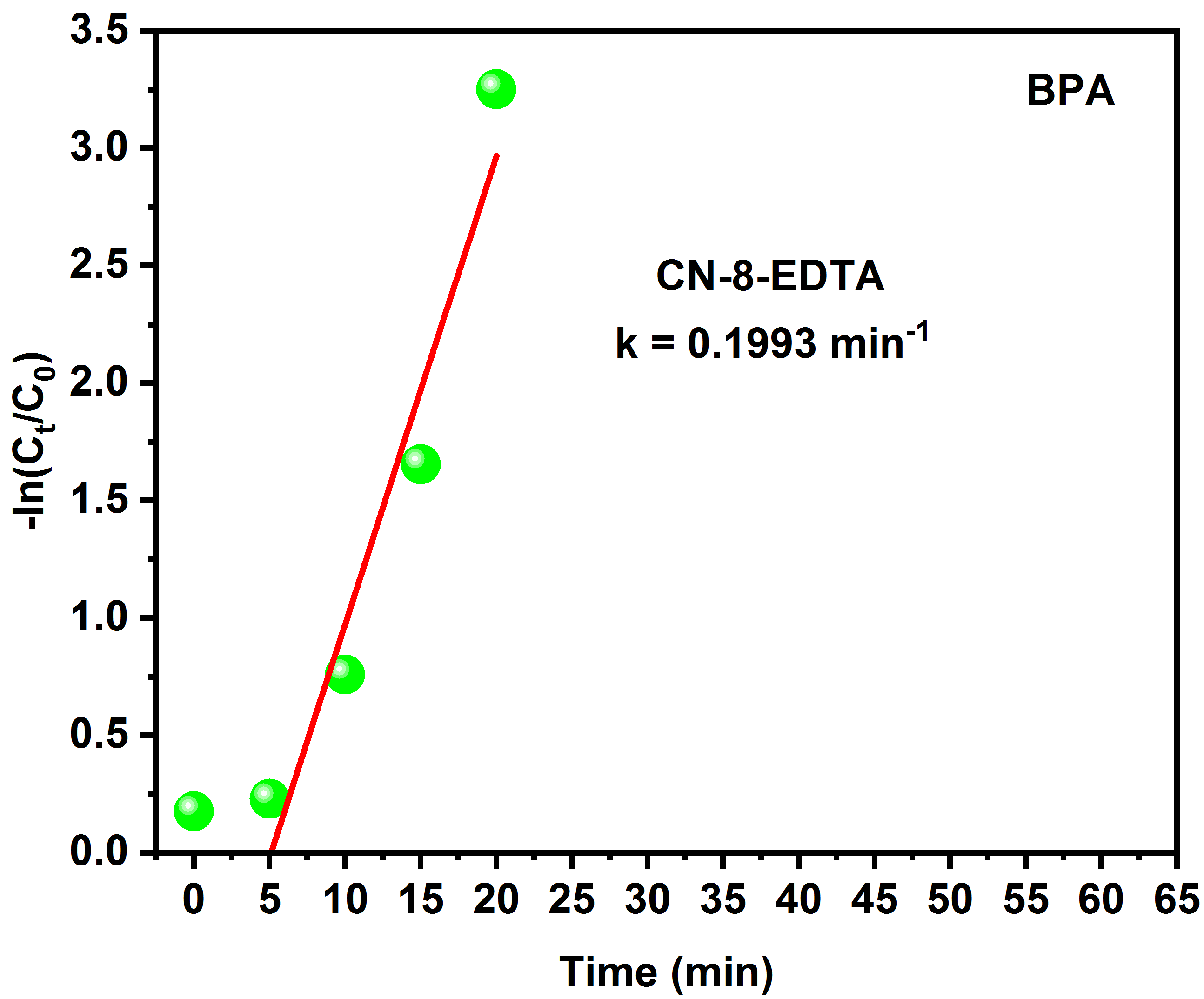


**Figure S17.** The degradation rate of BPA is iteratively fitted until at least 95% of the data matches the model (R^2^ > 0.95).


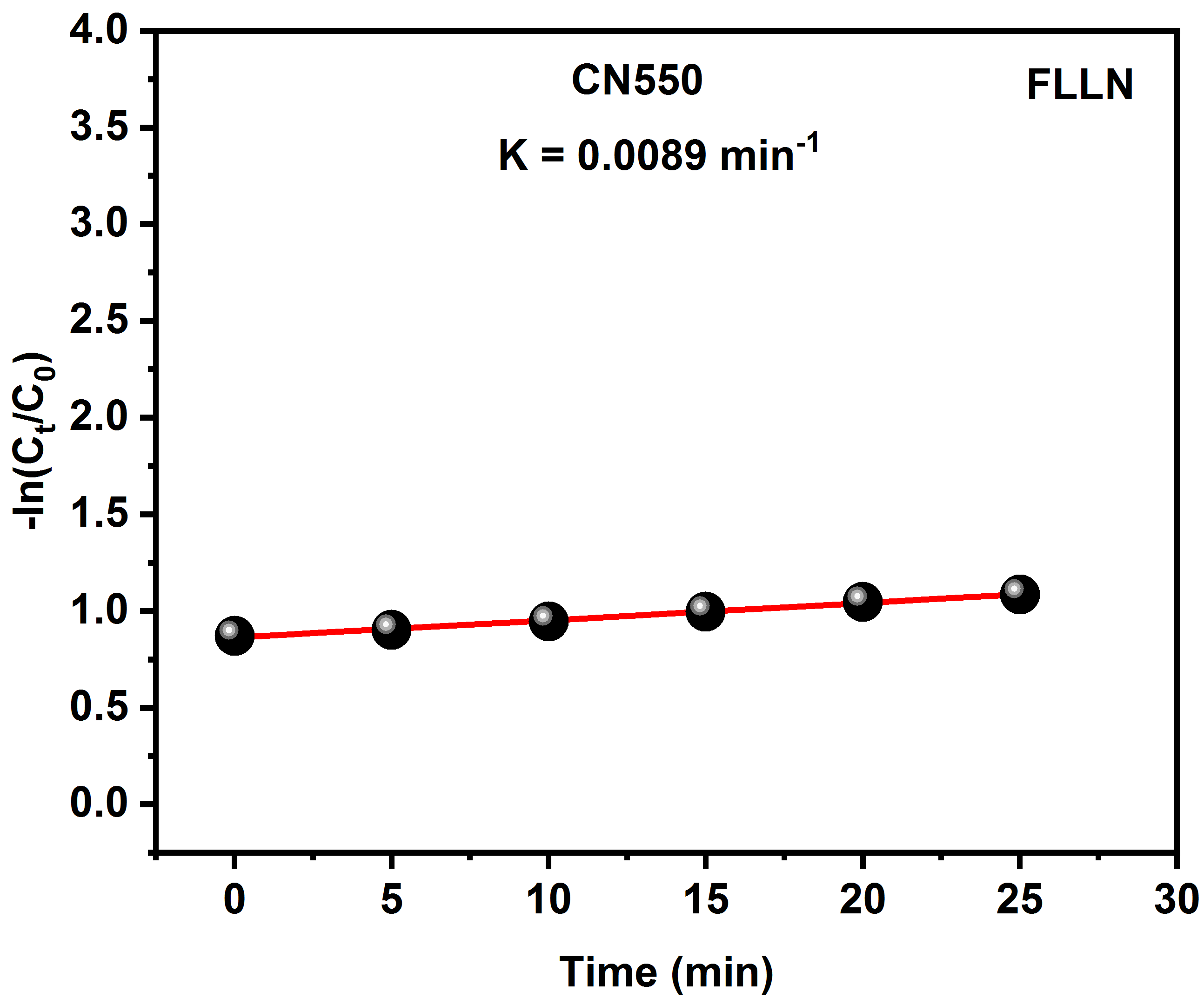

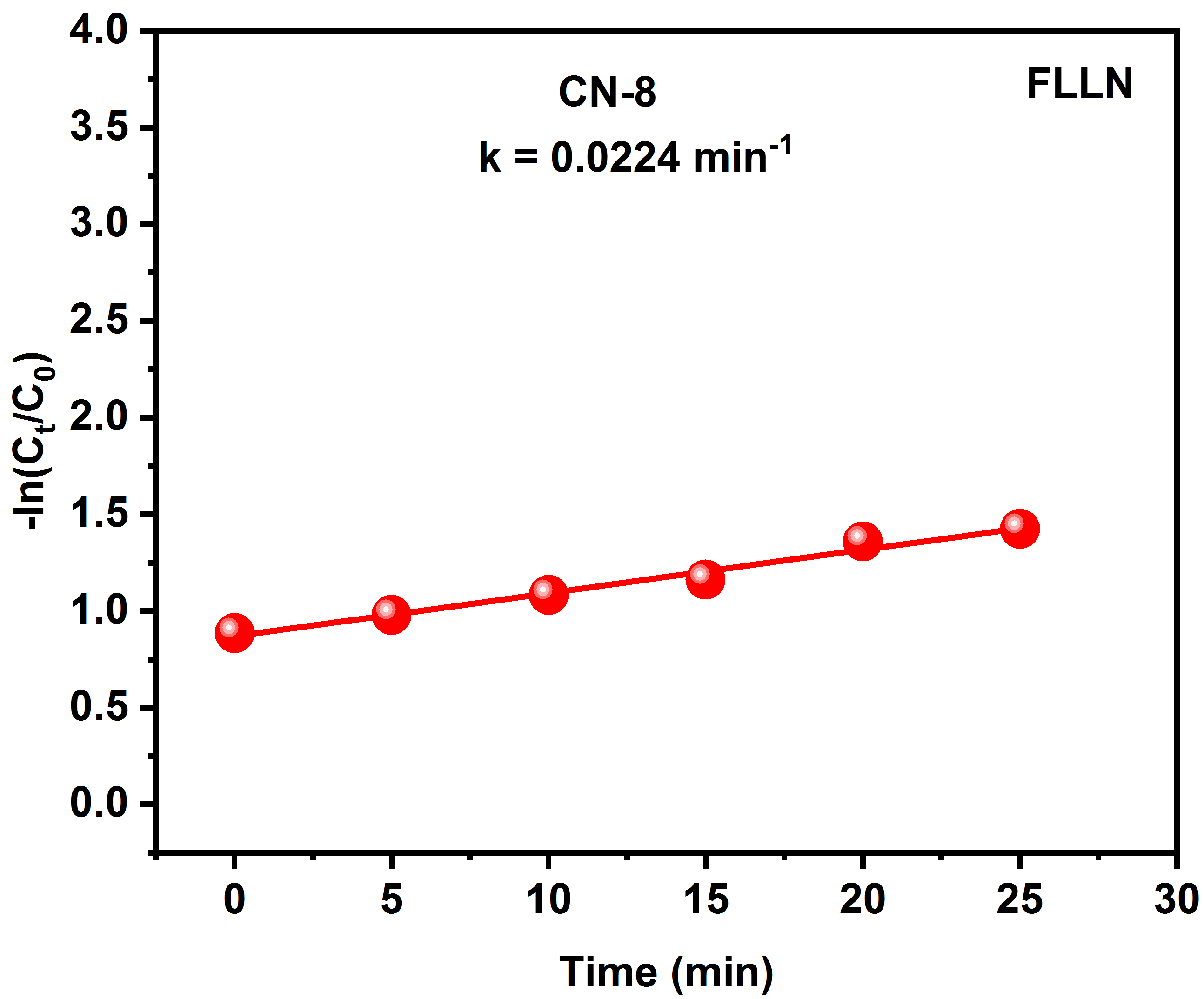

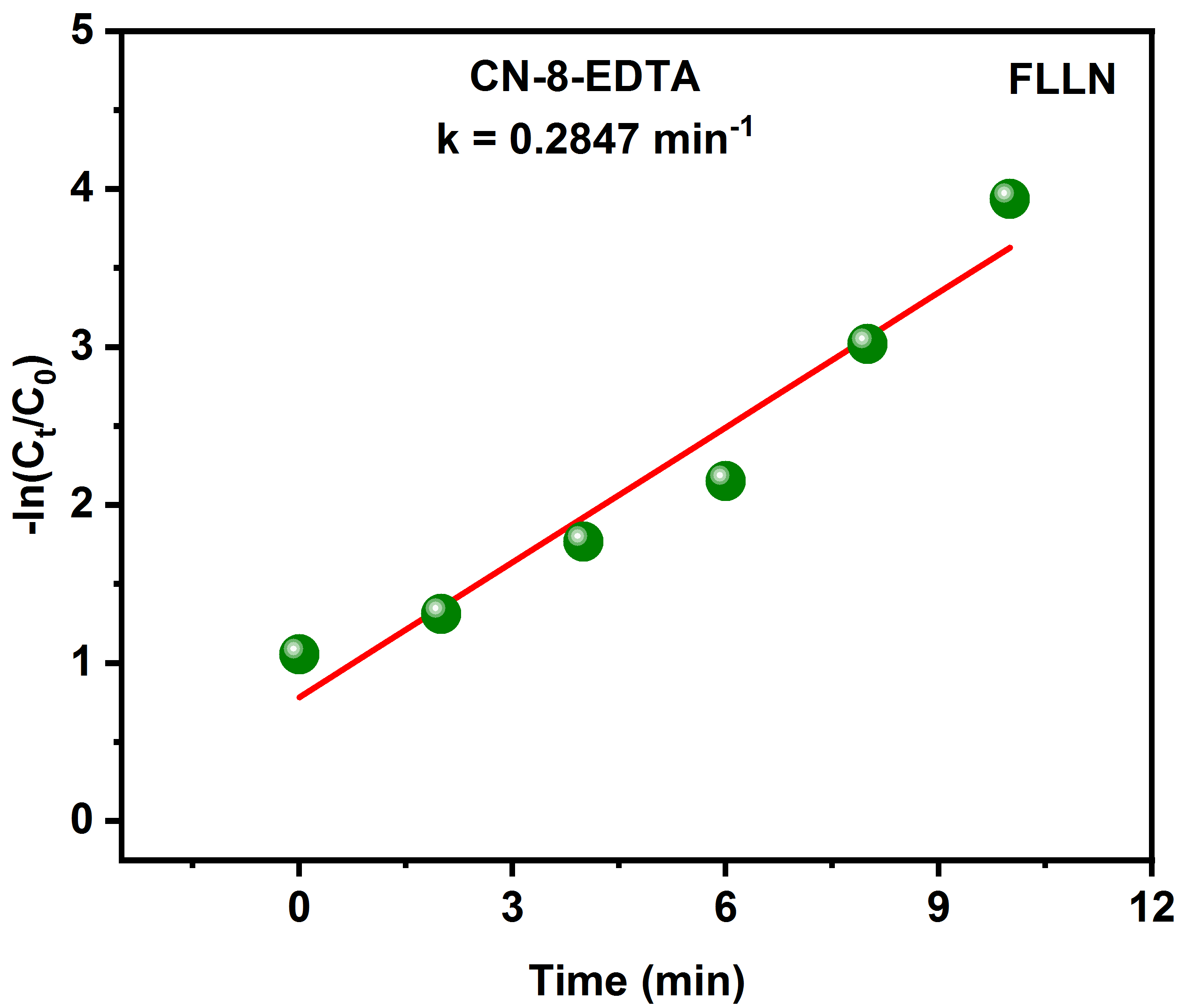


**Figure S18.** The degradation rate of FLLN is iteratively fitted until at least 95% of the data matches the model (R^2^ > 0.95).

**Figure S19.** Possible degradation pathways of FLLN inferred from LC-MS analysis.


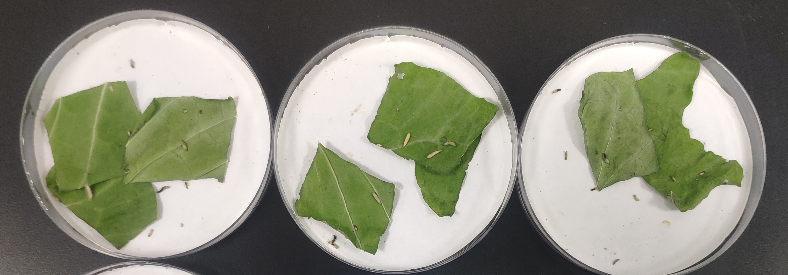

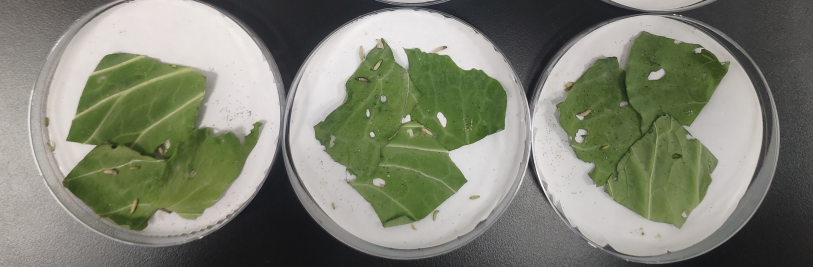


**a**

**b**

**Figure S20.** Testing of toxicity against the diamondback moth using immersion method: (a) solution of 20 mg/L FLLN, (b) solution after 60 minutes of photocatalytic degradation.


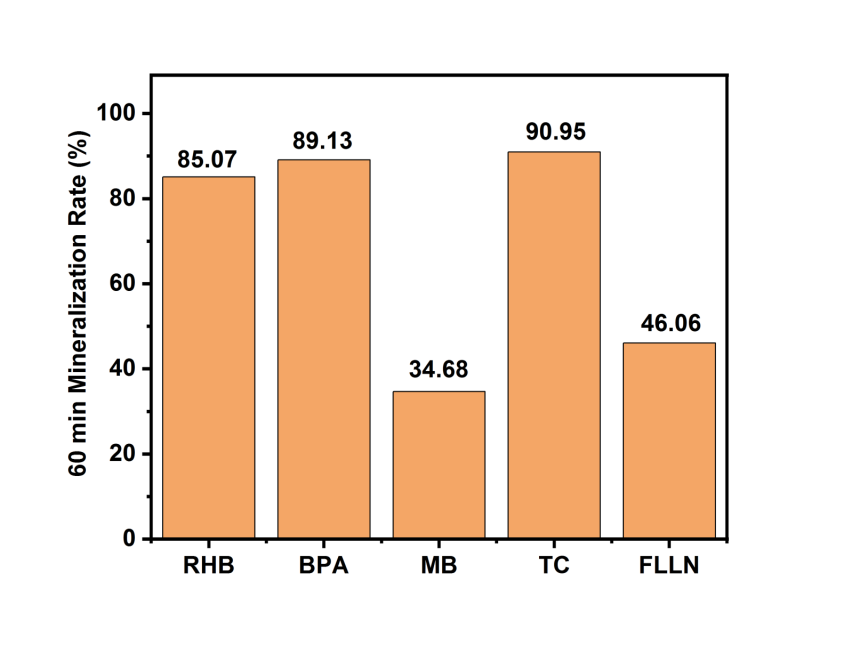


**Figure S21.** Mineralization rate after 60 minutes of 450 nm 12W LED light exposure.


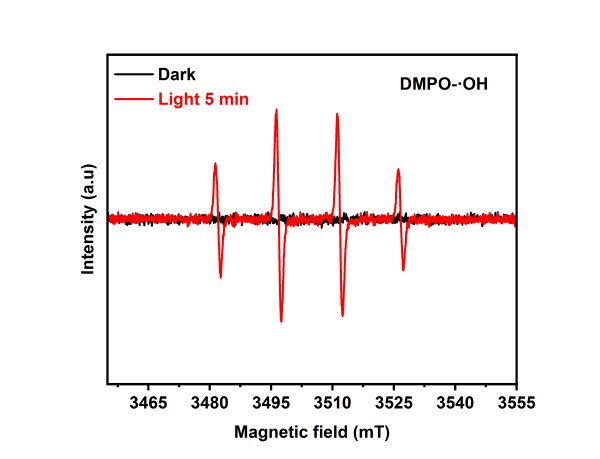


**a**

**b**

**Figure S22.** EPR of **·**OH and e^-^ under dark and light conditions.

**Figure S23.** Rate of degradation over 5 cycles

**Figure S24.** XRD comparison between original CN-8 and CN-8 after 5 cycles.

**Figure S25.** FT-IR comparison of CN-8 after 5 cycles.

**a**

**b**

**c**

**d**

**Figure S26.** (a-b) Original CN-8 (c-d) CN-8 after 5 cycles

**Supplementary Tables**

**Table S1.** Surface compositions of the CN550, CN550-8, CN-8 obtained from the XPS analysis.

| **Sample** | **C 1 s %** | **N 1 s %** | **O 1 s %** | **N-C=N % (C 1 s)** | **N-(C)_2_ % (N 1 s)** |
| --- | --- | --- | --- | --- | --- |
| CN550 | 40.94 | 56.48 | 2.58 | 28.78 | 13.03 |
| CN550-8 | 44.12 | 52.34 | 3.54 | 29.61 | 17.34 |
| CN-8 | 42.85 | 54.59 | 2.56 | 36.49 | 30.11 |

**Table S2.** BET surface area, average pore size, and pore volume of CN520, CN550, and CN-8.

| **Sample** | **BET surface area (m^2^/g)** | **average pore size (nm)** | **pore volume (cm^3^/g)** |
| --- | --- | --- | --- |
| CN520 | 50.55 | 9.118 | 0.1152 |
| CN550 | 77.79 | 11.305 | 0.2171 |
| CN-8 | 92.07 | 12.097 | 0.2785 |

**Table S3.** Comparison of the contribution of Fragment 1 to the first excited state

electron holes and its degradation rate of RhB.

| **Catalyst** | **Fragment 1**  **Holes and Electrons (%)** | **Photocatalytic**  **degradation RhB** |
| --- | --- | --- |
| CN-1 | 13.84 | k = 0.1173 min^-1^ |
| CN-2 | 13.24 | k = 0.0886 min^-1^ |
| CN-3 | 15.56 | k = 0.0672 min^-1^ |
| CN-4 | 13.80 | k = 0.0501 min^-1^ |
| CN-5 | 4.80 | k = 0.0397 min^-1^ |
| CN-6 | 14.47 | k = 0.0920 min^-1^ |
| CN-7 | 16.00 | k = 0.1051 min^-1^ |
| CN-8 | 29.10 | k = 0.2109 min^-1^ |
| CN-9 | 33.32 | k = 0.1013 min^-1^ |
| CN-10 | 19.51 | k = 0.0949 min^-1^ |
| CN-11 | 17.80 | k = 0.0643 min^-1^ |
| CN-12 | 13.36 | k = 0.0685 min^-1^ |

**Table S4.** DFT calculation of excitation energy

| **Catalyst** | **Excitation State 1 (eV)** | **Excitation State 2 (eV)** | **Excitation State 3 (eV)** | **Excitation State 4 (eV)** | **Excitation State 5 (eV)** |
| --- | --- | --- | --- | --- | --- |
| CN-01 | 3.133 | 3.445 | 3.519 | 3.993 | 3.970 |
| CN-02 | 3.187 | 3.418 | 3.531 | 3.899 | 3.952 |
| CN-03 | 3.161 | 3.473 | 3.564 | 3.567 | 3.836 |
| CN-04 | 3.158 | 3.413 | 3.525 | 3.918 | 3.943 |
| CN-05 | 3.353 | 3.418 | 3.542 | 3.561 | 3.967 |
| CN-06 | 3.099 | 3.436 | 3.442 | 3.512 | 3.816 |
| CN-07 | 3.115 | 3.474 | 3.568 | 3.579 | 3.775 |
| CN-08 | 3.113 | 3.409 | 3.488 | 3.516 | 3.847 |
| CN-09 | 3.043 | 3.403 | 3.437 | 3.505 | 3.713 |
| CN-10 | 3.096 | 3.407 | 3.423 | 3.512 | 3.842 |
| CN-11 | 3.100 | 3.411 | 3.442 | 3.510 | 3.864 |
| CN-12 | 3.166 | 3.413 | 3.525 | 3.873 | 3.943 |

**Table S5.** Excitation State 1 contribution of each fragment to holes and electrons

| **Catalyst** | **Fragment 1**  **Holes(%)** | **Fragment 1**  **Electrons(%)** | **Fragment 2**  **Holes(%)** | **Fragment 2**  **Electrons(%)** | **Fragment 3**  **Holes(%)** | **Fragment 3**  **Electrons(%)** |
| --- | --- | --- | --- | --- | --- | --- |
| CN-01 | 0.98 | 12.86 | 92.97 | 77.21 | 6.21 | 9.83 |
| CN-02 | 1.30 | 11.94 | 92.34 | 76.88 | 6.36 | 11.18 |
| CN-03 | 0.88 | 14.68 | 93.67 | 75.74 | 5.44 | 9.58 |
| CN-04 | 0.86 | 12.94 | 92.34 | 72.45 | 6.81 | 14.61 |
| CN-05 | 0.89 | 3.91 | 77.97 | 67.16 | 21.13 | 28.85 |
| CN-06 | 0.82 | 13.65 | 92.42 | 70.63 | 6.75 | 15.72 |
| CN-07 | 1.00 | 15.00 | 93.08 | 69.90 | 5.91 | 15.10 |
| CN-08 | 13.46 | 15.64 | 79.55 | 67.54 | 6.99 | 16.82 |
| CN-09 | 12.86 | 20.46 | 80.56 | 61.16 | 6.58 | 18.38 |
| CN-10 | 1.16 | 18.35 | 92.80 | 72.36 | 6.04 | 9.29 |
| CN-11 | 0.98 | 16.82 | 92.84 | 74.27 | 6.18 | 8.91 |
| CN-12 | 0.90 | 12.46 | 92.64 | 76.82 | 6.46 | 10.72 |

**Table S6.** Comparison of photocatalytic RhB properties of g-C_3_N_4_-based photocatalyst.

| **Photocatalyst** | **Light source** | **Degradation rate**  **performance** | **(Ref.)** |
| --- | --- | --- | --- |
| Ag/FeWO_4_/g-C_3_N_4_ | sunlight illumination | 98% degradation after 120 min | (Saher et al., 2020) |
| g-C_3_N_4_@NiFe-LDH | 500 W Xe lamp, λ ≥ 420 nm | 99% degradation after 240 min | (He et al., 2021) |
| 2D g-C_3_N_4_ nanoflakes | 150 W Xe lamp, λ ≥ 420 nm | 90% degradation after 180 min | (Hu et al., 2021) |
| poly(tannic acid)/g-C_3_N_4_ | 500 W Xe lamp, λ ≥ 420 nm | 97% degradation after 80 min | (Yang et al., 2021) |
| g-C_3_N_4_ nanosheets | 300 W Xe lamp, λ ≥ 420 nm | k = 0.3513 min^-1^ | (Linh et al., 2021) |
| 2TCOH-30 %/CN-N_0.02_ | 300 W Xe lamp, λ ≥ 420 nm | 98.3% degradation after 15 min | (Yang et al., 2024) |
| 3-P/K-CN-N | light illumination, λ ≥ 420 nm | k = 0.10601 min^-1^ | (Sun et al., 2024) |
| CN-8-EDTA | 6 W LED, λ = 450 nm | k = 0.6353 min^-1^ | This work |

**Table S7.** Comparison of photocatalytic MB properties of g-C_3_N_4_-based photocatalyst.

| **Photocatalyst** | **Light source** | **Degradation rate**  **performance** | **(Ref.)** |
| --- | --- | --- | --- |
| BaTiO_3_/g-C_3_N_4_ | sunlight irradiation | 91% degradation after 90 min | (Kappadan, Thomas and Kalarikkal, 2021) |
| g-C_3_N_4_@BiOCl | sunlight irradiation | k = 0.0301 min^-1^ | (Kundu, Sharma and Basu, 2021) |
| g-C_3_N_4_/BaTiO_3_ | 75 W-220 V lamp, λ > 400 nm | k = 0.0103 min^-1^ | (Nguyen et al., 2021) |
| g-C_3_N_4_/Ni-ZnO | sunlight irradiation | 89.45% degradation after 90 min | (Qamar et al., 2021) |
| g-C_3_N_4_/Gd_2_O_3_ | 300 W Xe lamp, λ ≥ 420 nm | 100% degradation after 120 min | (Zhou et al., 2021b) |
| Fe_3_O_4_/g-C_3_N_4_ | LED 50 W Philips light，mixture | k = 0.031 min^-1^ | (Singh Shagolsem and Mohondas Singh, 2024) |
| C_3_N_4_@Co_3_V_2_O_8_ | 300 W Xe lamp, λ ≥ 420 nm | 93.7% degradation after 60 min | (Cao et al., 2025) |
| CN-8 | 12 W LED, λ = 450 nm | k = 0.0731 min^-1^ | This work |

**Table S8.** Comparison of photocatalytic TC properties of g-C_3_N_4_-based photocatalyst.

| **Photocatalyst** | **Light source** | **Degradation rate**  **performance** | **(Ref.)** |
| --- | --- | --- | --- |
| N-TiO_2_/CNO_NV_-2 | visible light, λ ≥ 420 nm | k = 0.0169 min^-1^ | (Wang et al., 2020) |
| Er/g-C_3_N_4_ | 35 W Xe lamp, visible light | k = 0.0117 min^-1^ | (Li et al., 2020) |
| g-C_3_N_4_/Na-BiVO_4_ | 300 W Xe lamp, λ ≥ 420 nm | 98.20% degradation after 40 min | (Kang et al., 2021) |
| ZnSnO_3_@g-C_3_N_4_ | visible light irradiation | k = 0.0190 min^-1^ | (Zhu et al., 2020) |
| CuWO_4_/g-C_3_N_4_ | 300 W halogen lamp, visible light | 88% degradation after 120 min | (Vinesh et al., 2022) |
| Pd@BC/g-C_3_N_4_ | 300 W EP S3, Sonica, Italy | 94.23% degradation after 120 min | (Yekan Motlagh et al., 2024) |
| Fe-CNS | ~ | k = 0.04729 min^-1^ | (Tian et al., 2024) |
| CN-8-EDTA | 12 W LED, λ = 450 nm | k = 0.1947 min^-1^ | This work |

**Table S9.** Comparison of photocatalytic BPA properties of g-C_3_N_4_-based photocatalyst.

| **Photocatalyst** | **Light source** | **Degradation rate**  **performance** | **(Ref.)** |
| --- | --- | --- | --- |
| g-C_3_N_4_/ZIF-8/AgBr | 300 W Xe lamp, λ ≥ 420 nm | k = 0.0117 min^-1^ | (Zheng et al., 2021) |
| CeO_2_/g-C_3_N_4_ | visible light irradiation | k = 0.0197 min^-1^ | (Zhao et al., 2021) |
| g-C_3_N_4_-CA-0.3 | 300 W Xe lamp, λ ≥ 420 nm | k = 0.0203 min^-1^ | (Zhou et al., 2021a) |
| g-C_3_N_4_/BiOI/CdS | 300 W Xe lamp, λ ≥ 420 nm | 98.62% degradation after 120 min | (Zhang et al., 2022) |
| Bi_2_WO_6_/g-C_3_N_4_/BPQDs | 300 W Xe lamp, λ ≥ 380 nm | 95.6% degradation after 120 min | (Du et al., 2023) |
| GdFeO_3_/NiO@g-C_3_N_4_ | visible light source | 94.23% degradation after 45 min | (Iqbal et al., 2024) |
| g-C_3_N_4_/Ag_3_PO_4_ | 300 W Xe lamp, λ ≥ 420 nm | k = 0.0419 min^-1^ | (Li and Xie, 2024) |
| CN-8-EDTA | 12 W LED, λ = 450 nm | k = 0.1993 min^-1^ | This work |

**FLLN LC-MS analysis**

1.

2. 3.

4.

5. 6.


**References**

Cao, X., Liu, G., Zheng, J., Sui, Z., Zheng, S. and Zhang, Q. 2025. Ultra-thin g-C3N4-Modified Co3V2O8 hollow spheres for enhanced photocatalytic degradation of MB. Solid State Sciences 160.

Du, F., Lai, Z., Tang, H., Wang, H. and Zhao, C. 2023. Construction of dual Z-scheme Bi(2)WO(6)/g-C(3)N(4)/black phosphorus quantum dots composites for effective bisphenol A degradation. J Environ Sci (China) 124, 617-629.

He, Y., Zhou, S., Wang, Y., Jiang, G. and Jiao, F. 2021. Fabrication of g-C3N4@NiFe-LDH heterostructured nanocomposites for highly efficient photocatalytic removal of rhodamine B. Journal of Materials Science: Materials in Electronics 32(17), 21880-21896.

Hu, C., Liu, Z.-T., Yang, P.-C., Ding, Y.-X., Lin, K.-Y.A. and Nguyen, B.-S. 2021. Self-assembly L-cysteine based 2D g-C3N4 nanoflakes for light-dependent degradation of rhodamine B and tetracycline through photocatalysis. Journal of the Taiwan Institute of Chemical Engineers 123, 219-227.

Iqbal, S., Bibi, F., Taha, S.S., Mohany, M., Iqbal, R., Kalsoom, A., Ahmad, K., ahmed, A. and Jamshaid, M. 2024. A versatile GdFeO3/NiO@g-C3N4 ternary hetero-structure photo catalyst for effective photo-degradation and adsorption of tetracycline and ciprofloxacin from wastewater. Synthetic Metals 309.

Kang, J., Tang, Y., Wang, M., Jin, C., Liu, J., Li, S., Li, Z. and Zhu, J. 2021. The enhanced peroxymonosulfate-assisted photocatalytic degradation of tetracycline under visible light by g-C3N4/Na-BiVO4 heterojunction catalyst and its mechanism. Journal of Environmental Chemical Engineering 9(4).

Kappadan, S., Thomas, S. and Kalarikkal, N. 2021. Enhanced photocatalytic performance of BaTiO3/g-C3N4 heterojunction for the degradation of organic pollutants. Chemical Physics Letters 771.

Kundu, A., Sharma, S. and Basu, S. 2021. Modulated BiOCl nanoplates with porous g-C3N4 nanosheets for photocatalytic degradation of color/colorless pollutants in natural sunlight. Journal of Physics and Chemistry of Solids 154.

Li, G., Wang, B., Zhang, J., Wang, R. and Liu, H. 2020. Er-doped g-C3N4 for photodegradation of tetracycline and tylosin: High photocatalytic activity and low leaching toxicity. Chemical Engineering Journal 391.

Li, J.-X. and Xie, Y.-L. 2024. Enhanced separation performance of g-C3N4/Ag3PO4 heterojunction photocatalyst for efficient degradation of methylene blue and bisphenol A. Journal of Alloys and Compounds 1005.

Linh, P.H., Do Chung, P., Van Khien, N., Oanh, L.T.M., Thu, V.T., Bach, T.N., Hang, L.T., Hung, N.M. and Lam, V.D. 2021. A simple approach for controlling the morphology of g-C3N4 nanosheets with enhanced photocatalytic properties. Diamond and Related Materials 111.

Nguyen, V.K., Nguyen Thi, V.N., Tran, H.H., Tran Thi, T.P., Truong, T.T. and Vo, V. 2021. A facile synthesis of g-C3N4/BaTiO3 photocatalyst with enhanced activity for degradation of methylene blue under visible light. Bulletin of Materials Science 44(1).

Qamar, M.A., Shahid, S., Javed, M., Iqbal, S., Sher, M., Bahadur, A., Al-Anazy, M.M., Laref, A. and Li, D. 2021. Designing of highly active g-C3N4/Ni-ZnO photocatalyst nanocomposite for the disinfection and degradation of the organic dye under sunlight radiations. Colloids and Surfaces A: Physicochemical and Engineering Aspects 614.

Saher, R., Hanif, M.A., Mansha, A., Javed, H.M.A., Zahid, M., Nadeem, N., Mustafa, G., Shaheen, A. and Riaz, O. 2020. Sunlight-driven photocatalytic degradation of rhodamine B dye by Ag/FeWO4/g-C3N4 composites. International Journal of Environmental Science and Technology 18(4), 927-938.

Singh Shagolsem, B. and Mohondas Singh, N. 2024. An environmentally friendly photocatalyst for the breakdown of RhB, MB, 2,4-DCP, and TCAA: Reusable Fe3O4/Ag doped g-C3N4. Journal of Molecular Liquids 413.

Sun, Z., Li, J., Qiu, X., Wang, K. and Guo, L. 2024. One-step thermal polymerization synthesis of P and K co-doped two-dimensional porous g-C3N4 photocatalyst with enhanced visible light photocatalytic activity for RhB. Materials Letters 370.

Tian, X., Lin, Y., Xu, H., Gao, X., Wu, W., Wang, A., Zhang, X., Wang, J. and Yao, W. 2024. Construction of 2D porous g-C3N4 by steam reforming with the introduction of Fe clusters forming Fe-Nx to enhance photo-Fenton catalytic activity. Journal of Water Process Engineering 66.

Vinesh, V., Preeyanghaa, M., Kumar, T.R.N., Ashokkumar, M., Bianchi, C.L. and Neppolian, B. 2022. Revealing the stability of CuWO(4)/g-C(3)N(4) nanocomposite for photocatalytic tetracycline degradation from the aqueous environment and DFT analysis. Environ Res 207, 112112.

Wang, Y., Rao, L., Wang, P., Shi, Z. and Zhang, L. 2020. Photocatalytic activity of N-TiO2/O-doped N vacancy g-C3N4 and the intermediates toxicity evaluation under tetracycline hydrochloride and Cr(VI) coexistence environment. Applied Catalysis B: Environmental 262.

Yang, B., Wang, C., Ji, X., Li, G., Mao, J., Zhang, H. and Yang, Y. 2024. Decorating nitrogen-deficient crystalline g-C3N4 with Ti3C2(OH)2 for photocatalytic CO2 reduction and RhB degradation enhancement. Journal of Alloys and Compounds 1008.

Yang, D., Wang, W., An, K., Chen, Y., Zhao, Z., Gao, Y. and Jiang, Z. 2021. Bioinspired construction of carbonized poly(tannic acid)/g-C3N4 nanorod photocatalysts for organics degradation. Applied Surface Science 562.

Yekan Motlagh, P., Vahid, B., Babazadeh, N., Karimpour, D., Kayan, B., Baran, T., Yoon, Y. and Khataee, A. 2024. Palladium nanoparticles supported on biochar/graphitic carbon nitride as a heterogeneous catalyst for pharmaceutical degradation. Journal of Environmental Chemical Engineering 12(4).

Zhang, Y., Wu, Y., Wan, L., Yang, W., Ding, H., Lu, C., Zhang, W. and Xing, Z. 2022. Double Z-Scheme g-C3N4/BiOI/CdS heterojunction with I3−/I− pairs for enhanced visible light photocatalytic performance. Green Energy & Environment 7(6), 1377-1389.

Zhao, W., She, T., Zhang, J., Wang, G., Zhang, S., Wei, W., Yang, G., Zhang, L., Xia, D., Cheng, Z., Huang, H. and Leung, D.Y.C. 2021. A novel Z-scheme CeO2/g-C3N4 heterojunction photocatalyst for degradation of Bisphenol A and hydrogen evolution and insight of the photocatalysis mechanism. Journal of Materials Science & Technology 85, 18-29.

Zheng, W., Feng, S., Feng, S., Shao, C., Jiang, Z., Wu, W., Ge, Y., Liao, S., Li, K., Duan, C. and Meng, Q. 2021. The g-C3N4 modified by AgBr and ZIF-8 adsorption-photocatalysis synergistic degradation of bisphenol A. Research on Chemical Intermediates 47(4), 1471-1487.

Zhou, M., Jing, L., Dong, M., Lan, Y., Xu, Y., Wei, W., Wang, D., Xue, Z., Jiang, D. and Xie, J. 2021a. Novel broad-spectrum-driven g-C(3)N(4) with oxygen-linked band and porous defect for photodegradation of bisphenol A, 2-mercaptophenthiazole and ciprofloxacin. Chemosphere 268, 128839.

Zhou, Y., Zeng, F., Sun, C., Wu, J., Xie, Y., Zhang, F., Rao, S., Wang, F., Zhang, J., Zhao, J. and Li, S. 2021b. Gd2O3 nanoparticles modified g-C3N4 with enhanced photocatalysis activity for degradation of organic pollutants. Journal of Rare Earths 39(11), 1353-1361.

Zhu, X., Guo, F., Pan, J., Sun, H., Gao, L., Deng, J., Zhu, X. and Shi, W. 2020. Fabrication of visible-light-response face-contact ZnSnO3@g-C3N4 core–shell heterojunction for highly efficient photocatalytic degradation of tetracycline contaminant and mechanism insight. Journal of Materials Science 56(6), 4366-4379.
